# Test-retest Repeatability and Interobserver Variation of Healthy Tissue Metabolism using FDG-PET/CT of the Thorax among Lung Cancer Patients

**DOI:** 10.1097/MNM.0000000000001537

**Published:** 2022-05-01

**Authors:** Afnan A. Malaih, Joel T. Dunn, Lotte Nygård, David G. Kovacs, Flemming L. Andersen, Sally F. Barrington, Barbara M. Fischer

**Affiliations:** 1 School of Biomedical Engineering and Imaging Sciences, PET Imaging Centre, St Thomas Hospital, King’s College London, London SE1 7EH, UK; 2 Department of Oncology, Rigshospitalet, Copenhagen University Hospital, Blegdamsvej 9, 2100 Copenhagen, Denmark; 3 Department of Clinical Physiology, Nuclear Medicine and PET, Rigshospitalet, University of Copenhagen, Blegdamsvej 9, 2100 Copenhagen, Denmark

**Keywords:** ^18^F-FDG, PET-CT, thorax, standardized uptake value, healthy tissues, repeatability, interobserver variation

## Abstract

**Objectives:**

The aim of this study was to assess the test-retest repeatability and interobserver variation in healthy tissue (HT) metabolism using 2-deoxy-2-[^18^F]fluoro-D-glucose (^18^F-FDG) positron emission tomography/computed tomography (PET/CT) of the thorax in lung cancer patients.

**Methods:**

A retrospective analysis was conducted in 22 patients with non-small cell lung cancer who had two PET/CT scans of the thorax performed three days apart with no interval treatment. The maximum, mean and peak standardized uptake values (SUV) in different HTs were measured by a single observer for the test-retest analysis and two observers for interobserver variation. Bland-Altman plots were used to assess the repeatability and interobserver variation. Intrasubject variability was evaluated using within-subject coefficients of variation (wCV).

**Results:**

The wCV of test-retest SUV_mean_ measurements in mediastinal blood pool, bone marrow, skeletal muscles and lungs were <20%. The left ventricle (LV) showed higher wCV (>60%) in all SUV parameters with wide limits of repeatability. High interobserver agreement was found with wCV of <10% in SUV_mean_ of all HT, but up to 22% was noted in the LV.

**Conclusion:**

HT metabolism is stable in a test-retest scenario and has high interobserver agreement. SUVmean was the most stable metric in organs with low FDG-uptake and SUVpeak in HTs with moderate uptake. Test-retest measurements in LV were highly variable irrespective of the SUV parameters used for measurements.

## Introduction

The uptake of 2-deoxy-2-[^18^F]fluoro-D-glucose (^18^F-FDG) reflects glucose metabolism, not only in inflammatory and malignant tissues, but also in healthy tissues (HT) such as lung, myocardium, liver, spleen and bone marrow. Several studies have reported ^18^F-FDG uptake using positron emission tomography/computed tomography (PET/CT) in HT either before or during treatment to be linked to different factors including potential treatment-related adverse events [1–5]. Monitoring cancer response to therapy as well as their effects on HTs has become even more relevant with the increasing use of immunotherapy. Immunotherapy, such as the immune check-point inhibitors, used to treat various solid tumours and some haematological malignancies can activate the immune system causing unique patterns of ^18^F-FDG uptake in tumour as well as HT reflecting the induced inflammatory response [6,7]. These patterns in conjunction with changes in tumour metabolism may predict response to treatment or indicate treatment-related adverse events. Thus, expanding the utilization of ^18^F-FDG PET/CT to evaluate not only tumour but also healthy tissue metabolism during treatment may have potential to predict side effects and improve management of oncology patients.

As ^18^F-FDG distribution in the body is non-specific [8], it is important to distinguish variations due to physiological changes or measurement error from abnormal or true changes in tissue metabolism when assessing cancer patients undergoing different treatment modalities. Few studies in the literature have evaluated variations in HT metabolism [9,10] and most have focused on specific organs such as the liver as a reference organ [11,12] or interventional treatments performed between scans [13–15]. Knowledge of variations in HT metabolism measured by ^18^F-FDG without interval treatment in a test-retest setting is still very limited. Furthermore, consistency in the interpretation and measurement of ^18^F-FDG uptake in HTs between reporters is important, especially as no standardised method for measurement has yet been agreed upon [9-10,14].

The first aim of this study was to evaluate the test-retest repeatability of ^18^F-FDG as a surrogate for HT metabolism in the thoracic area using different standardized uptake value (SUV) parameters commonly applied in PET/CT imaging for cancer patients who received no intervening treatment. The second aim was to assess the interobserver variation in HT metabolism using ^18^F-FDG PET/CT and suggest suitable methods for measurement of HT metabolism in future studies.

## Materials and Methods

### Patients

Data were analysed retrospectively from 22 patients with non-small cell lung cancer (NSCLC) participating in a prospective repeatability study using ^18^F-FDG PET/CT from Rigshospitalet in Copenhagen [16]. For the analysis, areas of interest that were outside of the scanning field of view, showed artefacts or disease involvement were excluded.

All patients gave their informed consent in writing for the scientific use of their data. Study approval was obtained from the Danish Ethics Committee (protocol number H-1-2014-011) and the Danish Data Protection Agency (02986/30-1271) [16]. Study methods were performed in accordance with the relevant guidelines and regulations.

### Image Acquisition

All patients were instructed to fast for at least 4 hours prior to examination. Patients were administered 4 MBq/kg of ^18^F-FDG and positioned in the radiotherapy treatment position for scanning with both arms placed over the head. Two PET/CT scans were performed 2 to 5 days apart with no interval treatment. The scans were aimed to be acquired at the same time of the day. On both days the patients had a thoracic PET scan with low-dose CT on the same PET/CT scanner (Siemens Biograph mCT, Siemens Healthineers Erlangen) following the guidelines of the European Association of Nuclear Medicine (EANM) [16-17].

For PET acquisition, 2 – 3 minutes per bed position was applied for patients according to body mass index (BMI). Iterative reconstruction was used to correct for attenuation and scatter in PET images with 3D-ordered-subset expectation-maximisation technique which involved point spread function and time of flight. PET images were reconstructed with pixel sizes of 2 x 2 mm and slice thickness of 2 mm. Low dose CT scans were acquired in 3 to 4 seconds using 120 kV and 40 mAs and were subsequently used for attenuation correction of PET images. Detailed information about image acquisition and procedure was described in previous published work by Nygård *et al* [16].

### Image Analysis

For assessment of test-retest repeatability using the acquired free breathing PET/CT scans the following healthy tissue regions were evaluated: mediastinal blood pool (MBP), left ventricle (LV), bone marrow (BM), skeletal muscle (SM), lungs divided into right upper zone (RUZ), right middle zone (RMZ), right lower zone (RLZ), left upper zone (LUZ), left middle zone (LMZ) and left lower zone (LLZ). A Mirada XD^®^ workstation version 1.1.0.3.1 (Mirada Medical, Oxford, UK) was used to measure the maximum SUV (SUV_max_), mean SUV (SUV_mean_), and peak SUV (SUV_peak_). SUVs were measured using a 1.5 cm sphere as a volume of interest (VOI) or a manually contoured region of interest (ROI). Evaluation of each organ is described in more details in (Table 1) Table 1. Furthermore as patients included in this study did not adhere to the strict diet recommended for cardiac studies [18] measurement of myocardial uptake was supplemented with a simple visual score (0= no uptake, less than or equal to MBP, 1= patchy uptake above MBP, 2= diffusely increased LV uptake above MBP). All PET/CT scans were evaluated by a single observer (nuclear medicine technologist) for the test-retest repeatability and by two observers for the analysis of inter-observer variation i.e. a nuclear medicine physician (observer 1) with over 10 years experience and the nuclear medicine technologist (observer 2). Both observers analysed all 22 scans, the order of which was randomly selected (scan 1 or scan 2) for each patient. The visual scoring for myocardium was performed by the nuclear medicine physician.

### Statistical Analysis

Descriptive statistics including mean and standard deviation (SD) for all SUV parameters (SUV_max_, SUV_mean_ and SUV_peak_) of each organ on scan 1 and scan 2 were calculated. Scatter plots were created to illustrate the distribution of the SUV parameters in each organ for the test-retest scans. A standard 5% significance level was corrected to *P* <0.0015 using the Bonferroni method with 33 tested parameters to take the multiple comparisons.

Repeatability was defined as the difference between scan 1 and scan 2 in individual patients with the mean ± SD of the differences calculated for each SUV parameter. Further repeatability analysis requires that the difference between the paired observations follow a normal distribution which was assessed with Shapiro-Wilk test [19]. Natural log-transformation was used for the subsequent analysis as SUV measurements tend to be log-normal distributed [19-20]. Paired t-test was used to investigate any significant bias in the differences or log difference. The difference in log-transformed data *d*_ln_ was assessed as follows:

(1) $$d_{\text{ln}}=\text{ln}\left({\text{SUV}}_{2}\right)-\text{ln}\left({\text{SUV}}_{1}\right)$$

where SUV_1_ and SUV_2_ denote SUV from the same organ in scan 1 and scan 2, respectively. To assess the repeatability of a single measurement, the within-subject standard deviation (wSD_ln_) was obtained using the SD of the log-transformed difference and exponentiation was then applied to calculate the within-subject coefficient of variation (wCV%) as a percentage as follows [21]:

(2) $${\text{wSD}}_{\text{ln}}={\text{SD}}_{\text{dln}}/\sqrt{2}$$

(3) $$\text{wCV\%}=\left(\exp\left({\text{wSD}}_{\text{ln}}\right)-1\right)\times 100$$

The 95% repeatability coefficient (RC_ln_) was calculated on the log-transformed data and exponentiation was applied to determine its upper and lower 95% RC in percentage as

(4) $${\text{RC}}_{\text{ln}}=\pm 1.96\times \text{SD}\left(d_{\text{ln}}\right)$$

(5) $$\text{RC}=\left(\exp\left(\pm {\text{RC}}_{\text{ln}}\right)-1\right)\times 100$$

The 95% confidence intervals (CIs) of the upper and lower RCs were also calculated using χ^2^ distribution [21].

Bland-Altman plots were computed for the log-transformed differences against their means with their 95% CIs and the 95% upper and lower limits of RC. Linear regression analysis was used to assess the effect of the mean on the difference which may indicate any proportional bias. Trends of differences against the mean were investigated using Pearson’s Correlation coefficient, on both original and log-transformed data. An additional investigation of trends in variance of differences with mean was assessed using Kendall’s tau to correlate absolute differences against mean in the original and log-transformed data. Similar statistical methods as outlined above were applied for the interobserver analyses. Student paired *t*-test was used to compare the averages in weight, administered activity and uptake time between the two scans. Statistical analyses were performed using IBM SPSS Statistics for Windows, version 26 (IBM Corp., Armonk, N.Y., USA).

## Results

There were 7 female and 15 male patients with histologically confirmed NSCLC all with BMI ≤ 30 and no patients had type I diabetes. Patients characteristics are shown in (Table 2) [16]. In four patients, SUV measurements of the LV were excluded because part of the myocardium was outside the scanning field. SUVs of the lung parenchyma were not measured due to disease involvement in the LUZ, LMZ, LLZ and RUZ in two, two, one and four patients respectively.

### Test-retest Repeatability

We found no significant changes in the average weight, administered activity and uptake time after tracer administration *P* = 0.162, *P* = 0.332 and *P* = 0.719 between the two scans. The paired t-test showed no significant bias in the differences (all *P* >0.0015). Although the t-test can be robust, some violations of assumptions of normal distributions were found, thus, the nonparametric Wilcoxon signed rank test was also applied, though this did not change the results as shown in Table 3 and (see Table S1, supplemental digital content [SDC], which illustrates further repeatability analyses).

**Fig. 1:**
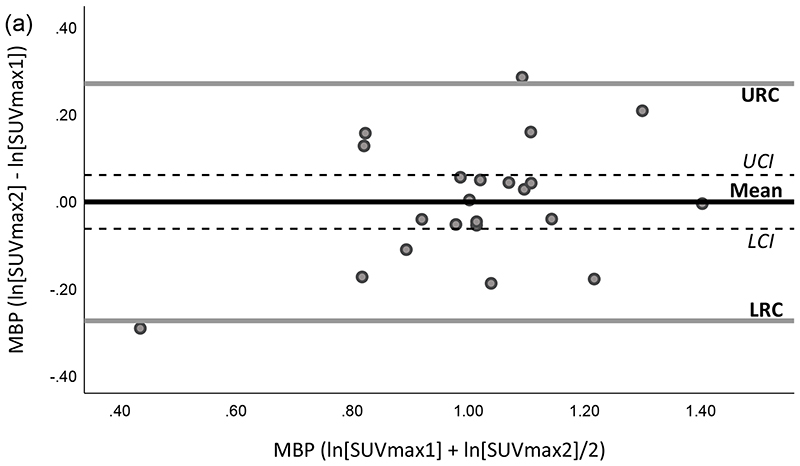

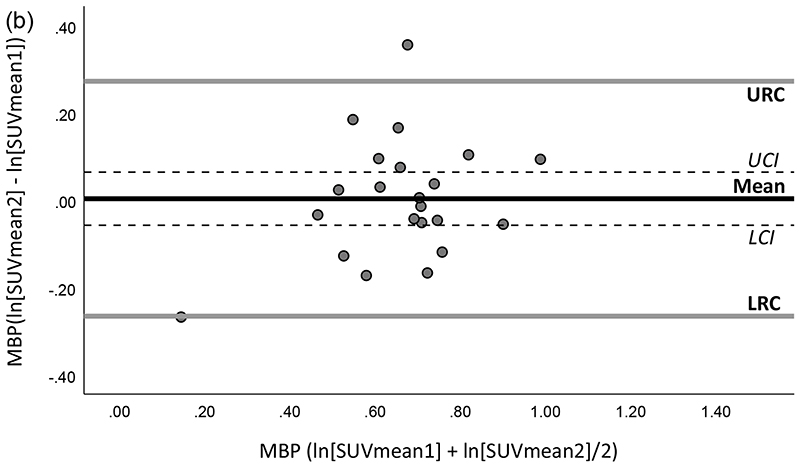

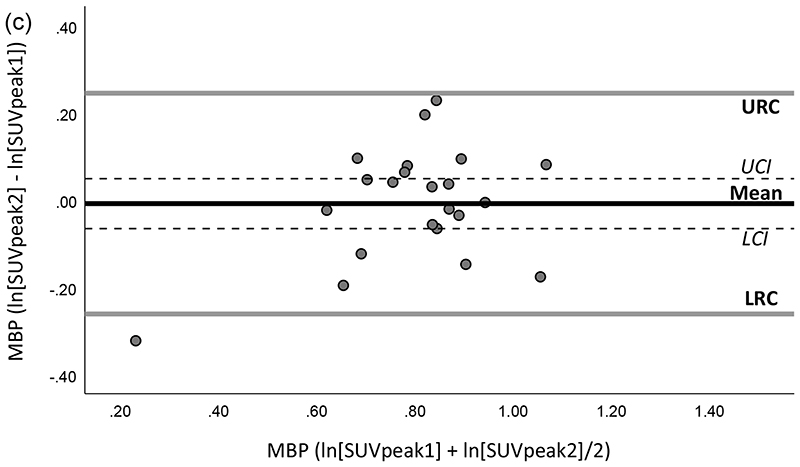

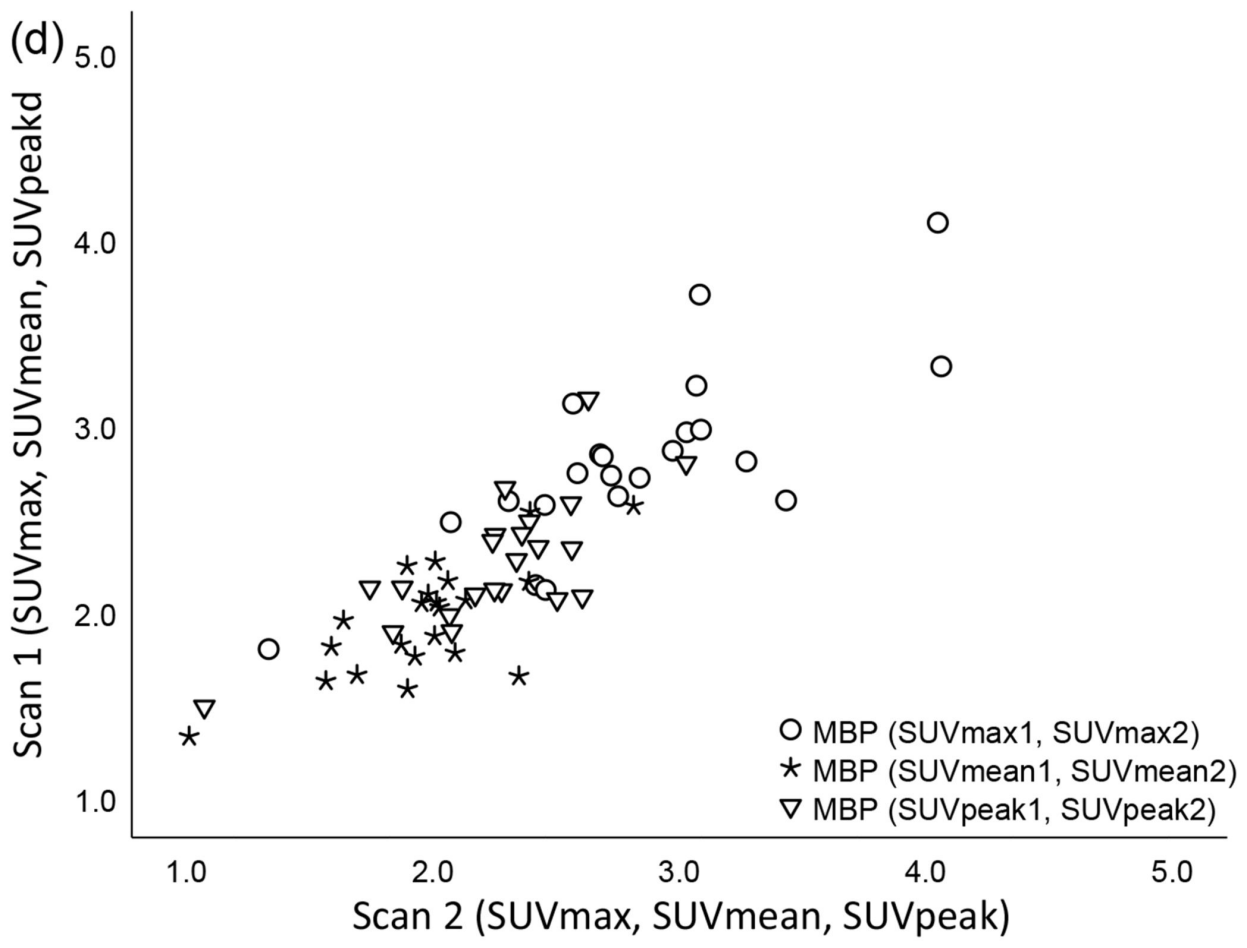

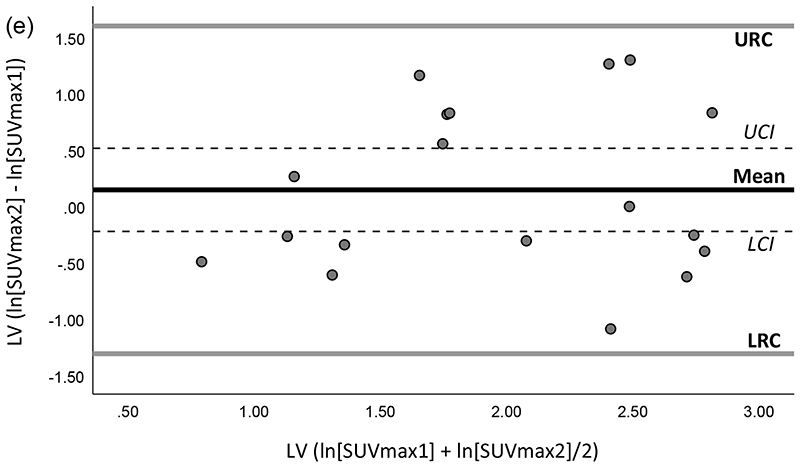

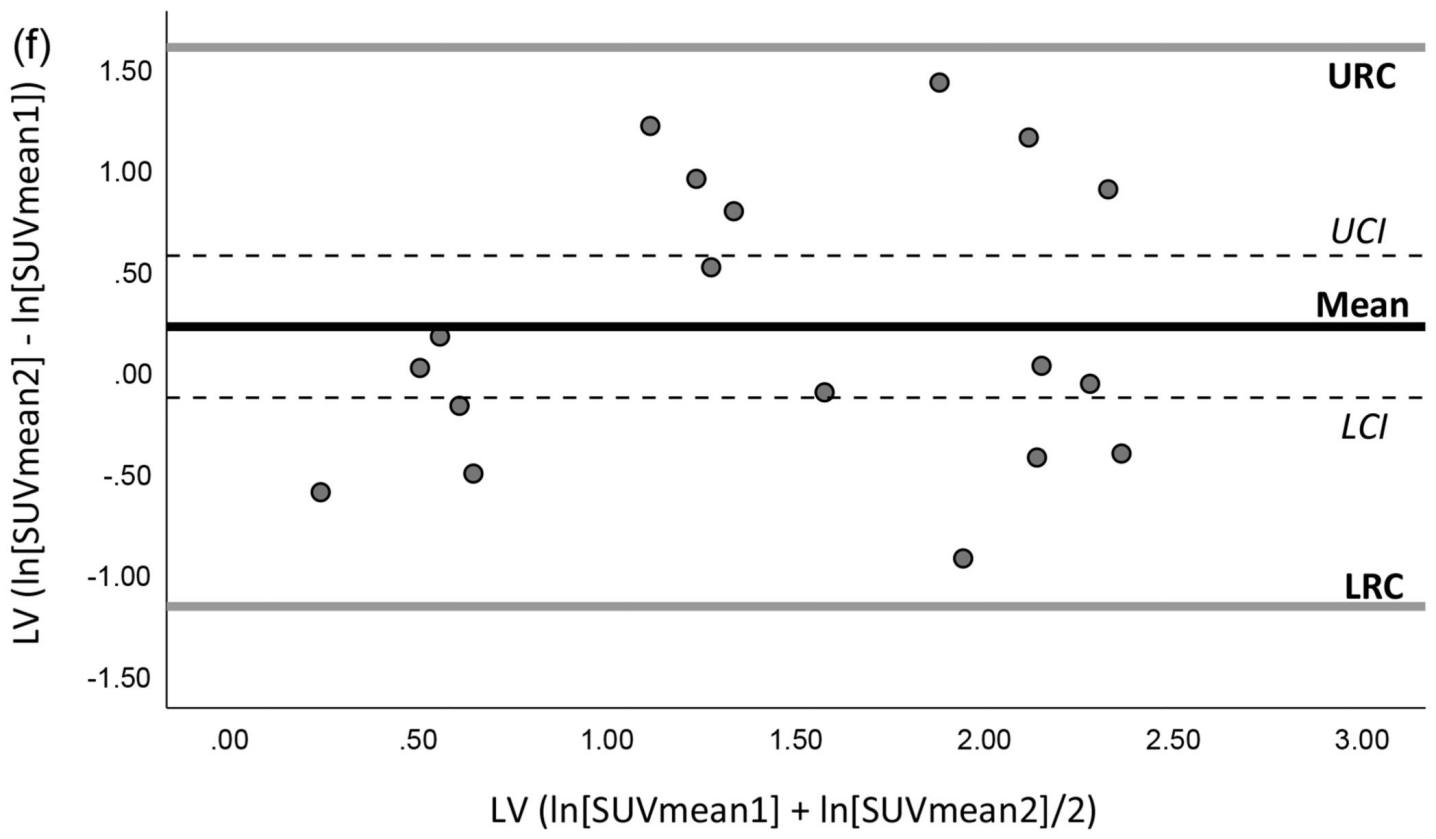

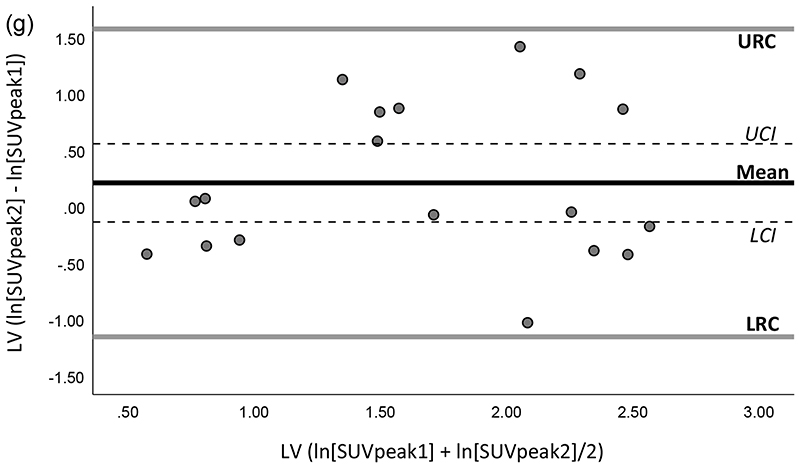

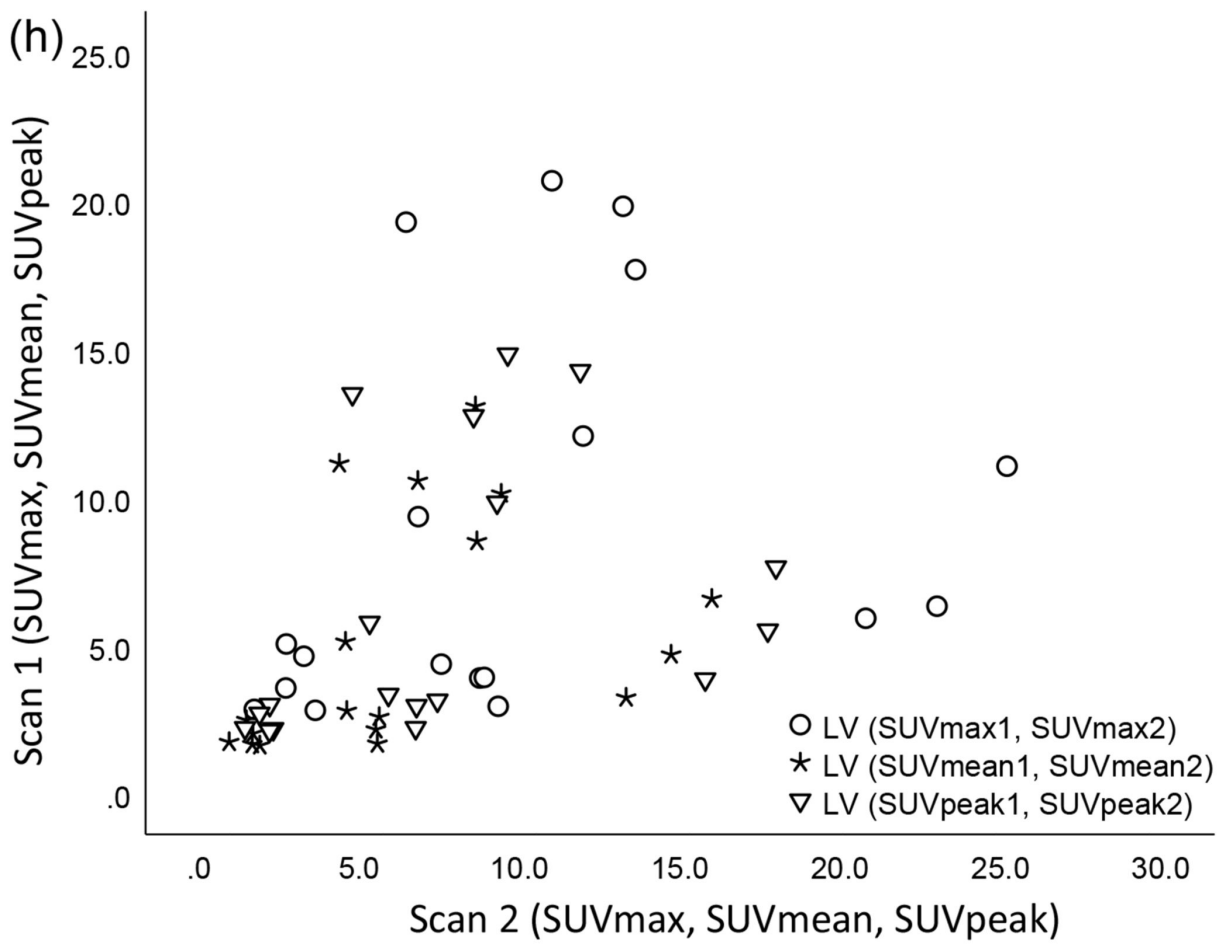
Test-retest repeatability of log-transformed SUV measurements including maximum, mean and peak in (a-c) mediastinal blood pool (MBP) and (e-g) left ventricle (LV) illustrated by the Bland-Altman method. A simple linear regression indicated no significant bias in (a-c) MBP and (e-g) LV data (*P* >0.0015). Scatter plots for distribution of test-retest measurements for different SUV parameters in (d) MBP and (h) LV. Mean, mean of SUV difference between measurements of scan 1 and 2; URC/LRC, upper and lower repeatability coefficients; UCI/LCI, upper and lower 95% confidence intervals of the mean difference; SUV, standardized uptake value.

The mean of the differences for SUV parameters in all HTs between the two scans were small, ranging from -0.13 to +0.11, except in the LV the differences in the means between scans varied from +1.33 to +1.47 (Table 3). Differences in MBP measurements between the two scans had high repeatability with the lowest intra-subject variation, remaining within ~10% and the 95% RCs within -23.9% and +31.4% for all SUV measurements (Table 3) and (Fig. 1). Less stability between the intra-subject measurements were observed in the BM, SM and the lungs assessed by wCV which ranged from 9.9% to 31.3% and corresponded to wider limits of agreement for all SUV parameters as shown in Table 3 (Fig. S1 – S10, SDC, which include Bland-Altman and scatter plots for test-retest of the different tissues). The highest intra-subject variation was associated with measurements in LV with wCVs varying from 63.6% to 69.0% and wide 95% limits of repeatability from -76.7% to +328.3% (Table 3) and (Fig. 1). Based on visual scoring, myocardial uptake similar to MBP (score 1) was seen in 10 patients on scan 1 and 5 on scan 2, uptake higher than MBP with patchy pattern (score 2) in 1 patient on scan 1 and scan 2 and diffuse high uptake (score 3) was seen in 7 patients on scan 1 and 12 on scan 2. All patients with changes in the uptake relative to MBP between the two scans had diffusely increased uptake in the second scan (5 patients from score 1 to 3).

SUV_mean_ measurements based on wCVs were more stable between the two scans compared to SUV_max_ and SUV_peak_ in the lungs. SUV_peak_ was the most stable in MBP and BM with similar stability to SUV_mean_ observed in the SM, but all SUV parameters were highly variable in the LV (Table 3).

Pearson’s Correlation for the difference against the mean showed only a strictly significant trend in SUV_mean_ of the BM with moderate negative correlation in both original and log-transformed data (*r* = -0.64, *P* = 0.001 and *r* = -0.66, *P* <0.001 respectively) (Table S2 and Fig. S1b, SDC, for SUV_mean_ of BM). Pearson’s correlation can be sensitive to violations of assumptions of normality though for completeness the nonparametric Kendall’s tau was also applied, confirming the similar trend in BM SUV_mean_. The variance of the difference relative to the mean assessed by Kendall’s tau showed only one measure with a strictly significant positive correlation (tau = 0.59, *P* <0.001) in SUV_max_ of lung RMZ in the untransformed data (Table S2, SDC). The distribution plots for SUV measurements of the BM and RSM indicated possible bias (Fig. S10a-S10b, SDC).

### Interobserver Variation

**Fig. 2:**
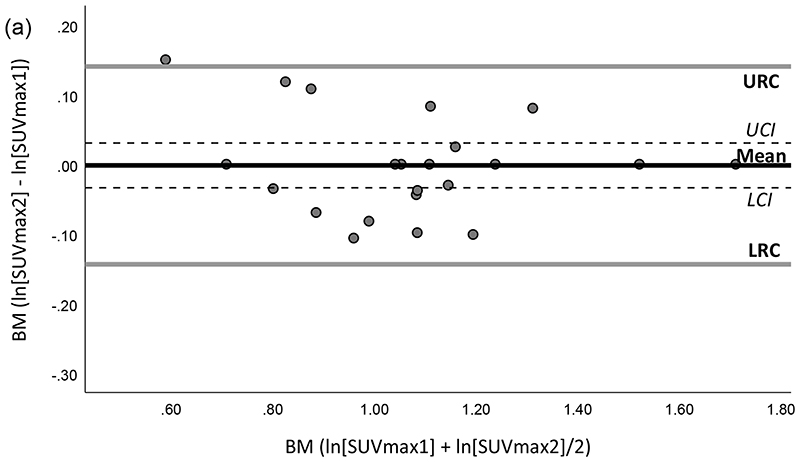

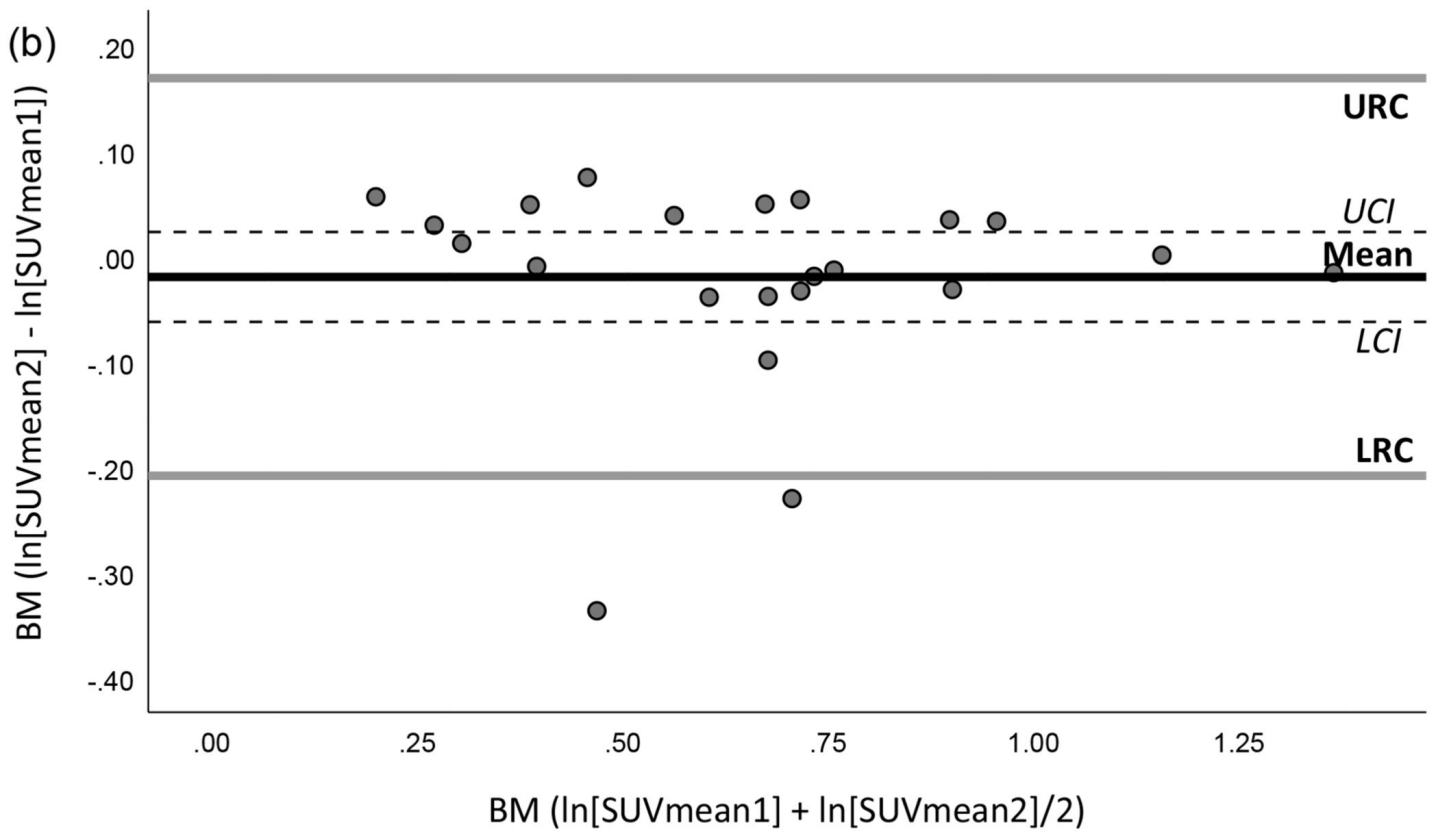

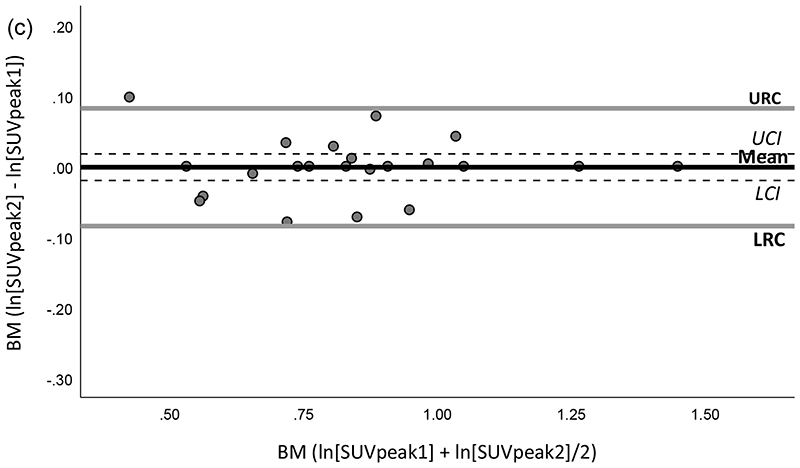

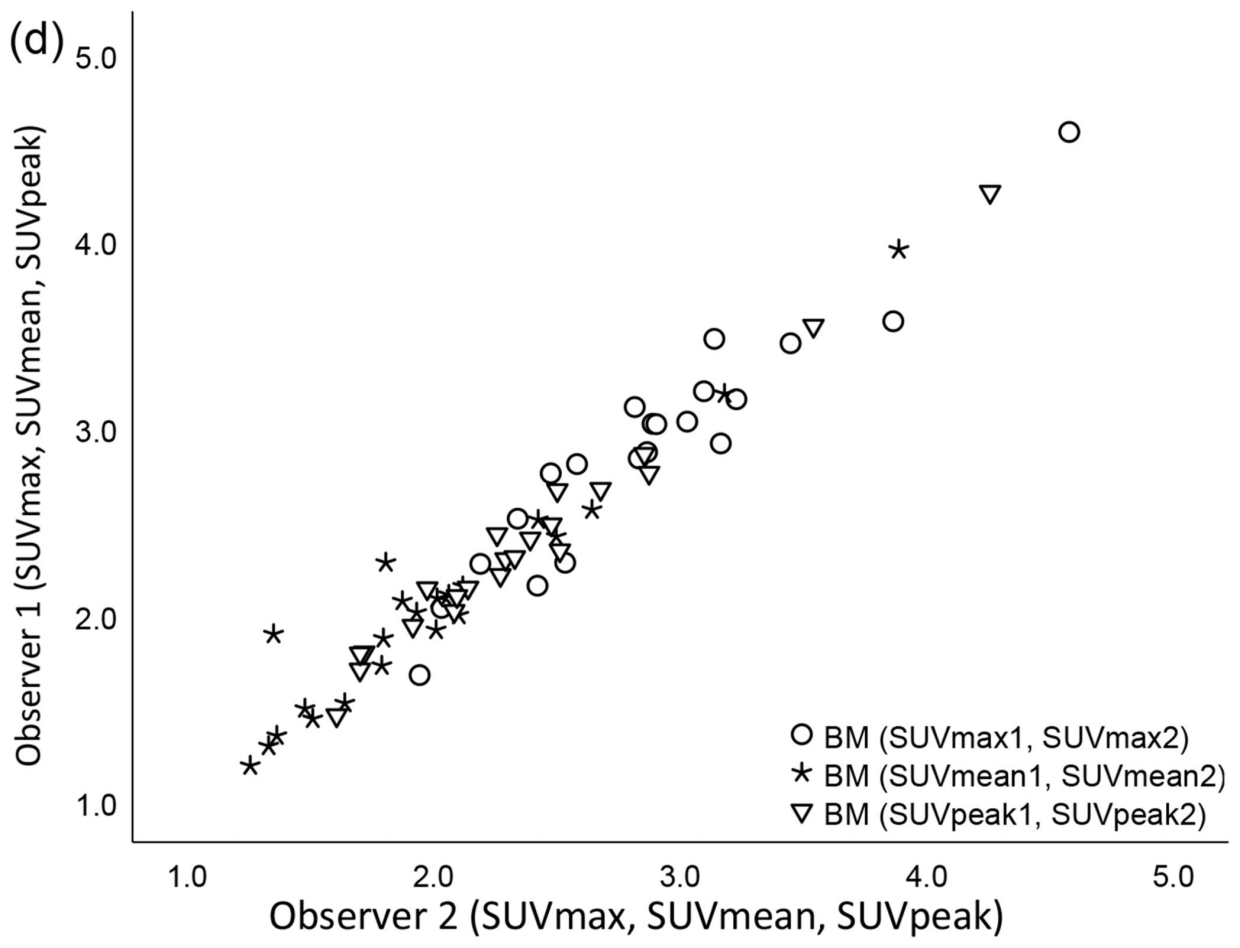

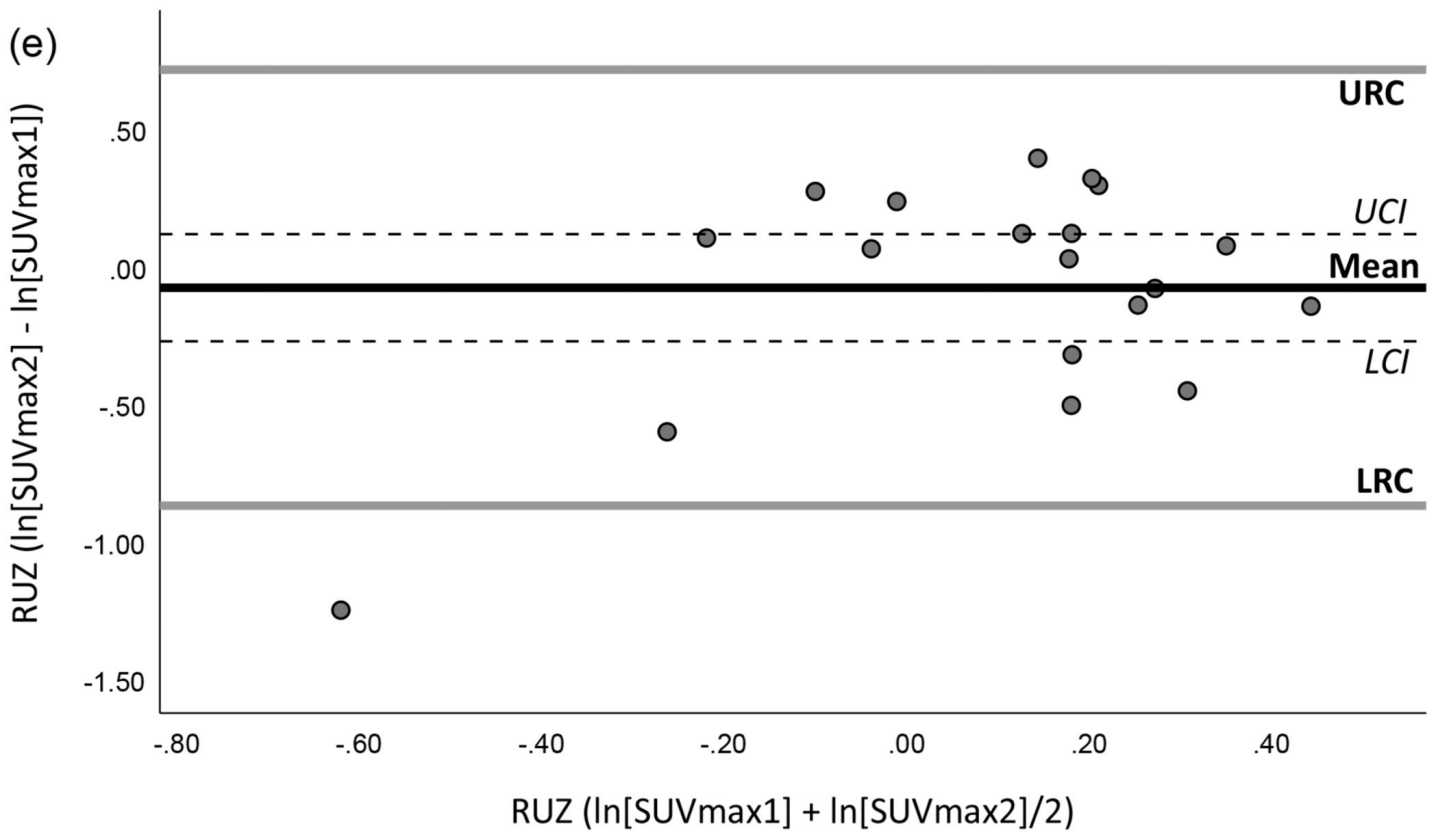

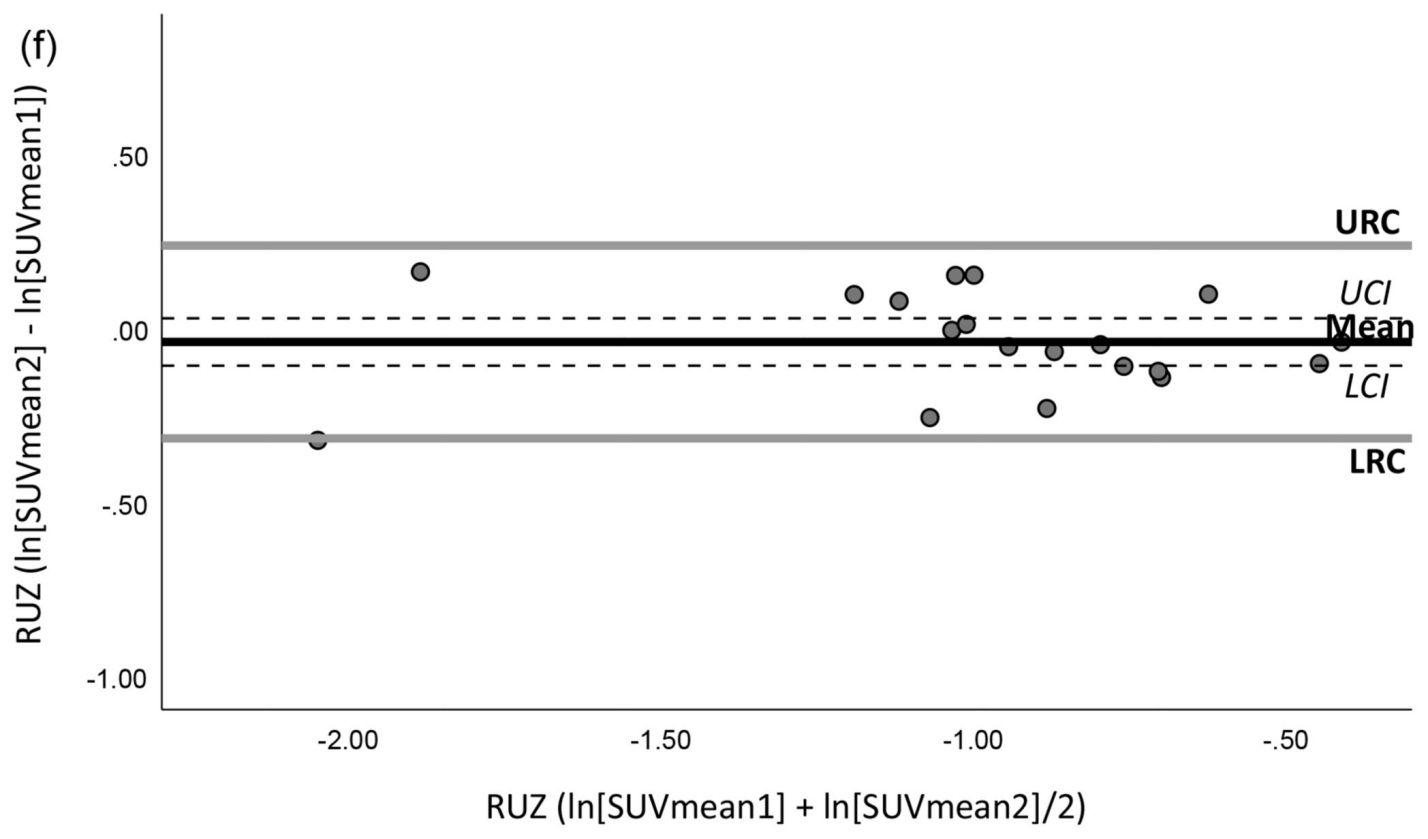

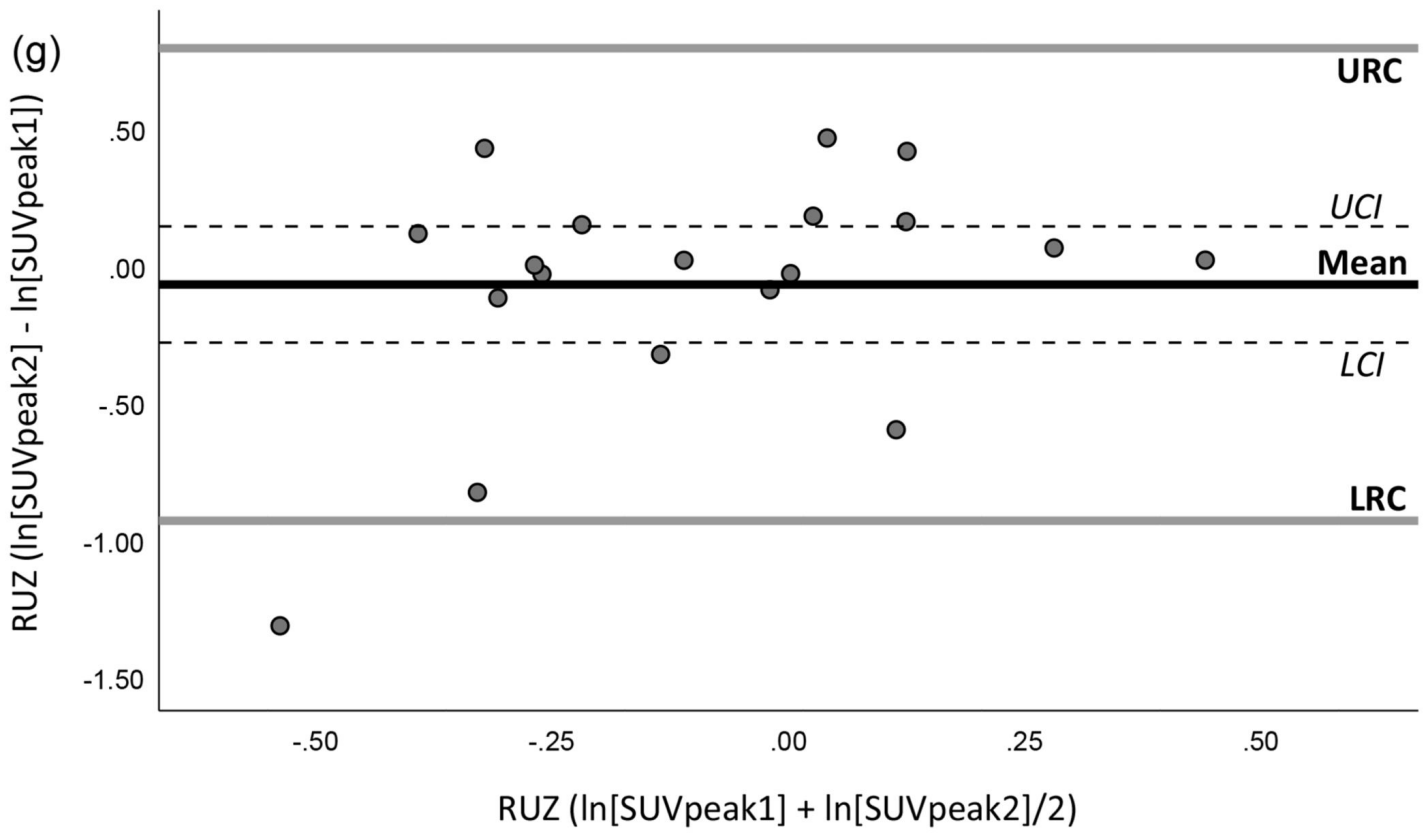

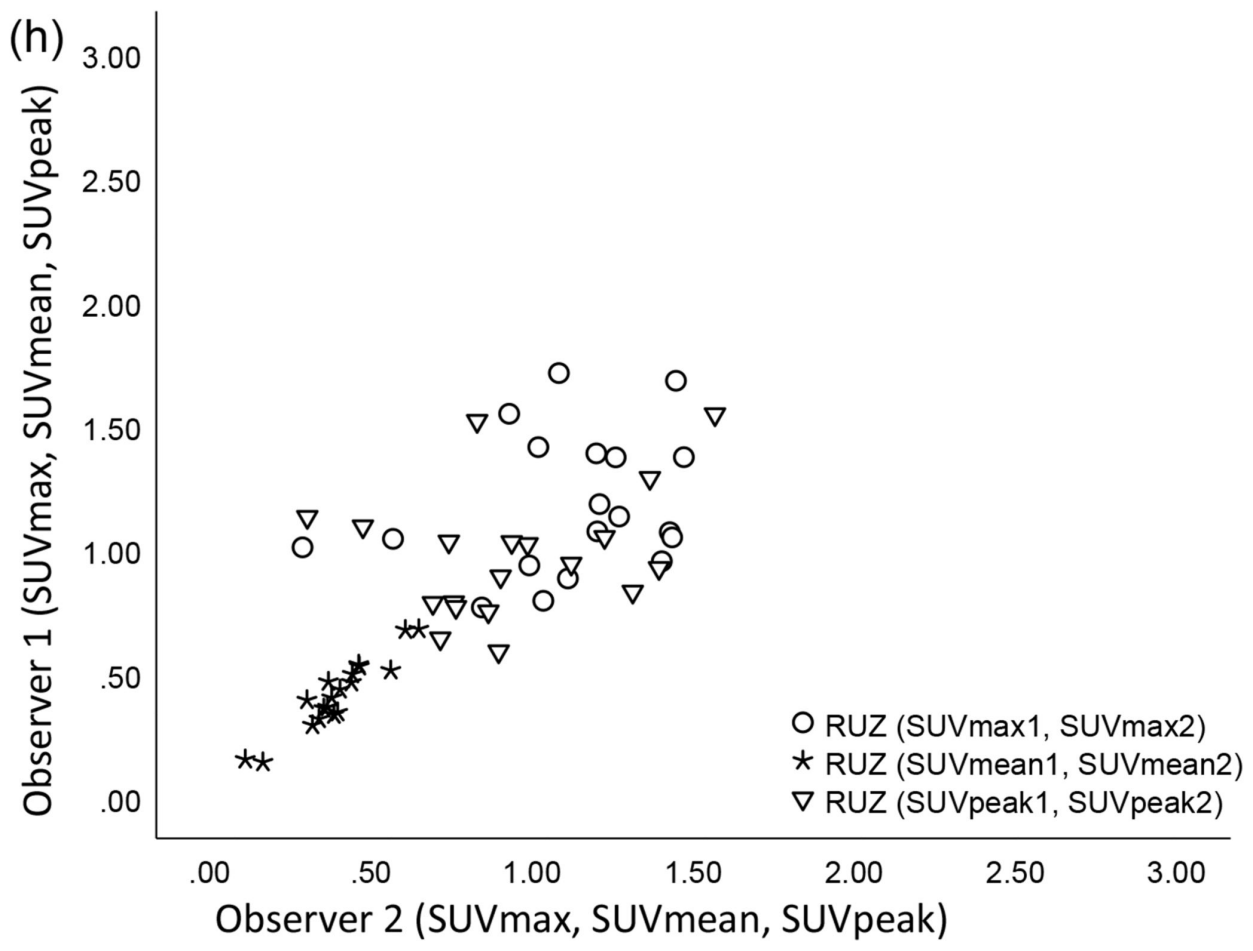
Interobserver variation of log-transformed SUV measurements including maximum, mean and peak in (a-c) bone marrow (BM) and (e-g) lung right upper zone (RUZ) illustrated by the Bland-Altman method. A simple linear regression indicated no significant bias in (a-c) BM and (e-g) lung RUZ data (*P* >0.0015). Scatter plots for distribution of interobserver measurements for different SUV parameters in (d) BM and (h) lung RUZ. Mean, mean of SUV difference between measurements of observer 1 and 2; URC/LRC, upper and lower repeatability coefficients; UCI/LCI, upper and lower 95% confidence intervals of the mean; SUV, standardized uptake value.

No significant bias *P* >0.0015 was found in the paired comparisons of the differences in interobserver measurements for all HTs. The mean of the differences for SUV parameters in all HTs between the two observers ranged from -0.28 to +0.15 (Table 4). SUV_mean_ and SUV_peak_ measurements both showed high interobserver agreement for MBP, BM and SM with wCV of ≤10.3% with narrow limits of repeatability in each tissue as shown in Table 4, for BM (Fig. 2) and for other HTs (Fig. S11-S20, SDC). The wCVs of LV were <21.6% indicating less agreement between observers with wider ranges of upper and lower RCs (Table 4). SUV_mean_ of interobserver measurements in different lung zones showed high agreement in all lung zones with wCV of ≤10.5%, but SUV_max_ and SUV_peak_ measurements showed more variations specifically in lung RUZ with wCV measured 33.1% and 36.5%, respectively as presented in Table 4 (Fig. 2).

Pearson’s Correlation for interobserver measurements was not significant (*P* >0.0015) in any SUV parameters. Kendall’s tau showed one strictly significant correlation (tau = -0.50, *P* <0.001) in SUV_max_ of lung RLZ in the log-transformed data (Table S5, SDC). The Bland-Altman plots of SUV_max_ and SUV_peak_ in MBP look fairly biased (Fig. S11a and S11c, SDC).

## Discussion

The aims of this study were to assess the test-retest repeatability and interobserver variation of HT metabolism using the SUV parameters commonly applied in ^18^F-FDG PET/CT imaging for cancer patients. We found no significant bias in the mean differences of both analyses (the test-retest) and (interobserver) measurements in different HTs.

Currently only metabolic activity of the liver and MBP are routinely used for response evaluation in patients with lymphoma [22]. Metabolic activity in the liver has been assessed by several studies [10–12,14,23]. For MBP our findings are in accord with those observed by Paquet *et al*. who found that ^18^F-FDG uptake in MBP was stable in follow-up scans of cancer-free oncology patients with wCV for SUV_max_ and SUV_mean_ of 13.1% and 12.3% [10]. Wu *et al*. and Kim *et al*. found that SUVs of MBP were stable but SUV_mean_ in Kim *et al*. study showed a significant yet small change during chemotherapy in patients with diffuse large B-cell lymphoma [24-25] confirming that MBP is stable and can be used to assess and normalise ^18^F-FDG uptake in cancer patients during treatment as applied in the Deauville scale for lymphoma [22]. However, Kramer *et al*. reported that normalisation of tumour uptake to MBP was more variable at 90 min than 60 min after ^18^F-FDG injection suggesting that MBP repeatability might be influenced by the uptake time [23]. Similar good repeatability was seen in the bone marrow in our study, with one outlier which may explain the slightly wider variations in the repeatability than other HTs as indicated by the wCVs.

Paquet *et al*. reported significant variations in the average difference of SUV_max_ and SUV_mean_ of SM which was not found in our analysis [10]. But similar to Paquet *et al*. we observed an average decrease, although not significant, in ^18^F-FDG uptake in SM from the first to second scan which was attributed by Paquet *et al*. to the reduced stress associated with repeat PET/CT examination as patients became more familiar with the procedure [10]. Although Paquet *et al*. used the trapezius muscle in their analysis and we measured SUVs on teres major, they are both in the upper back and might be influenced by similar factors such as movement or exposure to low temperature. This might be one of the reasons for the higher stability reported by Gheysens *et al*. in their repeatability analysis using segmented gluteal and quadriceps muscles with very low wCV of 2.2% and 3.6%, respectively [9].

Paquet *et al*. also noted significant variations in the average difference of SUVs in the basal region of the right lung in consecutive PET/CT scans (Table 5) [10]. One of the suggested causes is the low ^18^F-FDG uptake in the lungs leading to high susceptibility to noise when measuring SUV and the close proximity to the liver [10]. In contrast to the findings of Paquet *et al*., no significant variations were detected in the average differences in different lung zones in the current study. The better repeatability in our study might be due to the use of larger ROIs placed at least 2 cm away from the liver, a shorter period between the two scans (3.1 ± 1 vs. 271 ± 118 days) and a more rigorous repeatability study design unlike Paquet *et al*. where analysis was based on retrospective follow up of oncology patients [10].

In the repeatability study by Gheysens *et al*., a wCV of 20.7% was reported in the LV which was much lower than the wCVs we obtained for the different SUV parameters ranging from 63.60% to 69.00% [9]. The study by Gheysens *et al*. was conducted in 6 healthy individuals with a mean age ± SD of (32 ± 10 years) [9] rather than in cancer patients with a mean age of (68.6 ± 7.7 years) in our analysis. This may imply that the age and the physical condition of patients may affect the stability of ^18^F-FDG uptake in LV, but also that variability in myocardial uptake may be more common in cancer patients [15]. The findings of Thut *et al*. from 20 patients with non-Hodgkin’s lymphoma which showed high regional variability in LV with variable patterns of SUV_max_ across different regions of the LV in several PET/CT scans regardless of the fasting period also support this hypothesis [15]. Quite similar observations were reported in a retrospective study by Inglese *et al*. in 49 patients with various malignancies during treatment which showed heterogeneity of uptake in different myocardial regions and high variability in the same region at different time points [14]. On the other hand, Kim *et al*. found no significant variations in volumetric measurements of myocardial ^18^F-FDG uptake in patients with diffuse large B-cell lymphoma during treatment [25]. It may be preferable to use a more global assessment in addition to a regional evaluation when monitoring changes in ^18^F-FDG metabolism in myocardium during treatment of cancer patients. However, the simple visual assessment performed in our study indicates that the low repeatability in LV measurements is unlikely to be primarily a result of the segmentation method applied, but simply reflecting the large inter- and intraindividual variations in myocardial uptake when patients have not been instructed to follow a low-carbohydrate diet and prolonged fasting [18] and questions the use of myocardial uptake as a prognostic marker [1] unless there are carefully controlled dietary conditions.

Image interpretation of HT metabolism may be inconsistent between observers when nonstandard methods are used for measuring ^18^F-FDG uptake in HT. In our interobserver analysis using standardised placement of fixed VOIs we found low wCV indicating high agreement in MBP, BM and SM and moderate agreement in the LV for all SUV measurements. High agreement was also noted in SUV_mean_ measurements between the observers in all lung zones, but more variability in SUV_max_ and SUV_peak_ measurements. To the best of our knowledge, interobserver agreement of ^18^F-FDG uptake in HT has only been studied in the liver and brown adipose tissue [12,26-27].

Other indirect interobserver analyses have been conducted. Burger *et al*. compared two methods for measuring the background activity from different healthy tissues as reference regions for malignant lesions [28]. The study showed excellent interobserver agreement in the VOI method used for mean background activity of the respective organs including the lung, liver, skin and neck [28]. Despite the high agreement in SUV_mean_ in our interobserver measurements in the lungs, the variations in SUV_max_ and SUV_peak_ might be attributed to the observer dependent ROI sizes which may augment the effect of possible spillover from adjacent lung cancer lesions or physiological high uptake e.g. in the myocardium. Ohira *et al*. assessed interobserver variation of myocardium metabolism in 2 groups of patients on low-carbohydrate/high-fat diet and unrestricted diet respectively with cardiac sarcoidosis using pattern and regional ROI approaches [29]. Agreement in the pattern interpretation was moderate, but results showed a trend for improved agreement in the restricted diet group [29]. This may be a possible factor for the only moderate interobserver variation in the LV among patients in the current study who were not on cardiac-specific dietary restriction.

One of the interesting findings with regard to the methods of measurements is that we found large disparity in the test-retest and interobserver lung measurements of SUV_max_ and SUV_peak_ compared to SUV_mean_. Schwartz *et al*. pointed out in their phantom repeatability study that SUV_max_ and SUV_mean_ are similar when measured in smaller ROIs, more homogenous sources and at longer scan times (>3min/bed position), but the repeatability improves with larger objects and SUV_mean_ has better repeatability regardless of the ROI size [30]. Because lung tissue is more susceptible to statistical errors, using larger ROIs and SUV_mean_ for evaluating the variation in ^18^F-FDG uptake in the lung is likely to be more reliable than smaller ROIs and values derived from SUV_max_ or SUV_peak_.

With this study we also wished to formulate recommendations for future studies evaluating HT metabolism. Based on the presented repeatability analysis of HT metabolism we found both SUV_peak_ and SUV_mean_ were more stable in HT than SUV_max_. The overall test-retest repeatability and interobserver variation were better in HTs with moderate ^18^F-FDG uptake (MBP and BM) and lower in tissues with low uptake (SM and lungs). These observations in HTs agree with the pattern of repeatability observed in measurement of tumours whereby the repeatability is improved as ^18^F-FDG uptake increases [19]. Our findings also indicate that in organs with very low physiological ^18^F-FDG uptake measurements using SUV_mean_ in a larger ROI or segmented organ might be preferable. This, however, raises an issue regarding interobserver variation in defining these regions which may be improved by applying automated segmentation, e.g. based on artificial intelligence.

There are some limitations to our study. The retrospective analysis prevented the control of potential factors such as the restricted diet for LV assessment, nevertheless, analysis of other HTs under restricted diet would possibly not reflect the normal status of cancer patients having a clinical PET/CT scan. The assessment of the test-retest scans was performed by a less experienced observer, however, the subsequent interobserver analysis against an experienced reader showed good agreement and low interobserver variation. In addition, other HTs such as liver, spleen and bowel were not analysed because the original study required thoracic PET/CT scans only to assess lung cancer lesions repeatability using different breathing protocols. The test-retest and interobserver variation of liver SUV has however been previously reported [11-12,26-27]. As we only used data from lung cancer patients our results might not be applicable to patients with other types of malignancies. However, we consider the repeatability analysis and results are likely to apply across a broad range of cancers, especially as all scans were acquired prior to any treatment. Furthermore, it might be difficult to estimate correlations for the differences in the test-retest scans and interobserver measurements to the means due to random noise from the low range SUV in HT, combined with large numbers of statistical tests and small sample size. It would be desirable to validate our results in a larger independent sample, but conducting repeatability studies on large numbers of patients with repeat radiation exposure particularly for evaluation of HTs might not be feasible or ethical.

## Conclusion

HT metabolism is stable in a test-retest scenario and has high interobserver agreement. The wCV of SUV_mean_ measurements between the two scans were <20% and <10% between the observers, thus, variation in SUV_mean_ of over 20% would indicate a true change. SUV_mean_ is suggested as the most stable metric especially in organs with low ^18^F-FDG uptake (SM and lungs). For HTs with moderate uptake (MBP and BM) SUV_peak_ is suggested as the preferred metric. Test-retest measurements in LV were highly variable, irrespective of the SUV parameter used, although this might be reduced by considering automated segmentation and assessment methods that do not solely rely on regional analysis accompanied with dietary restrictions where feasible.

## Supplementary Material

Supplemental digital content

## Figures and Tables

**Table 1:** Assessment methods to evaluate variability of FDG uptake in healthy tissue

Healthy tissue	Location	Method
Mediastinal blood pool	Descending aorta	Placing of 1.5 cm diameter sphere using nudge tool on descending aorta [30 - 31] to ensure the volume of interest (VOI) is not touching the wall of aorta (Guided by CT images to avoid artefacts from adjacent structures or atherosclerotic associated inflammation).
Myocardium	Lateral wall of the left ventricle (LV)	Placing of 1.5 cm sphere using nudge tool at the highest uptake area in the lateral wall of the LV to stay within the wall boundaries in a mid-trans-axial PET/CT slice excluding artefact [30 - 31].
Bone marrow	Thoracic vertebral body at level of bifurcation of the carina	Placing of 1.5 cm diameter sphere at mid-vertebral body in a trans-axial PET/CT slice with review of sagittal CT images to confirm accurate placement and avoid areas of focal uptake, artefacts, compression fracture or severe osteoarthritic changes.
Skeletal muscle	Right and left teres major muscles	Placing of 1.5 cm diameter sphere using nudge tool in teres major at each selected skeletal muscle excluding areas of focal uptake in a trans-axial PET/CT slice.
Lungs	Both lung zones	Manual drawing of region of interest segmenting all of lung parenchyma leaving a margin to avoid overlap with pleura at a single slice in respective upper, middle and lower zones of the lungs [32], excluding the hilar vessels and any disease in a trans-axial PET/CT slice. ROI in the RLZ of the lung was placed at least 2 cm away from the liver. ROIs were used for lungs to avoid the inclusion of tumours or any possible areas of inflammation and large ROIs were drawn to reduce the possible noise effect [31] and to get better insight into SUV repeatability and variations in each zone.

**Table 2:** Characteristics of the study population

Population Characteristics	Scan 1 (test)			Scan 2 (retest)		
Patients (n) = 22	Mean ± SD	Median	Range	Mean ± SD	Median	Range
Sex (n) = F (7), M (15)						
Tumour: non-small cell lung cancer						
Age (y)	68.6 ± 7.7	70	54-85	-	-	-
Weight (kg)	76.7 ± 11.5	76.5	53-99	76.9 ± 11.9	76.5	52-99
Administered activity (MBq)	304.8 ± 45.5	304.5	213-395	307.3 ± 48.5	304	197-393
Uptake time (min)	67 ± 03	67	61-75	68 ± 04	67	61-78
Time from scan 1 to 2 (d)	-	-	-	3.1 ± 1	3.5	2-5

SD, standard deviation

**Table 3:** Characteristics of test-retest repeatability for different SUV parameters in healthy tissue

Healthy Tissue		PET parameter	Mean ± SD	*P* value ^a^	wCV(%)	Upper RC (%)	Lower RC (%)
Mediastinal blood pool		SUV_max_	0.01 ± 0.38	*0.808*	10.33	+ 31.35	- 23.87
		SUV_mean_	0.02 ± 0.25	*0.961*	10.20	+ 30.88	- 23.59
		SUV_peak_	-0.01 ± 0.27	*0.884*	9.56	+ 28.81	- 22.36
Left ventricle		SUV_max_	1.37 ± 8.04	*0.711*	69.02	+ 328.34	- 76.65
		SUV_mean_	1.33 ± 4.73	*0.528*	64.68	+ 298.53	- 74.91
		SUV_peak_	1.47 ± 5.68	*0.586*	63.63	+ 291.54	- 74.46
Bone marrow		SUV_max_	-0.13 ± 0.77	*0.833*	18.55	+ 60.27	- 37.61
		SUV_mean_	-0.004 ± 0.44	*0.408*	14.85	+ 46.80	- 31.88
		SUV_peak_	-0.08 ± 0.44	*0.615*	11.31	+ 34.59	- 25.70
Skeletal muscle	Right	SUV_max_	-0.08 ± 0.29	*0.189*	23.39	+ 79.08	- 44.16
		SUV_mean_	-0.03 ± 0.10	*0.306*	15.45	+ 48.91	- 32.85
		SUV_peak_	-0.03 ± 0.13	*0.638*	15.35	+ 48.55	- 32.68
	Left	SUV_max_	-0.10 ± 0.32	*0.445*	21.56	+ 71.81	- 41.79
		SUV_mean_	-0.02 ± 0.12	*0.685*	15.74	+ 49.94	- 33.31
		SUV_peak_	-0.02 ± 0.14	*0.638*	15.15	+ 47.83	- 32.36
Right lung	Upper	SUV_max_	-0.20 ± 0.36	*0.039*	28.24	+ 99.26	- 49.81
		SUV_mean_	0.02 ± 0.06	*0.215*	10.58	+ 32.16	- 24.33
		SUV_peak_	0.01 ± 0.27	*0.679*	27.70	+ 96.92	- 49.22
	Middle	SUV_max_	-0.20 ± 0.36	*0.808*	25.39	+ 87.23	- 46.59
		SUV_mean_	0.02 ± 0.06	*0.178*	9.85	+ 29.75	- 22.93
		SUV_peak_	0.01 ± 0.27	*0.426*	17.19	+ 55.21	- 35.57
	Lower	SUV_max_	0.01 ± 0.51	*0.783*	22.22	+ 74.39	- 42.66
		SUV_mean_	0.02 ± 0.07	*0.131*	10.53	+ 31.97	- 24.23
		SUV_peak_	0.11 ± 0.36	*0.291*	17.80	+ 57.47	- 36.50
Left lung	Upper	SUV_max_	-0.09 ± 0.54	*0.737*	31.27	+ 112.58	- 52.96
		SUV_mean_	0.01 ± 0.06	*0.433*	12.67	+ 39.20	- 28.16
		SUV_peak_	0.01 ± 0.21	*0.654*	19.48	+ 63.77	- 38.94
	Middle	SUV_max_	-0.08 ± 0.29	*0.191*	16.88	+ 54.09	- 35.10
		SUV_mean_	0.01 ± 0.07	*0.411*	15.10	+ 47.68	- 32.29
		SUV_peak_	-0.07 ± 0.24	*0.247*	18.98	+ 61.88	- 38.23
	Lower	SUV_max_	-0.01 ± 0.36	*0.986*	18.85	+ 61.38	- 38.04
		SUV_mean_	0.03 ± 0.10	*0.192*	18.48	+ 60.01	- 37.51
		SUV_peak_	0.09 ± 0.26	*0.122*	15.40	+ 48.73	- 32.77

SUV, standardized uptake value; Mean ± SD of intrasubject difference between scan 1 and scan 2

a *P* value of the difference from Wilcoxon signed rank test

wCV, within-subject coefficient of variation; RC, repeatability coefficients.

**Table 4:** Characteristics of interobserver variation for different SUV parameters in healthy tissue

Healthy tissue		PET parameters	Mean ± SD	*P* value ^a^	wCV(%)	Upper RC (%)	Lower RC (%)
Mediastinal blood pool		SUV_max_	0.08 ± 0.45	*0.961*	10.85	+ 33.06	- 24.85
		SUV_mean_	0.01 ± 0.18	*0.884*	6.10	+ 17.84	- 15.14
		SUV_peak_	0.01 ± 0.18	*0.808*	5.41	+ 15.72	- 13.58
Left ventricle		SUV_max_	0.15 ± 2.00	*0.397*	20.29	+ 66.89	- 40.08
		SUV_mean_	-0.28 ± 1.18	*0.943*	21.61	+ 72.01	- 41.87
		SUV_peak_	0.13 ± 1.57	*0.363*	16.78	+ 53.71	- 34.94
Bone marrow		SUV_max_	-0.01 ± 0.19	*0.654*	5.26	+ 15.26	- 13.24
		SUV_mean_	-0.04 ± 0.17	*0.884*	7.05	+ 20.77	- 17.20
		SUV_peak_	0 ± 0.09	*0.852*	3.06	+ 8.70	- 8.00
Skeletal muscle	Right	SUV_max_	0.09 ± 0.24	*0.123*	17.26	+ 55.47	- 35.68
		SUV_mean_	-0.02 ± 0.09	*0.131*	9.94	+ 30.04	- 23.10
		SUV_peak_	0 ± 0.11	*0.783*	10.28	+ 31.17	- 23.76
	Left	SUV_max_	-0.01 ± 0.18	*0.884*	12.66	+ 39.16	-28.14
		SUV_mean_	-0.04 ± 0.08	*0.020*	8.12	+ 24.16	- 19.46
		SUV_peak_	-0.03 ± 0.07	*0.101*	6.85	+ 20.16	- 16.78
Right lung	Upper	SUV_max_	-0.06 ± 0.36	*0.629*	33.10	+ 120.88	- 54.72
		SUV_mean_	-0.02 ± 0.05	*0.126*	10.52	+ 31.95	- 24.22
		SUV_peak_	-0.04 ± 0.35	*0.968*	36.47	+ 136.75	- 57.76
	Middle	SUV_max_	-0.12 ± 0.49	*0.291*	25.05	+ 85.83	- 46.19
		SUV_mean_	0 ± 0.05	*0.485*	7.60	+ 22.48	- 18.35
		SUV_peak_	-0.11 ± 0.34	*0.178*	24.47	+ 83.44	-45.49
	Lower	SUV_max_	0.03 ± 0.26	*0.543*	15.67	+ 49.71	-33.20
		SUV_mean_	0 ± 0.04	*0.758*	6.12	+ 17.90	- 15.18
		SUV_peak_	0.02 ± 0.29	*0.638*	21.90	+ 73.14	- 42.24
Left lung	Upper	SUV_max_	-0.03 ± 0.35	*0.478*	22.28	+ 74.63	- 42.74
		SUV_mean_	-0.01 ± 0.05	*0.455*	9.42	+ 28.35	- 22.09
		SUV_peak_	-0.06 ± 0.22	*0.391*	18.72	+ 60.89	- 37.85
	Middle	SUV_max_	-0.09 ± 0.36	*0.263*	20.85	+ 69.04	- 40.84
		SUV_mean_	0 ± 0.03	*0.502*	4.78	+ 13.81	- 12.14
		SUV_peak_	-0.13 ± 0.32	*0.086*	20.17	+ 66.40	- 39.90
	Lower	SUV_max_	-0.12 ± 0.32	*0.159*	15.84	+ 50.31	- 33.47
		SUV_mean_	-0.01 ± 0.03	*0.131*	4.77	+ 13.78	- 12.11
		SUV_peak_	-0.13 ± 0.38	*0.192*	19.78	+ 64.92	- 39.37

SUV, standardized uptake value; Mean ± SD of intrasubject difference between observer 1 and observer 2

a *P* value of the difference from Wilcoxon signed rank test

wCV, within-subject coefficient of variation; RC, repeatability coefficients.

**Table 5:** Repeatability studies on healthy tissue from literature

Publication	Year	Healthy tissue	Tissue ROI	PET parameter	Repeatability method	Repeatability measurements	Variability
Gheysens *et al*.[9]	2015	Myocardium	3 VOIs in left ventricle	SUV*	ICC and CV%	(0.93, 20.7%)^‡^	High
		Skeletal muscle	Segmented ROI of gluteal muscle	SUV*	ICC and CV%	(0.88, 2.2%)^‡^	Low
			Segmented ROI of quadriceps muscle			(0.96, 3.6%)^‡^	Low
Paquet *et al*.[10]	2004	Mediastinum	ROI on upper region, level of large vessels	SUV_max_	Paired *t*-test, ICC and CV%	0.06 ± 0.39 (0.67, 13.1%)^‡^	*P* = NS
				SUV_mean_		0.02 ± 0.28 (0.65, 12.3%)^‡^	*P* = NS
		Skeletal muscle	ROI on trapezius muscle	SUV_max_	Paired *t*-test	0.07 ± 0.28	*P* < 0.05
				SUV_mean_		0.05 ± 0.16	*P* < 0.05
		Lungs	ROI on lower region of right lung at distance to diaphragm	SUV_max_	Paired *t*-test	0.06 ± 0.24	*P* < 0.05
				SUV_mean_		0.03 ± 0.12	*P* < 0.05

NS, not significant

* SUV parameter was not identified

ICC, intraclass correlation coefficients; CV, coefficient of variation

‡ (ICC, CV%); Measurements mean ± SD.
