# Supplementary material for: Test-retest Repeatability and Interobserver Variation of Healthy Tissue Metabolism using FDG-PET/CT of the Thorax among Lung Cancer Patients
Source: Nucl Med Commun. Author manuscript; Available in PMC 2022 May 1. (PMC7612596; doi:10.1097/MNM.0000000000001537)
Supplement: Supplemental digital content [file EMS140744-supplement-Supplemental_digital_content.pdf]

Supplemental Digital Content (SDC)

Test-retest Repeatability and Interobserver Variation of Healthy Tissue Metabolism using FDG-PET/CT of the Thorax among Lung Cancer Patients

Journal: Nuclear Medicine Communications

Afnan Malaih[1], Joel T. Dunn[1], Lotte Nygård[2], David G. Kovacs[3], Flemming L. Andersen[3], Sally F. Barrington[1], Barbara M. Fischer[1,3]

[1]School of Biomedical Engineering and Imaging Sciences, PET Imaging Centre, St Thomas' Hospital, King's College London, London, UK

[2]Department of Oncology, Rigshospitalet, Copenhagen University Hospital, Copenhagen, Denmark

[3]Department of Clinical Physiology, Nuclear Medicine and PET, Rigshospitalet, University of Copenhagen, Copenhagen, Denmark

Corresponding Author:

Afnan Malaih, MSc., PhD Candidate

School of Biomedical Engineering and Imaging Sciences, King's College London

St Thomas' Hospital, Westminster Bridge Road

London SE1 7EH

E-mail: afnan.malaih@kcl.ac.uk

Table S1 Mean and SD of scan 1 and scan 2 with their absolute, original and log-transformed differences

Healthy tissue		PET parameters	Scan 1	Scan 2	|*d*|	*d*	*p* value [a]	*p* value [b]	*p* value [c]	d_{ln}	*p* value [d]	*p* value [e]	*p* value [f]
**Mediastinal blood pool**		SUV_{max}	2.81 ± 0.50	2.81 ± 0.60	0.29 ± 0.24	0.01 ± 0.38	*0.567*	*0.906*	*0.808*	-0.01 ± 0.14	*0.957*	*0.865*	*0.733*
		SUV_{mean}	1.95 ± 0.31	1.97 ± 0.36	0.19 ± 0.16	0.02 ± 0.25	*0.310*	*0.745*	*0.961*	0.003± 0.14	*0.861*	*0.745*	*0.910*
		SUV_{peak}	2.26 ± 0.35	2.25 ± 0.39	0.21 ± 0.16	-0.01 ± 0.27	*0.827*	*0.933*	*0.884*	-0.01 ± 0.13	*0.685*	*0.933*	*0.935*
**Left ventricle**		SUV_{max}	8.67 ± 6.48	10.04 ± 7.02	5.96 ± 5.39	1.37 ± 8.04	*0.449*	*0.479*	*0.711*	0.14 ± 0.74	*0.135*	*0.431*	*0.500*
		SUV_{mean}	5.08 ± 3.88	6.41 ± 4.60	3.36 ± 3.51	1.33 ± 4.73	*0.089*	*0.249*	*0.528*	0.21 ± 0.71	*0.348*	*0.219*	*0.327*
		SUV_{peak}	6.20 ± 4.68	7.66 ± 5.30	4.1 ± 4.1	1.47 ± 5.68	*0.184*	*0.289*	*0.586*	-0.14 ± 0.70	*0.192*	*0.229*	*0.396*
**Bone marrow**		SUV_{max}	3.08 ± 0.87	2.95 ± 0.61	0.46 ± 0.62	-0.13 ± 0.77	*<0.001****	*0.446*	*0.833*	-0.03 ± 0.24	*0.002***	*0.594*	*0.808*
		SUV_{mean}	1.99 ± 0.71	1.99 ± 0.44	0.27 ± 0.35	-0.004 ± 0.44	*<0.001****	*0.970*	*0.408*	0.03 ± 0.20	*0.096*	*0.447*	*0.236*
		SUV_{peak}	2.37 ± 0.70	2.30 ± 0.46	0.25 ± 0.37	-0.08 ± 0.44	*<0.001****	*0.427*	*0.615*	-0.02 ± 0.15	*<0.001****	*0.628*	*0.527*
**Skeletal muscle**	Right	SUV_{max}	1.16 ± 0.26	1.08 ± 0.26	0.24 ± 0.17	-0.08 ± 0.29	*0.866*	*0.199*	*0.189*	-0.09 ± 0.30	*0.027**	*0.194*	*0.123*
		SUV_{mean}	0.65 ± 0.12	0.62 ± 0.16	0.07 ± 0.07	-0.03 ± 0.10	*0.003***	*0.228*	*0.306*	-0.06 ± 0.20	*<0.001****	*0.195*	*0.291*
		SUV_{peak}	0.80 ± 0.17	0.77 ± 0.18	0.09 ± 0.09	-0.03 ± 0.13	*0.044**	*0.352*	*0.638*	-0.04 ± 0.21	*0.001***	*0.355*	*0.661*
	Left	SUV_{max}	1.16 ± 0.35	1.06 ± 0.25	0.24 ± 0.24	-0.10 ± 0.32	*0.025**	*0.180*	*0.445*	-0.07 ± 0.28	*0.207*	*0.225*	*0.465*
		SUV_{mean}	0.65 ± 0.14	0.63 ± 0.15	0.09 ± 0.08	-0.02 ± 0.12	*0.103*	*0.685*	*0.685*	-0.04 ± 0.21	*0.012**	*0.321*	*0.570*
		SUV_{peak}	0.79 ± 0.15	0.77 ± 0.16	0.10 ± 0.09	-0.02 ± 0.14	*0.401*	*0.402*	*0.638*	-0.04 ± 0.20	*0.133*	*0.381*	*0.733*
**Right lung**	Upper	SUV_{max}	1.27 ± 0.42	1.07 ± 0.29	0.28 ± 0.29	-0.20 ± 0.36	*0.092*	*0.032**	*0.039**	-0.14 ± 0.35	*0.194*	*0.120*	*0.043*
		SUV_{mean}	0.38 ± 0.12	0.40 ± 0.13	0.04 ± 0.05	0.02 ± 0.06	*0.204*	*0.187*	*0.215*	0.06 ± 0.14	*0.283*	*0.107*	*0.184*
		SUV_{peak}	0.91 ± 0.28	0.92 ± 0.31	0.20 ± 0.18	0.01 ± 0.27	*0.477*	*0.940*	*0.679*	0.004 ± 0.35	*0.190*	*0.961*	*0.647*
	Middle	SUV_{max}	1.34 ± 0.46	1.34 ± 0.47	0.37 ± 0.34	0.002 ± 0.51	*0.706*	*0.989*	*0.808*	-0.01 ± 0.32	*0.931*	*0.944*	*0.935*
		SUV_{mean}	0.45 ± 0.13	0.47 ± 0.15	0.04 ± 0.04	0.02 ± 0.05	*0.159*	*0.106*	*0.178*	0.02 ± 0.13	*0.216*	*0.405*	*0.236*
		SUV_{peak}	1.14 ± 0.38	1.17 ± 0.37	0.23 ± 0.19	0.03 ± 0.30	*0.443*	*0.629*	*0.426*	0.03 ± 0.22	*0.364*	*0.548*	*0.485*
	Lower	SUV_{max}	1.62 ± 0.40	1.63 ± 0.53	0.32 ± 0.39	0.01 ± 0.51	*0.005***	*0.922*	*0.783*	-0.01 ± 0.28	*0.055*	*0.940*	*0.570*
		SUV_{mean}	0.50 ± 0.14	0.52 ± 0.16	0.06 ± 0.04	0.02 ± 0.07	*0.940*	*0.146*	*0.131*	0.03 ± 0.14	*0.892*	*0.306*	*0.249*
		SUV_{peak}	1.25 ± 0.36	1.36 ± 0.46	0.24 ± 0.29	0.11 ± 0.36	*0.002***	*0.149*	*0.291*	0.08 ± 0.23	*0.041*	*0.120*	*0.306*
**Left lung**	Upper	SUV_{max}	1.19 ± 0.46	1.10 ± 0.32	0.33 ± 0.43	-0.09 ± 0.54	*0.122*	*0.468*	*0.737*	-0.06 ± 0.38	*0.122*	*0.480*	*0.737*
		SUV_{mean}	0.39 ± 0.08	0.40 ± 0.10	0.05 ± 0.04	0.01 ± 0.06	*0.980*	*0.445*	*0.433*	0.02 ± 0.17	*0.671*	*0.628*	*0.526*
		SUV_{peak}	0.90 ± 0.15	0.91 ± 0.20	0.17 ± 0.11	0.01 ± 0.21	*0.380*	*0.753*	*0.654*	0.002 ± 0.25	*0.548*	*0.969*	*0.794*
	Middle	SUV_{max}	1.32 ± 0.34	1.23 ± 0.36	0.26 ± 0.15	-0.08 ± 0.29	*0.334*	*0.218*	*0.191*	-0.07 ± 0.22	*0.196*	*0.148*	*0.117*
		SUV_{mean}	0.43 ± 0.12	0.45 ± 0.13	0.06 ± 0.04	0.01 ± 0.07	*0.953*	*0.374*	*0.411*	0.02 ± 0.20	*0.390*	*0.671*	*0.455*
		SUV_{peak}	1.12 ± 0.27	1.04 ± 0.29	0.21 ± 0.13	-0.07 ± 0.24	*0.742*	*0.225*	*0.247*	-0.07 ± 0.25	*0.450*	*0.205*	*0.263*
	Lower	SUV_{max}	1.52 ± 0.34	1.51 ± 0.41	0.27 ± 0.23	-0.01 ± 0.36	*0.790*	*0.882*	*0.986*	-0.02 ± 0.24	*0.968*	*0.735*	*0.848*
		SUV_{mean}	0.55 ± 0.18	0.58 ± 0.19	0.08 ± 0.07	0.03 ± 0.1	*0.313*	*0.152*	*0.192*	0.06 ± 0.24	*0.506*	*0.305*	*0.205*
		SUV_{peak}	1.12 ± 0.32	1.21 ± 0.36	0.19 ± 0.19	0.09 ± 0.26	*0.009***	*0.109*	*0.122*	0.07 ± 0.20	*0.405*	*0.107*	*0.099*

SUV standardized uptake value, |*d*| absolute difference of |retest – test|, *d* difference of original values (scan2 – scan1) with the signs (+/-), [a]*p*-value of *d* from Shapiro-Wilk test, [b]*p*-value of *d* from paired sample t-test, [c]*p*-value of *d* from Wilcoxon signed rank test, d_{ln} log-transformed difference [ln(scan2) – ln(scan1)], [d]*p*-value of d_{ln} from Shapiro-Wilk test, [e]*p*-value of d_{ln} from paired sample t-test, [f]*p*-value of d_{ln} from Wilcoxon signed rank test, ****p*** ≤0.05, *****p*** ≤0.01 and ******p*** ≤0.001

Table S2 Correlations between the difference of (scan 1 and scan 2) and the means of SUVs (maximum, mean and peak)

Healthy tissue		PET parameter	Kendall τ				Pearson	
			Absolute difference	Absolute Log-difference	Original difference	Log-difference	Original difference	Log-difference
Mediastinal blood pool		SUV_{max}	(-0.074, *p*= 0.632)	(-0.195, *p*= 0.204)	(0.169, *p*= 0.271)	(0.186, *p*= 0.225)	(0.274, *p*= 0.218)	(0.392, *p*= 0.071)
		SUV_{mean}	(0.013, *p*= 0.933)	(-0.091, *p*= 0.554)	(0.013, *p*= 0.993)	(0.013, *p*= 0.933)	(0.225, *p*= 0.314)	(0.323, *p*= 0.142)
		SUV_{peak}	(-0.030, *p*= 0.844)	(-0.126, *p*= 0.414)	(0.065, *p*= 0.672)	(0.048, *p*= *0.756*)	(0.164, *p*= 0.465)	(0.402, *p*= 0.064)
Left ventricle		SUV_{max}	(0.542, *p*= 0.002**)	(0.242, *p*= 0.161)	(0.007, *p*= 0.970)	(0.124, *p*= 0.472)	(0.083, *p*= 0.742)	(0.095, *p*= 0.708)
		SUV_{mean}	(0.438, *p*= 0.011*)	(0.098, *p*= 0.570)	(0.033, *p*= 0.850)	(0.059, *p*= 0.733)	(0.183, *p*= 0.466)	(0.148, *p*= 0.559)
		SUV_{peak}	(0.477, *p*= 0.006**)	(0.229, *p*= 0.185)	(0.033, *p*= 0.850)	(0.033, *p*= 0.850)	(0.134, *p*= 0.596)	(0.114, *p*= 0.653)
Bone marrow		SUV_{max}	(-0.039, *p*= 0.800)	(-0.203, *p*= 0.185)	(-0.273, *p*= 0.076)	(-0.203, *p*= 0.185)	(-0.385, *p*= 0.077)	(-0.286, *p*= 0.198)
		SUV_{mean}	(-0.108, *p*= 0.481)	(-0.290, *p*= 0.059)	(-0.437, *p*= 0.004**)	(-0.472, *p*= 0.002**)	(-0.636, *p*= 0.001**)	(-0.662, *p*= 0.001**)
		SUV_{peak}	(0.299, *p*= 0.052)	(0.212, *p*= 0.167)	(-0.186, *p*= 0.225)	(-0.229, *p*= 0.135)	(-0.572, *p*= 0.005**)	(-0.453, *p*= 0.034*)
Skeletal muscle	Right	SUV_{max}	(0.273, *p*= 0.076)	(0.048, *p*= 0.756)	(0.048, *p*= 0.756)	(0.048, *p*= 0.756)	(0.018, *p*= 0.936)	(0.286, *p*= 0.197)
		SUV_{mean}	(-0.030, *p*= 0.884)	(-0.150, *p*= 0.324)	(0.169, *p*= 0.271)	(0.160, *p*= 0.297)	(0.415, *p*= 0.055)	(0.478, *p*= 0.024*)
		SUV_{peak}	(-0.030, *p*= 0.844)	(0.022, *p*= 0.888)	(0.004, *p*= 0.978)	(0.022, *p*= 0.888)	(0.082, *p*= 0.715)	(0.275, *p*= 0.216)
	Left	SUV_{max}	(0.065, *p*= 0.672)	(-0.065, *p*= 0.672)	(-0.134, *p*= 0.382)	(-0.100, *p*= 0.517)	(-0.365, *p*= 0.094)	(-0.176, *p*= 0.432)
		SUV_{mean}	(-0.048, *p*= 0.756)	(-0.169, *p*= 0.271)	(0.195, *p*= 0.204)	(0.152, *p*= 0.324)	(0.100, *p*= 0.657)	(0.214, *p*= 0.338)
		SUV_{peak}	(-0.048, *p*= 0.756)	(-0.186, *p*= 0.225)	(0.126, *p*= 0.414)	(0.126, *p*= 0.414)	(0.096, *p*= 0.672)	(0.228, *p*= 0.307)
Right lung	Upper	SUV_{max}	(0.046, *p*= 0.791)	(-0.098, *p*= 0.570)	(-0.268, *p*= 0.120)	(-0.137, *p*= 0.426)	(-0.412, *p*= 0.089)	(-0.449, *p*= 0.062)
		SUV_{mean}	(0.320, *p*= 0.063)	(0.176, *p*= 0.306)	(0.072, *p*= 0.677)	(0.033, *p*= 0.850)	(0.029, *p*= 0.910)	(-0.191, *p*= 0.447)
		SUV_{peak}	(0.176, *p*= 0.306)	(0.033, *p*= 0.350)	(0.007, *p*= 0.970)	(0.020, *p*= 0.910)	(0.140, *p*= 0.579)	(-0.056, *p*= 0.826)
	Middle	SUV_{max}	(0.593, *p*<0.001***)	(0.429, *p*= 0.005**)	(0.048, *p*= 0.756)	(0.091, *p*= 0.554)	(0.044, *p*= 0.847)	(0.091, *p*= 0.689)
		SUV_{mean}	(0.022, *p*= 0.888)	(-0.238, *p*= 0.121)	(0.177, *p*= 0.248)	(0.117, *p*= 0.446)	(0.416, *p*= 0.054)	(0.572, *p*= 0.005**)
		SUV_{peak}	(0.351, *p*= 0.022*)	(0.160, *p*= 0.297)	(-0.022, *p*= 0.888)	(-0.039, *p*= 0.800)	(-0.012, *p*= 0.959)	(-0.012, *p*= 0.957)
	Lower	SUV_{max}	(0.100, *p*= 0.517)	(-0.108, *p*= 0.481)	(0.108, *p*= 0.481)	(0.065, *p*= 0.672)	(0.291, *p*= 0.189)	(0.104, *p*= 0.645)
		SUV_{mean}	(0.203, *p*= 0.185)	(-0.013, *p*= 0.933)	(0.299, *p*= 0.052)	(0.247, *p*= 0.108)	(0.228, *p*= 0.308)	(0.364, *p*= 0.096)
		SUV_{peak}	(0.359, *p*= 0.019*)	(0.247, *p*= 0.108)	(0.048, *p*= 0.756)	(0.065, *p*= 0.672)	(0.308, *p*= 0.163)	(0.068, *p*= 0.763)
Left lung	Upper	SUV_{max}	(0.263, *p*= 0.105)	(0.189, *p*= 0.243)	(-0.032, *p*= 0.846)	(-0.011, *p*= 0.948)	(-0.352, *p*= 0.128)	(-0.118, *p*= 0.621)
		SUV_{mean}	(0.032, *p*= 0.846)	(-0.137, *p*= 0.399)	(0.158, *p*= 0.330)	(0.158, *p*= 0.330)	(0.218, *p*= 0.355)	(0.344, *p*= 0.137)
		SUV_{peak}	(-0.116, *p*= 0.475)	(-0.295, *p*= 0.069)	(0.189, *p*= 0.243)	(0.147, *p*= 0.364)	(0.310, *p*= 0.183)	(0.420, *p*= 0.065)
	Middle	SUV_{max}	(0.358, *p*= 0.027*)	(0.147, *p*= 0.364)	(0.053, *p*= 0.746)	(0.095, *p*= 0.559)	(0.089, *p*= 0.709)	(0.183, *p*= 0.439)
		SUV_{mean}	(0.011, *p*= 0.948)	(-0.147, *p*= 0.364)	(0.263, *p*= 0.105)	(0.242, *p*= 0.136)	(0.375, *p*= 0.104)	(0.294, *p*= 0.208)
		SUV_{peak}	(-0.032, *p*= 0.846)	(-0.158, *p*= 0.330)	(-0.021, *p*= 0.897)	(0.053, *p*= 0.746)	(0.114, *p*= 0.631)	(0.162, *p*= 0.494)
	Lower	SUV_{max}	(-0.038, *p*= 0.809)	(-0.152, *p*= 0.334)	(0.152, *p*= 0.334)	(0.152, *p*= 0.334)	(0.197, *p*= 0.393)	(0.206, *p*= 0.371)
		SUV_{mean}	(0.01, *p*= 0.952)	(-0.219, *p*= 0.165)	(0.038, *p*= 0.809)	(0.000, *p*= 1.00)	(0.073, *p*= 0.752)	(0.134, *p*= 0.561)
		SUV_{peak}	(0.295, *p*= 0.061)	(0.057, *p*= 0.717)	(0.162, *p*= 0.305)	(0.105, *p*= 0.506)	(0.164, *p*= 0.479)	(0.138, *p*= 0.550)

SUV standardized uptake value, values in parentheses indicate (correlation coefficient, significance), *$p \leq 0.05$, ** $p \leq 0.01$ and *** $p \leq 0.001$

Table S3 Within-subject coefficient of variation (wCV) and repeatability Coefficients (RC) with 95% CIs for Test-Retest Assessment of HT

Healthy Tissue		PET parameter	wCV(%)	RC_{ln}	Upper RC (%)	Lower RC (%)	95% CI for URC	95% CI for LRC
**Mediastinal blood pool**		SUV_{max}	10.33	± 0.273	+ 31.35	- 23.87	23.4 to 47.7	-32.3 to -18.9
		SUV_{mean}	10.20	± 0.269	+ 30.88	- 23.59	22.9 to 46.9	-31.9 to -18.7
		SUV_{peak}	9.56	± 0.253	+ 28.81	- 22.36	21.5 to 43.6	-30.3 to -17.7
**Left ventricle**		SUV_{max}	69.02	± 1.455	+ 328.34	- 76.65	206.3 to 699.9	-87.5 to -67.4
		SUV_{mean}	64.68	± 1.383	+ 298.53	- 74.91	189.8 to 621.7	-86.1 to -65.5
		SUV_{peak}	63.63	± 1.365	+ 291.54	- 74.46	185.8 to 603.3	-85.8 to -65
**Bone marrow**		SUV_{max}	18.55	± 0.472	+ 60.27	- 37.61	43.8 to 96.3	-49.1 to -42.4
		SUV_{mean}	14.85	± 0.384	+ 46.80	- 31.88	34.4 to 73.1	-42.2 to -25.6
		SUV_{peak}	11.31/7.47*	± 0.297/0.200*	+ 34.59/22.10*	- 25.70/-18.10*	25.7 to 52.9/16.6 to 33.1*	-34.6 to -20.4/-24.9 to -14.3*
**Skeletal muscle**	Right	SUV_{max}	23.39	± 0.583	+ 79.08	- 44.16	56.6 to 130.1	-56.5 to -36.1
		SUV_{mean}	15.45(6.99)	± 0.398(0.193)	+ 48.91(21.36)	- 32.85(-17.61)	35.8 to 76.6 (14.8 to 27.6)	-43.4 to -26.4(-27.6 to -14.8)
		SUV_{peak}	15.35(8.99)	± 0.396(0.249)	+ 48.55(28.44)	- 32.68(-22.14)	35.6 to 76.1(19.2 to 35.6)	-43.2 to -26.3(-35.6 to -19.2)
	Left	SUV_{max}	21.56	± 0.541	+ 71.81	- 41.79	51.6 to 116.7	-53.8 to -34.0
		SUV_{mean}	15.74	± 0.405	+ 49.94	- 33.31	36.6 to 78.4	-43.9 to -26.8
		SUV_{peak}	15.15	± 0.391	+ 47.83	- 32.36	35.1 to 74.8	-42.8 to -25.8
**Right lung**	Upper	SUV_{max}	28.24	± 0.689	+ 99.26	- 49.81	69.9 to 167.7	-62.6 to -41.1
		SUV_{mean}	10.58	± 0.279	+ 32.16	- 24.33	23.9 to 50.7	-32.9 to -19.3
		SUV_{peak}	27.70	± 0.678	+ 96.92	- 49.22	68.5 to 163.5	-62.1 to -40.6
	Middle	SUV_{max}	25.39	± 0.627	+ 87.23	- 46.59	61.9 to 144.9	-59.2 to -38.3
		SUV_{mean}	9.85	± 0.260	+ 29.75	- 22.93	22.1 to 44.9	-31.0 to -18.1
		SUV_{peak}	17.19	± 0.440	+ 55.21	- 35.57	40.3 to 87.5	-46.7 to -28.7
	Lower	SUV_{max}	22.22	± 0.556	+ 74.39	- 42.66	53.4 to 121.3	-54.8 to -34.8
		SUV_{mean}	10.53	± 0.277	+ 31.97	- 24.23	23.8 to 48.6	-32.7 to -19.2
		SUV_{peak}	17.80	± 0.454	+ 57.47	- 36.50	41.8 to 91.3	-47.7 to -29.5
**Left lung**	Upper	SUV_{max}	31.27	± 0.754	+ 112.58	- 52.96	78.6 to 193.7	-65.9 to -44.0
		SUV_{mean}	12.67	± 0.331	+ 39.20	- 28.16	29.0 to 60.5	-37.7 to -22.5
		SUV_{peak}	19.48	± 0.493	+ 63.77	- 38.94	46.1 to 102.3	-50.6 to -31.6
	Middle	SUV_{max}	16.88	± 0.432	+ 54.09	- 35.10	39.4 to 85.4	-61.7 to -28.3
		SUV_{mean}	15.10	± 0.390	+ 47.68	- 32.29	34.9 to 74.6	-42.7 to -25.9
		SUV_{peak}	18.98	± 0.482	+ 61.88	- 38.23	44.9 to 99.1	-49.8 to -30.9
	Lower	SUV_{max}	18.85	± 0.479	+ 61.38	- 38.04	44.6 to 98.3	-49.6 to -30.8
		SUV_{mean}	18.48	± 0.470	+ 60.01	- 37.51	43.6 to 95.7	-48.9 to -30.3
		SUV_{peak}	15.40	± 0.397	+ 48.73	- 32.77	35.7 to 76.4	-43.3 to -26.3

wCV within-subject coefficient of variation, *RC_{ln}* repeatability coefficient on logarithmic scale, *SUV* standardized uptake value, *values were calculated after removing extreme outlier, values in parentheses were calculated from original scale

Table S4 Mean and SD of observer 1 and observer 2 with their absolute, original and log-transformed differences

Healthy Tissue		PET parameter	Observer 1	Observer 2	$\lvert d \rvert$	d	p value [a]	p value [b]	p value [c]	d_{ln}	p value [d]	p value [e]	p value [f]
**Mediastinal blood pool**		SUV_{max}	2.77 ± 0.31	2.85 ± 0.57	0.33 ± 0.30	0.08 ± 0.45	*0.003***	*0.414*	*0.961*	0.02 ± 0.15	*0.012**	*0.617*	*0.935*
		SUV_{mean}	1.99 ± 0.25	2.00 ± 0.33	0.13 ± 0.12	0.01 ± 0.18	*0.334*	*0.771*	*0.884*	0 ± 0.08	*0.936*	*0.970*	*0.935*
		SUV_{peak}	2.29 ± 0.26	2.30 ± 0.36	0.13 ± 0.12	0.01 ± 0.18	*0.794*	*0.707*	*0.808*	0 ± 0.07	*0.838*	*0.966*	*0.910*
**Left ventricle**		SUV_{max}	9.08 ± 5.90	9.22 ± 6.34	1.29 ± 1.50	0.15 ± 2.00	*0.035**	*0.763*	*0.397*	-0.02 ± 0.26	*0.002***	*0.777*	*0.510*
		SUV_{mean}	5.84 ± 3.93	5.56 ± 4.07	0.67 ± 1.00	-0.28 ± 1.18	*<0.001****	*0.342*	*0.943*	-0.09 ± 0.24	*<0.001****	*0.156*	*0.723*
		SUV_{peak}	6.67 ± 4.57	6.80 ± 4.87	0.83 ± 1.32	0.13 ± 1.57	*0.001***	*0.737*	*0.363*	-0.01 ± 0.22	*0.004***	*0.847*	*0.510*
**Bone marrow**		SUV_{max}	3.0 ± 0.83	2.99 ± 0.82	0.14 ± 0.12	-0.01 ± 0.19	*0.097*	*0.765*	*0.654*	0 ± 0.07	*0.135*	*0.913*	*0.794*
		SUV_{mean}	2.04 ± 0.64	2.00 ± 0.63	0.11 ± 0.13	-0.04 ± 0.17	*<0.001****	*0.276*	*0.884*	-0.02 ± 0.10	*<0.001****	*0.340*	*0.961*
		SUV_{peak}	2.37 ± 0.62	2.37 ± 0.62	0.06 ± 0.07	0 ± 0.09	*0.053*	*0.873*	*0.852*	0 ± 0.04	*0.086*	*0.885*	*0.823*
**Skeletal muscle**	Right	SUV_{max}	1.03 ± 0.25	1.12 ± 0.26	0.20 ± 0.16	0.09 ± 0.24	*0.558*	*0.084*	*0.123*	0.09 ± 0.23	*0.391*	*0.082*	*0.108*
		SUV_{mean}	0.67 ± 0.15	0.65 ± 0.13	0.07 ± 0.06	-0.02 ± 0.09	*0.119*	*0.279*	*0.131*	-0.03 ± 0.13	*0.065*	*0.328*	*0.178*
		SUV_{peak}	0.81 ± 0.16	0.81 ± 0.17	0.09 ± 0.07	0 ± 0.11	*0.164*	*0.883*	*0.783*	0 ± 0.14	*0.164*	*0.869*	*0.858*
	Left	SUV_{max}	1.07 ± 0.21	1.06 ± 0.19	0.13 ± 0.12	-0.01 ± 0.18	*0.083*	*0.760*	*0.884*	-0.01 ± 0.17	*0.261*	*0.813*	*0.935*
		SUV_{mean}	0.68 ± 0.13	0.64 ± 0.11	0.07 ± 0.06	-0.04 ± 0.08	*0.352*	*0.050*	*0.020**	-0.05 ± 0.11	*0.503*	*0.038**	*0.024**
		SUV_{peak}	0.80 ± 0.13	0.77 ± 0.12	0.06 ± 0.05	-0.03 ± 0.07	*0.717*	*0.079*	*0.101*	-0.04 ± 0.09	*0.773*	*0.092*	*0.131*
**Right lung**	Upper	SUV_{max}	1.17 ± 0.28	1.11 ± 0.31	0.29 ± 0.21	-0.06 ± 0.36	*0.199*	*0.629*	*0.629*	-0.08 ± 0.40	*0.017**	*0.392*	*0.687*
		SUV_{mean}	0.53 ± 0.16	0.53 ± 0.16	0.04 ± 0.03	-0.02 ± 0.05	*0.395*	*0.112*	*0.126*	-0.04 ± 0.14	*0.517*	*0.202*	*0.314*
		SUV_{peak}	0.98 ± 0.26	0.94 ± 0.32	0.24 ± 0.25	-0.04 ± 0.35	*0.056*	*0.642*	*0.968*	-0.07 ± 0.44	*0.007***	*0.477*	*0.936*
	Middle	SUV_{max}	1.49 ± 0.43	1.37 ± 0.53	0.38 ± 0.32	-0.12 ± 0.49	*0.725*	*0.250*	*0.291*	-0.11 ± 0.32	*0.555*	*0.127*	*0.223*
		SUV_{mean}	0.47 ± 0.13	0.47 ± 0.14	0.03 ± 0.03	0 ± 0.05	*0.081*	*0.635*	*0.485*	-0.02 ± 0.10	*0.682*	*0.421*	*0.372*
		SUV_{peak}	1.31 ± 0.35	1.20 ± 0.44	0.28 ± 0.23	-0.11 ± 0.34	*0.515*	*0.139*	*0.178*	-0.12 ± 0.31	*0.852*	*0.090*	*0.131*
	Lower	SUV_{max}	1.60 ± 0.56	1.63 ± 0.54	0.20 ± 0.16	0.03 ± 0.26	*0.710*	*0.547*	*0.543*	0.02 ± 0.21	*0.325*	*0.596*	*0.394*
		SUV_{mean}	0.53 ± 0.16	0.53 ± 0.16	0.03 ± 0.02	0 ± 0.04	*0.634*	*0.699*	*0.758*	-0.01 ± 0.08	*0.634*	*0.655*	*0.758*
		SUV_{peak}	1.34 ± 0.47	1.35 ± 0.48	0.18 ± 0.22	0.02 ± 0.29	*0.021**	*0.775*	*0.638*	0 ± 0.28	*0.001***	*0.912*	*0.570*
**Left lung**	Upper	SUV_{max}	1.15 ± 0.35	1.12 ± 0.31	0.28 ± 0.21	-0.03 ± 0.35	*0.268*	*0.703*	*0.478*	-0.02 ± 0.28	*0.164*	*0.803*	*0.709*
		SUV_{mean}	0.41 ± 0.10	0.40 ± 0.09	0.04 ± 0.03	-0.01 ± 0.05	*0.566*	*0.361*	*0.455*	-0.01 ± 0.05	*0.307*	*0.479*	*0.478*
		SUV_{peak}	0.97 ± 0.27	0.91 ± 0.19	0.19 ± 0.13	-0.06 ± 0.22	*0.365*	*0.251*	*0.391*	-0.05 ± 0.24	*0.277*	*0.383*	*0.526*
	Middle	SUV_{max}	1.40 ± 0.41	1.31 ± 0.41	0.30 ± 0.21	-0.09 ± 0.36	*0.871*	*0.283*	*0.263*	-0.07 ± 0.27	*0.929*	*0.261*	*0.218*
		SUV_{mean}	0.45 ± 0.12	0.45 ± 0.11	0.02 ± 0.02	0 ± 0.03	*0.787*	*0.501*	*0.502*	-0.01 ± 0.07	*0.908*	*0.444*	*0.024**
		SUV_{peak}	1.25 ± 0.37	1.11 ± 0.29	0.25 ± 0.23	-0.13 ± 0.32	*0.394*	*0.075*	*0.086*	-0.12 ± 0.26	*0.830*	*0.075*	*0.073*
	Lower	SUV_{max}	1.56 ± 0.42	1.44 ± 0.37	0.24 ± 0.25	-0.12 ± 0.32	*0.035**	*0.091*	*0.159*	-0.03 ± 0.21	*0.301*	*0.081*	*0.149*
		SUV_{mean}	0.59 ± 0.19	0.58 ± 0.18	0.03 ± 0.02	-0.01 ± 0.03	*0.558*	*0.152*	*0.131*	-0.02 ± 0.07	*0.099*	*0.250*	*0.289*
		SUV_{peak}	1.30 ± 0.41	1.17 ± 0.33	0.24 ± 0.31	-0.13 ± 0.38	*0.003***	*0.138*	*0.192*	-0.10 ± 0.26	*0.044**	*0.103*	*0.159*

SUV standardized uptake value, $\lvert d \rvert$ absolute difference of |observer2 – observer1|, d difference of original values (observer2 – observer1) with the signs (+/-), [a]*p*-value of d from Shapiro-Wilk test, [b]*p*-value of d from paired sample t-test, [c]*p*-value of d from Wilcoxon signed rank test, d_{ln} log-transformed difference [ln(observer2) – ln(observer1)], [d]*p*-value of d_{ln} from Shapiro-Wilk test, [e]*p*-value of d_{ln} from paired sample t-test, [f]*p*-value of d_{ln} from Wilcoxon signed rank test, ***p* ≤0.05, ****p* ≤0.01 and *****p* ≤0.001

Table S5 Correlations between the difference of interobserver data and the means of SUVs (maximum, mean and peak)

PET parameter		Difference	Kendall τ				Pearson	
			Absolute difference	Absolute Log-difference	Original difference	Log-difference	Original difference	Log-difference
**Mediastinal blood pool**		SUV_{max}	(0.342, *p*= 0.026*)	(0.238, *p*= 0.121)	(0.299, *p*= 0.052)	(0.377, *p*= 0.014*)	(0.633, *p*= 0.002**)	(0.613, *p*= 0.002**)
		SUV_{mean}	(0.022, *p*= 0.888)	(-0.056, *p*= 0.714)	(0.307, *p*= 0.045*)	(0.299, *p*= 0.052)	(0.458, *p*= 0.032*)	(0.401, *p*= 0.064)
		SUV_{peak}	(0.273, *p*= 0.076)	(0.221, *p*= 0.150)	(0.333, *p*= 0.030*)	(0.333, *p*= 0.030*)	(0.583, *p*= 0.004**)	(0.601, *p*= 0.003**)
**Left ventricle**		SUV_{max}	(0.245, *p*= 0.173)	(-0.052, *p*= 0.772)	(0.290, *p*= 0.107)	(0.156, *p*= 0.386)	(0.219, *p*= 0.398)	(0.287, *p*= 0.263)
		SUV_{mean}	(0.103, *p*= 0.564)	(-0.191, *p*= 0.284)	(0.279, *p*= 0.118)	(0.235, *p*= 0.187)	(0.118, *p*= 0.652)	(0.344, *p*= 0.176)
		SUV_{peak}	(0.230, *p*= 0.200)	(-0.112, *p*= 0.535)	(0.260, *p*= 0.148)	(0.067, *p*= 0.710)	(0.195, *p*= 0.452)	(0.257, *p*= 0.319)
**Bone marrow**		SUV_{max}	(-0.087, *p*= 0.573)	(-0.295, *p*= 0.055)	(0.000, *p*= 1.000)	(-0.026, *p*= 0.866)	(-0.059, *p*= 0.795)	(-0.194, *p*= 0.387)
		SUV_{mean}	(0.022, *p*= 0.888)	(-0.134, *p*= 0.382)	(-0.100, *p*= 0.517)	(-0.134, *p*= 0.382)	(-0.015, *p*= 0.949)	(-0.031, *p*= 0.892)
		SUV_{peak}	(-0.113, *p*= 0.463)	(-0.200, *p*= 0.194)	(0.113, *p*= 0.463)	(0.121, *p*= 0.430)	(0.033, *p*= 0.885)	(-0.020, *p*= 0.928)
**Skeletal muscle**	Right	SUV_{max}	(0.126, *p*= 0.414)	(0.022, *p*= 0.888)	(-0.065, *p*= 0.672)	(-0.100, *p*= 0.517)	(0.073, *p*= 0.748)	(-0.053, *p*= 0.815)
		SUV_{mean}	(0.472, *p*= 0.002**)	(0.333, *p*= 0.030*)	(-0.368, *p*= 0.017*)	(-0.307, *p*= 0.045*)	(-0.206, *p*= 0.358)	(-0.214, *p*= 0.338)
		SUV_{peak}	(0.368, *p*= 0.017*)	(0.273, *p*= 0.076)	(-0.126, *p*= 0.414)	(-0.108, *p*= 0.481)	(0.022, *p*= 0.921)	(-0.034, *p*= 0.880)
	Left	SUV_{max}	(-0.212, *p*= 0.167)	(-0.307, *p*= 0.045*)	(-0.091, *p*= 0.554)	(-0.082, *p*= 0.592)	(-0.146, *p*= 0.516)	(-0.070, *p*= 0.756)
		SUV_{mean}	(0.342, *p*= 0.026*)	(0.212, *p*= 0.167)	(-0.186, *p*= 0.225)	(-0.108, *p*= 0.481)	(-0.224, *p*= 0.316)	(-0.083, *p*= 0.714)
		SUV_{peak}	(0.091, *p*= 0.554)	(-0.048, *p*= 0.756)	(-0.212, *p*= 0.167)	(-0.143, *p*= 0.352)	(-0.249, *p*= 0.264)	(-0.131, *p*= 0.563)
**Right lung**	Upper	SUV_{max}	(-0.053, *p*= 0.753)	(-0.158, *p*= 0.345)	(-0.064, *p*= 0.700)	(-0.006, *p*= 0.972)	(0.086, *p*= 0.728)	(0.466, *p*= 0.045*)
		SUV_{mean}	(0.205, *p*= 0.221)	(-0.216, *p*= 0.196)	(-0.251, *p*= 0.132)	(-0.170, *p*= 0.310)	(-0.281, *p*= 0.243)	(0.003, *p*= 0.992)
		SUV_{peak}	(0.041, *p*= 0.807)	(-0.123, *p*= 0.463)	(0.193, *p*= 0.248)	(0.275, *p*= 0.100)	(0.223, *p*= 0.359)	(0.396, *p*= 0.093)
	Middle	SUV_{max}	(0.299, *p*= 0.052)	(0.039, *p*= 0.800)	(0.048, *p*= 0.756)	(0.126, *p*= 0.414)	(0.249, *p*= 0.264)	(0.204, *p*= 0.362)
		SUV_{mean}	(-0.056, *p*=0.714)	(-0.229, *p*= 0.135)	(0.169, *p*= 0.271)	(0.203, *p*= 0.185)	(0.215, *p*= 0.336)	(0.265, *p*= 0.234)
		SUV_{peak}	(-0.229, *p*= 0.135)	(-0.429, *p*= 0.005**)	(0.203, *p*= 0.185)	(0.238, *p*= 0.121)	(0.271, *p*= 0.223)	(0.338, *p*= 0.124)
	Lower	SUV_{max}	(-0.273, *p*= 0.076)	(-0.498, *p*= 0.001***)	(-0.134, *p*= 0.382)	(-0.160, *p*= 0.297)	(-0.096, *p*= 0.670)	(0.001, *p*= 0.998)
		SUV_{mean}	(-0.203, *p*= 0.185)	(-0.316, *p*= 0.040*)	(0.030, *p*= 0.844)	(0.056, *p*= 0.714)	(0.058, *p*= 0.797)	(0.121, *p*= 0.593)
		SUV_{peak}	(-0.091, *p*= 0.554)	(-0.238, *p*= 0.121)	(0.004, *p*= 0.978)	(-0.048, *p*= 0.756)	(0.064, *p*= 0.778)	(0.106, *p*= 0.639)
**Left lung**	Upper	SUV_{max}	(0.263, *p*= 0.105)	(0.032, *p*= 0.846)	(-0.158, *p*= 0.330)	(-0.126, *p*= 0.436)	(-0.158, *p*= 0.505)	(-0.174, *p*= 0.464)
		SUV_{mean}	(0.158, *p*= 0.330)	(0.032, *p*= 0.846)	(-0.200, *p*= 0.218)	(-0.189, *p*= 0.243)	(-0.356, *p*= 0.123)	(-0.251, *p*= 0.287)
		SUV_{peak}	(0.105, *p*= 0.516)	(-0.211, *p*= 0.194)	(-0.200, *p*= 0.218)	(-0.179, *p*= 0.270)	(-0.396, *p*= 0.084)	(-0.291, *p*= 0.213)
	Middle	SUV_{max}	(0.253, *p*= 0.119)	(0.032, *p*= 0.846)	(-0.053, *p*= 0.746)	(0.011, *p*= 0.948)	(-0.009, *p*= 0.969)	(0.037, *p*= 0.878)
		SUV_{mean}	(-0.126, *p*= 0.436)	(-0.295, *p*= 0.069)	(0.105, *p*= 0.516)	(0.105, *p*= 0.516)	(0.130, *p*= 0.586)	(0.162, *p*= 0.496)
		SUV_{peak}	(0.211, *p*= 0.194)	(0.105, *p*= 0.516)	(-0.032, *p*= 0.846)	(0.011, *p*= 0.948)	(-0.268, *p*= 0.252)	(-0.094, *p*= 0.694)
	Lower	SUV_{max}	(0.019, *p*= 0.904)	(-0.143, *p*= 0.365)	(0.076, *p*= 0.629)	(0.114, *p*= 0.469)	(-0.164, *p*= 0.478)	(0.035, *p*= 0.881)
		SUV_{mean}	(0.095, *p*= 0.546)	(-0.190, *p*= 0.227)	(-0.133, *p*= 0.398)	(-0.086, *p*= 0.587)	(-0.157, *p*= 0.496)	(-0.076, *p*= 0.742)
		SUV_{peak}	(0.343, *p*= 0.030*)	(0.219, *p*= 0.165)	(-0.057, *p*= 0.717)	(0.000, *p*= 1.00)	(-0.242, *p*= 0.291)	(-0.124, *p*= 0.593)

SUV standardized uptake value, values in parentheses indicate (correlation coefficient, significance), *****$p \leq 0.05$, ******$p \leq 0.01$ and *******$p \leq 0.001$

Table S6 Within-subject coefficient of variation (wCV) and repeatability coefficients (RC) with 95%CIs for Inter-Observer Assessment of HT

Healthy tissue		PET parameter	wCV(%)	RC_{ln}	Upper RC (%)	Lower RC (%)	95% CI for URC	95% CI for LRC
**Mediastinal blood pool**		SUV_{max}	10.85	± 0.286	+ 33.06	- 24.85	24.6 to 50.5	-19.8 to -33.6
		SUV_{mean}	6.10	± 0.164	+ 17.84	- 15.14	13.4 to 26.4	-11.9 to -20.9
		SUV_{peak}	5.41	± 0.146	+ 15.72	- 13.58	11.9 to 23.2	-10.6 to -18.8
**Left ventricle**		SUV_{max}	20.29	± 0.512	+ 66.89	- 40.08	48.3 to 107.9	-32.6 to -51.9
		SUV_{mean}	21.61/14.00*	± 0.542/0.363*	+ 72.01/43.79*	- 41.87/30.46*	51.7 to 116.9/32.2 to 67.9*	- 53.9 to 34.1/-40.5 to -24.4*
		SUV_{peak}	16.78	± 0.430	+ 53.71	- 34.94	39.2 to 84.9	-28.2 to -45.9
**Bone marrow**		SUV_{max}	5.26	± 0.142	+ 15.26	- 13.24	11.6 to 22.5	-10.4 to -18.4
		SUV_{mean}	7.05/4.84*	± 0.189/0.131*	+ 20.77/14.01*	- 17.20/12.29*	15.7 to 31/10.6 to 20.6*	-13.5 to -23.7/-17.1 to -9.6*
		SUV_{peak}	3.06	± 0.083	+ 8.70	- 8.00	6.6 to 12.6	-6.2 to -11.2
**Skeletal muscle**	Right	SUV_{max}	17.26	± 0.441	+ 55.47	- 35.68	40.4 to 87.8	-46.8 to -28.8
		SUV_{mean}	9.94	± 0.263	+ 30.04	- 23.10	22.4 to 45.6	-31.3 to -18.3
		SUV_{peak}	10.28	± 0.271	+ 31.17	- 23.76	23.2 to 47.3	-32.1 to -18.8
	Left	SUV_{max}	12.66	± 0.330	+ 39.16	-28.14	28.9 to 60.3	-37.6 to -22.4
		SUV_{mean}	8.12	± 0.216	+ 24.16	- 19.46	18.1 to 36.2	-26.6 to -15.3
		SUV_{peak}	6.85	± 0.184	+ 20.16	- 16.78	15.2 to 30.1	-23.1 to -13.2
**Right lung**	Upper	SUV_{max}	33.10	± 0.792	+ 120.88	- 54.72	83.9 to 210.1	-67.8 to -45.6
		SUV_{mean}	10.52	± 0.277	+ 31.95	- 24.22	23.8 to 48.6	-32.7 to -19.2
		SUV_{peak}	36.47	± 0.862	+ 136.75	- 57.76	94.1 to 242.8	-70.8 to -48.5
	Middle	SUV_{max}	25.05	± 0.620	+ 85.83	- 46.19	61.1 to 142.5	-58.8 to -37.9
		SUV_{mean}	7.60	± 0.203	+ 22.48	- 18.35	16.9 to 33.7	-25.2 to -14.5
		SUV_{peak}	24.47	± 0.607	+ 83.44	-45.49	59.5 to 138.1	-57.9 to -37.3
	Lower	SUV_{max}	15.67	± 0.404	+ 49.71	-33.20	36.5 to 78.1	-43.9 to -26.7
		SUV_{mean}	6.12	± 0.165	+ 17.90	- 15.18	13.5 to 26.6	-21.0 to -11.9
		SUV_{peak}	21.90(20.21)	± 0.549(0.560)	+ 73.14(77.78)	- 42.24(-43.75)	52.6 to 119.1(43.1 to 80.0)	-54.4 to -34.5(-80.0 to -43.1)
**Left lung**	Upper	SUV_{max}	22.28	± 0.557	+ 74.63	- 42.74	53.5 to 121.7	-54.9 to -34.9
		SUV_{mean}	9.42	± 0.249	+ 28.35	- 22.09	21.1 to 42.7	-29.9 to -17.4
		SUV_{peak}	18.72	± 0.476	+ 60.89	- 37.85	43.2 to 97.4	-49.4 to -30.7
	Middle	SUV_{max}	20.85	± 0.525	+ 69.04	- 40.84	49.8 to 111.8	-52.8 to -33.2
		SUV_{mean}	4.78	± 0.129	+ 13.81	- 12.14	10.4 to 20.2	-16.8 to -9.5
		SUV_{peak}	20.17	± 0.509	+ 66.40	- 39.90	47.9 to 106.9	-51.7 to -32.4
	Lower	SUV_{max}	15.84	± 0.408	+ 50.31	- 33.47	36.9 to 79.2	-44.2 to -26.9
		SUV_{mean}	4.77	± 0.129	+ 13.78	- 12.11	10.4 to 20.2	-16.8 to -9.5
		SUV_{peak}	19.78	± 0.500	+ 64.92	- 39.37	46.9 to 104.3	-51.1 to -31.9

wCV within-subject coefficient of variation, RC_{ln} repeatability coefficient on logarithmic scale, *SUV* standardized uptake value, *values were calculated after removing extreme outlier, values in parentheses were calculated from original scale

Bland-Altman Plots for Test-retest Repeatability in each organ:

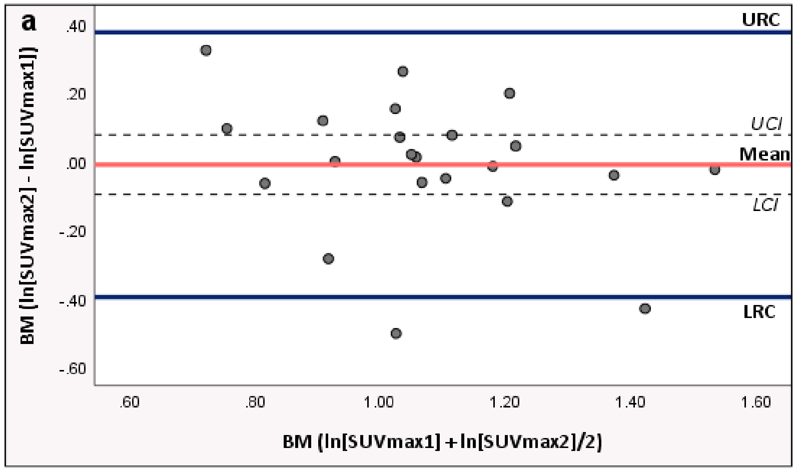

A simple linear regression indicates no significant bias (β = -0.294, p = 0.172).

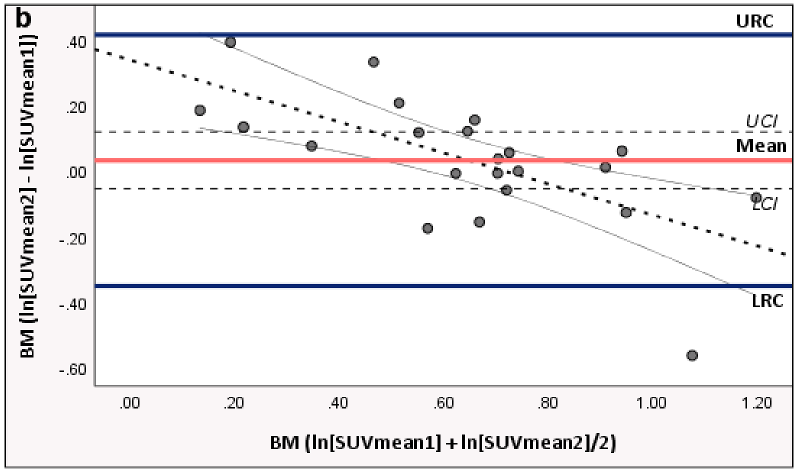

A simple linear regression (dotted line) indicates a significant bias (β = -0.475, p = *0.001*).

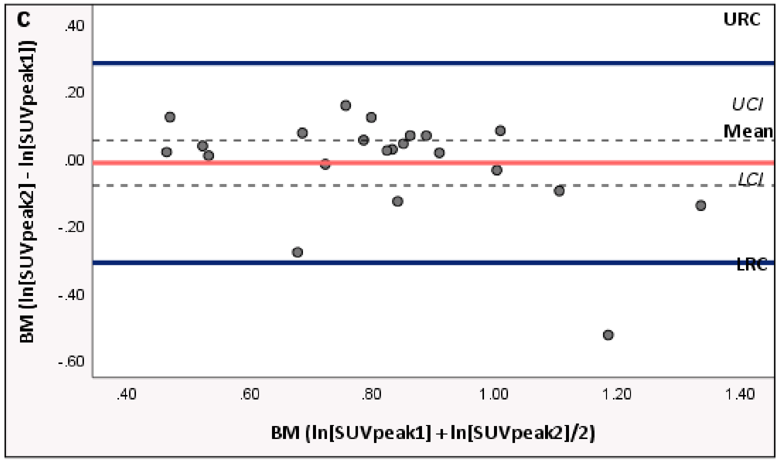

A simple linear regression (dotted line) indicates no significant bias (β = -0.309, p =*0.034*) **

URC/LRC: upper and lower repeatability coefficients. UCI/LCI: upper and lower 95% confidence intervals for the mean of the difference. SUV: standardized uptake value. BM: bone marrow.

**The 95% RCs for SUVpeak may not represent the true limits of agreement because both original and log-transformed differences were not normally distributed at significance level (p < 0.0015).

Fig. S1 Test-retest repeatability of FDG uptake in bone marrow (BM) illustrated by Bland-Altman method

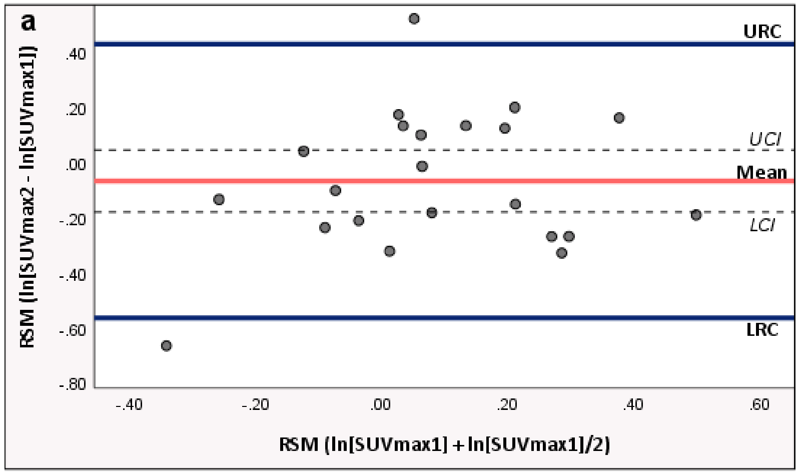

A simple linear regression indicates no significant bias (β = 0.254, p = 0.369).

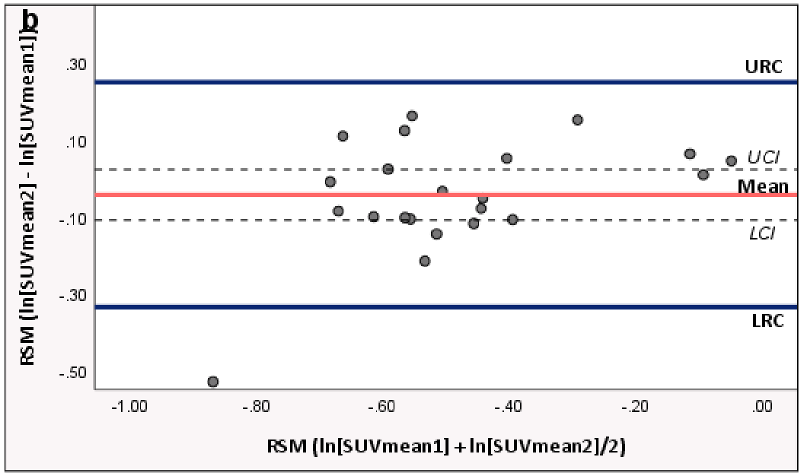

A simple linear regression (dotted line) indicates a significant bias (β = 0.340, p = *0.032*) **

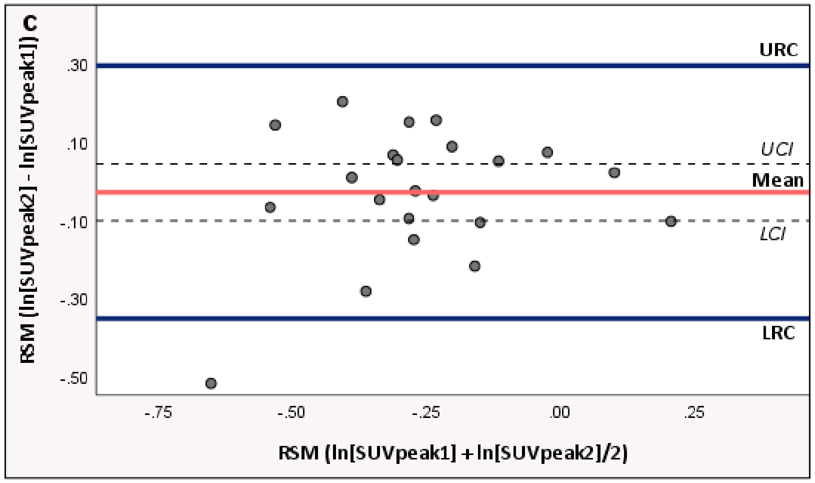

A simple linear regression indicates no significant bias (β = 0.169, p = 0.368) **

URC/LRC: upper and lower repeatability coefficients. UCI/LCI: upper and lower 95% confidence intervals for the mean of the difference. SUV: standardized uptake value. RSM: right skeletal muscle.

** The 95% RCs for SUVmean and SUVpeak may not represent the true limits of agreement because both original and log-transformed differences were not normally distributed at significance level (p < 0.0015).

Fig. S2 Test-retest repeatability of FDG uptake in right skeletal muscle (RSM) illustrated by Bland-Altman method

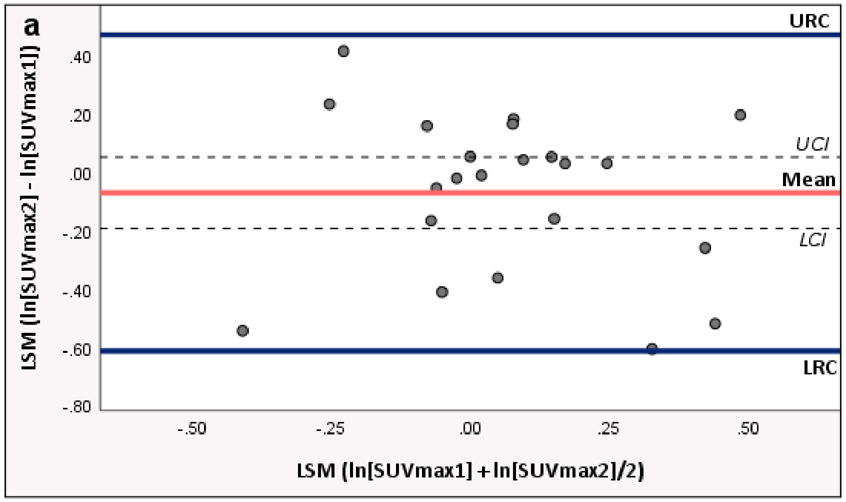

A simple linear regression indicates no significant bias (β = -0.216, *p* = 0.432).

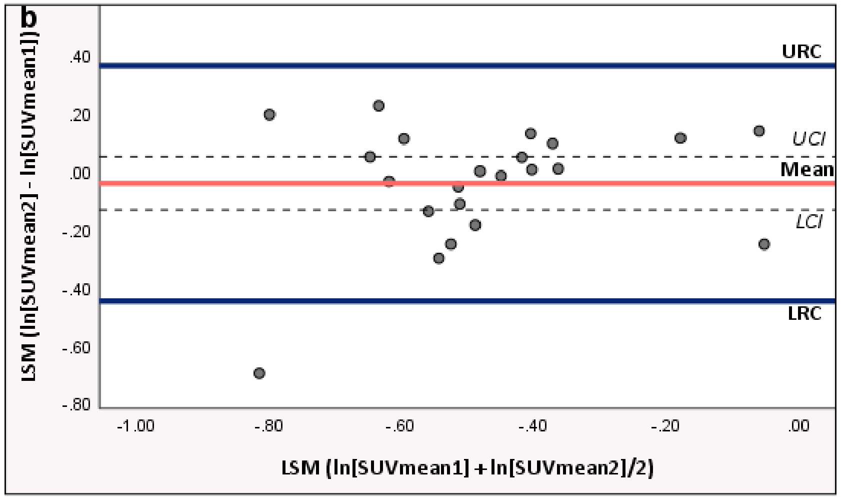

A simple linear regression indicates no significant bias (β = 0.226, *p* = 0.338).

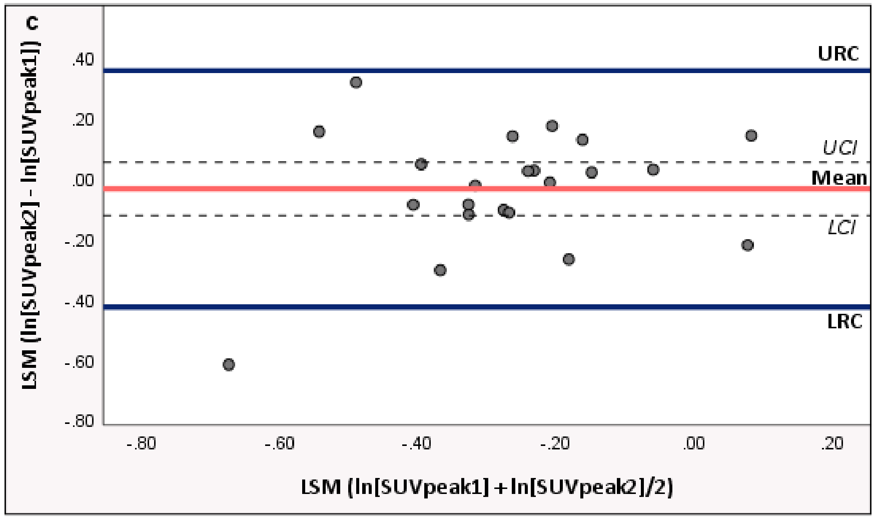

A simple linear regression indicates no significant bias (β = 0.254, *p* = 0.307).

URC/LRC: upper and lower repeatability coefficients. UCI/LCI: upper and lower 95% confidence intervals for the mean of the difference. SUV: standardized uptake value. LSM: left skeletal muscle.

Fig. S3 Test-retest repeatability of FDG uptake in left skeletal muscle (LSM) illustrated by Bland-Altman method

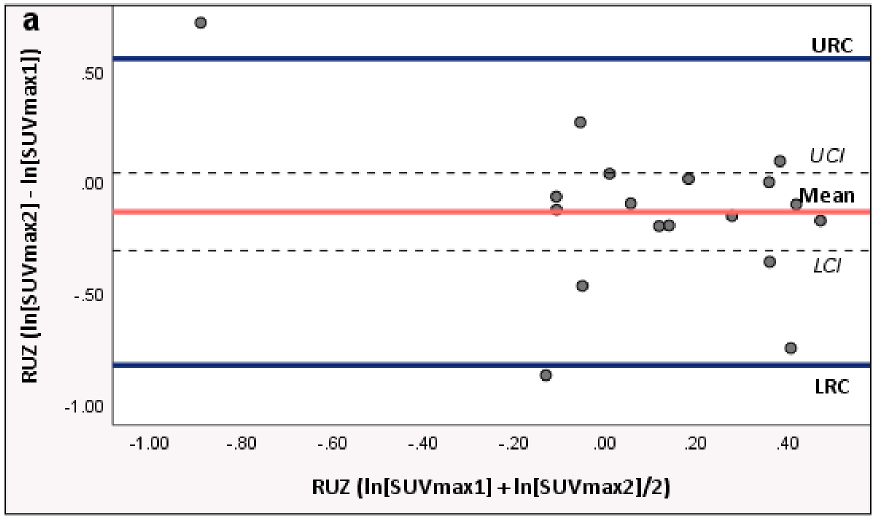

A simple linear regression indicates no significant bias (β = -0.489, *p* = 0.062).

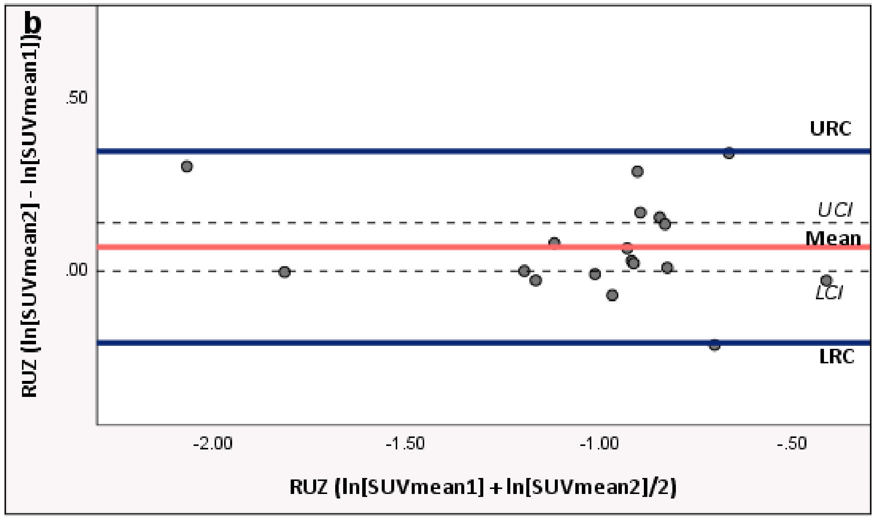

A simple linear regression indicates no significant bias (β = -0.070, *p* = 0.447).

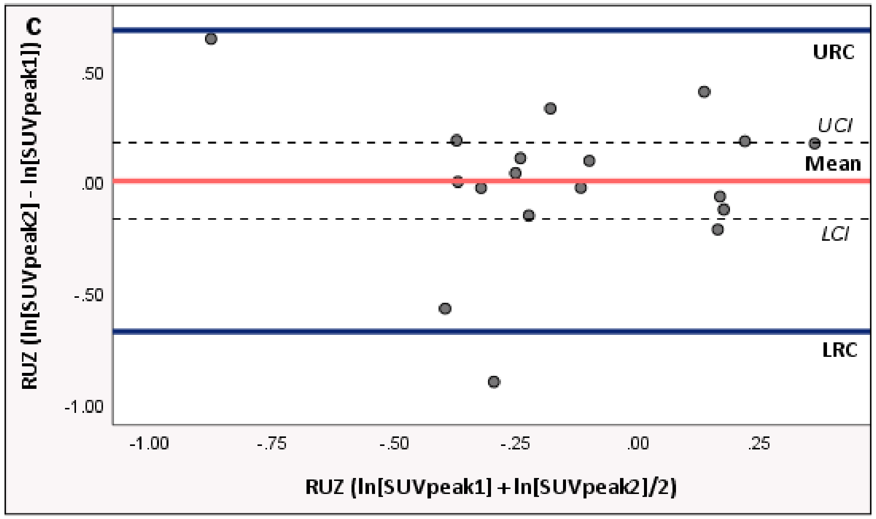

A simple linear regression indicates no significant bias (β = -0.064, *p* = 0.826).

URC/LRC: upper and lower repeatability coefficients. UCI/LCI: upper and lower 95% confidence intervals for the mean of the difference. SUV: standardized uptake value. RUZ: right upper lung zone.

Fig. S4 Test-retest repeatability of FDG uptake in right upper lung zone (RUZ) illustrated by Bland-Altman method.

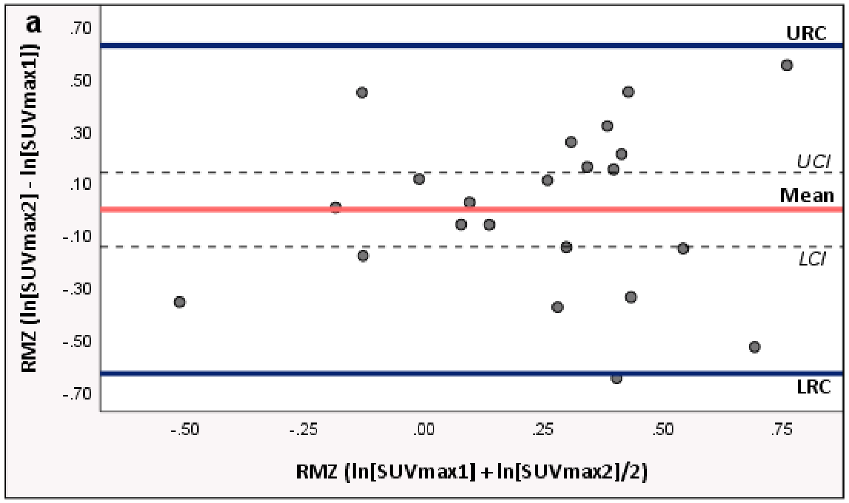

A simple linear regression indicates no significant bias (β = 0.096, p = 0.689).

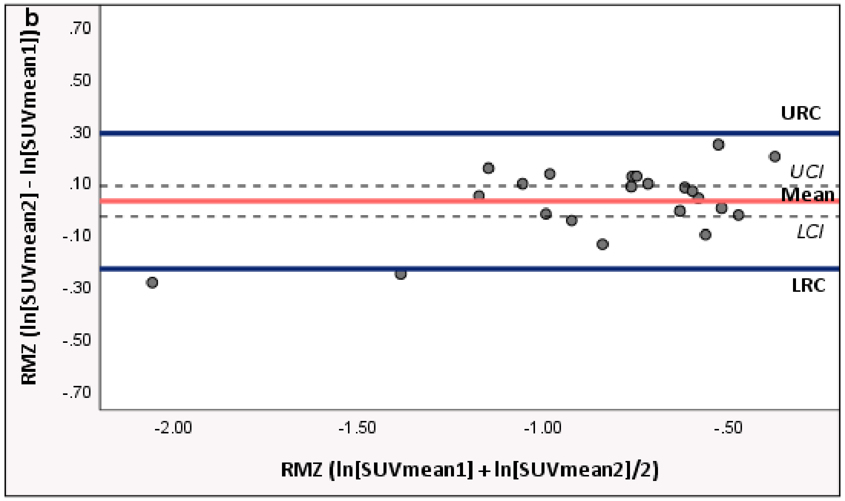

A simple linear regression (dotted line) indicates no significant bias (β = 0.202, p =*0.005*).

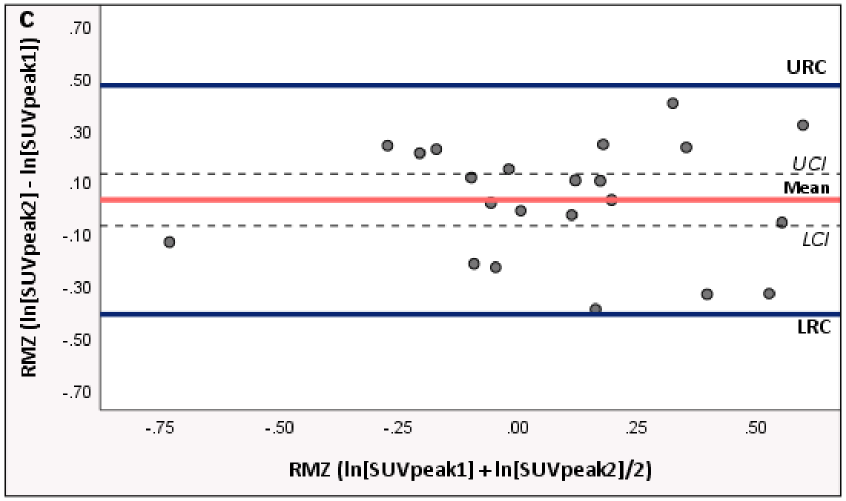

A simple linear regression indicates no significant bias (β = -0.009, p = 0.957).

URC/LRC: upper and lower repeatability coefficients. UCI/LCI: upper and lower 95% confidence intervals for the mean of the difference. SUV: standardized uptake value. RMZ: right middle lung zone.

Fig. S5 Test-retest repeatability of FDG uptake in right middle lung zone (RMZ) illustrated by Bland-Altman method.

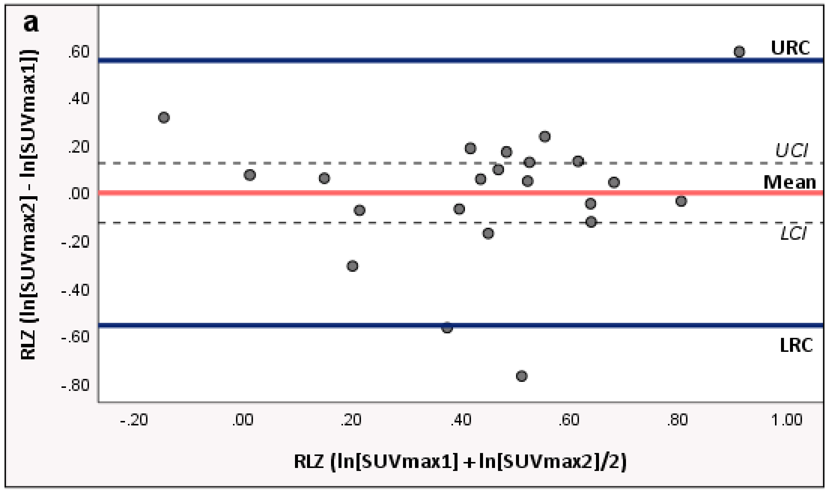

A simple linear regression indicates no significant bias (β = 0.119, p = 0.645).

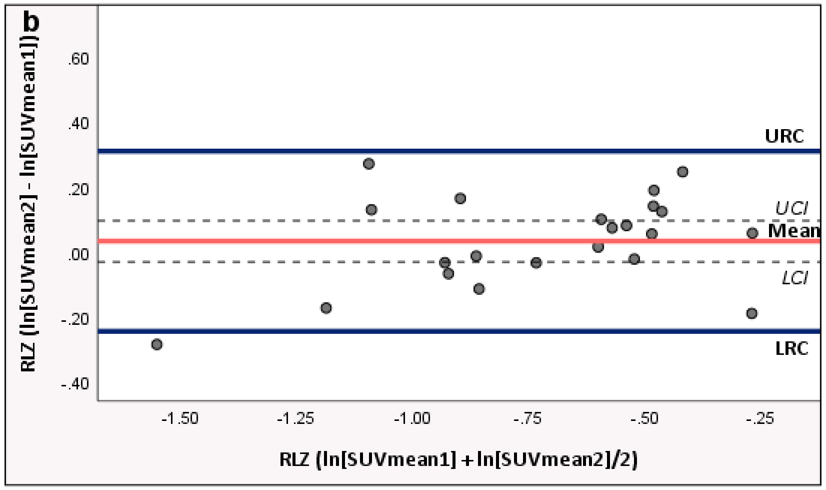

A simple linear regression indicates no significant bias (β = 0.159, p = 0.096).

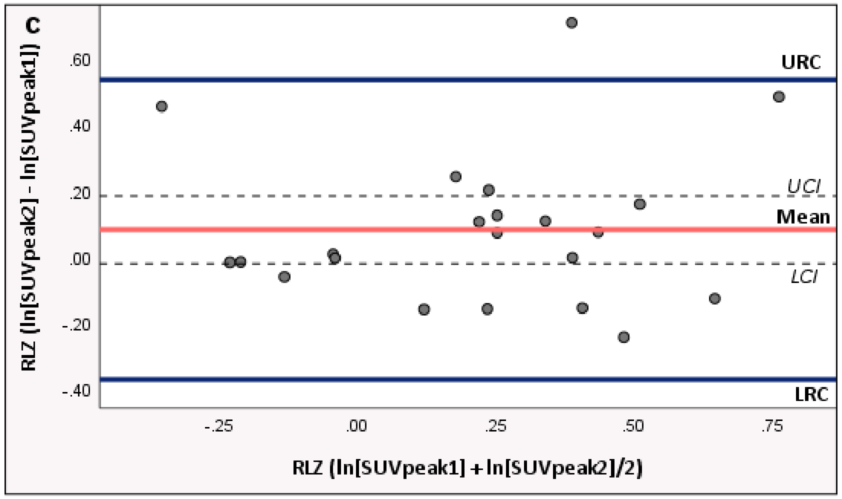

A simple linear regression indicates no significant bias (β = 0.054, p = 0.763).

URC/LRC: upper and lower repeatability coefficients. UCI/LCI: upper and lower 95% confidence intervals for the mean of the difference. SUV: standardized uptake value. RLZ: right lower lung zone.

Fig. S6 Test-retest repeatability of FDG uptake in right lower lung zone (RLZ) illustrated by Bland-Altman method.

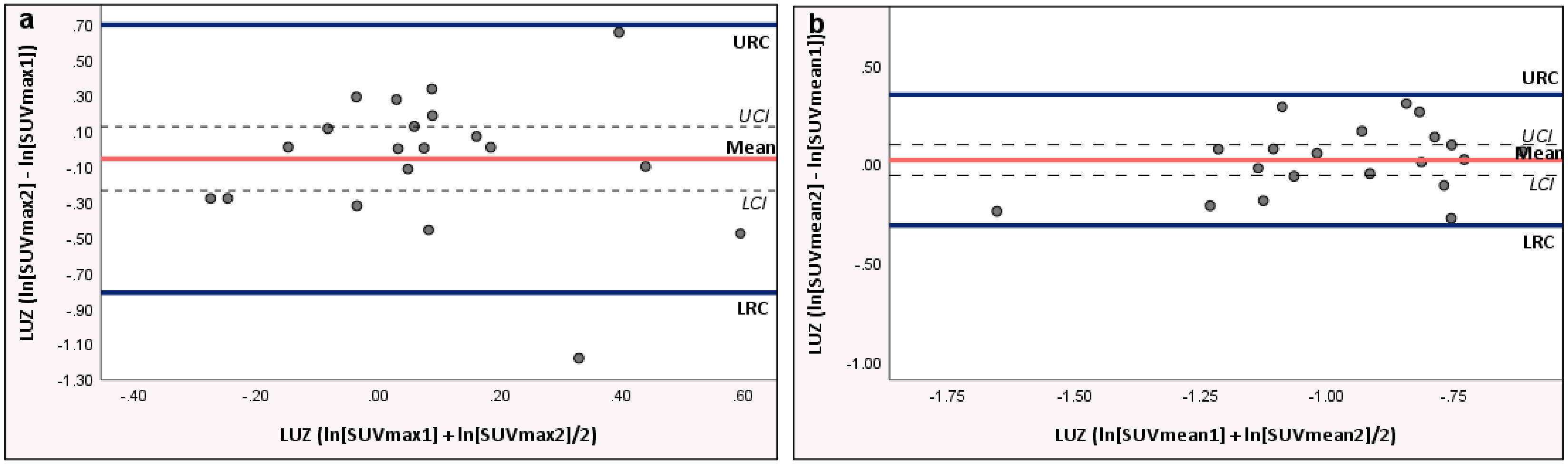

A simple linear regression indicates no significant bias (β = -0.206, *p* = 0.621).

A simple linear regression indicates no significant bias (β = -0.240, *p* = 0.137).

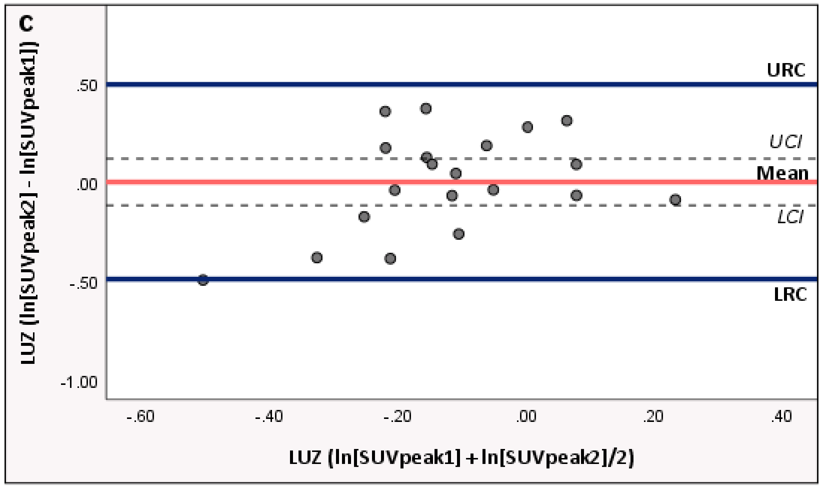

A simple linear regression indicates no significant bias (β = -0.647, *p* = 0.065).

URC/LRC: upper and lower repeatability coefficients. UCI/LCI: upper and lower 95% confidence intervals for the mean of the difference. SUV: standardized uptake value. LUZ: left upper lung zone.

Fig. S7 Test-retest repeatability of FDG uptake in left upper lung zone(LUZ) illustrated by Bland-Altman method.

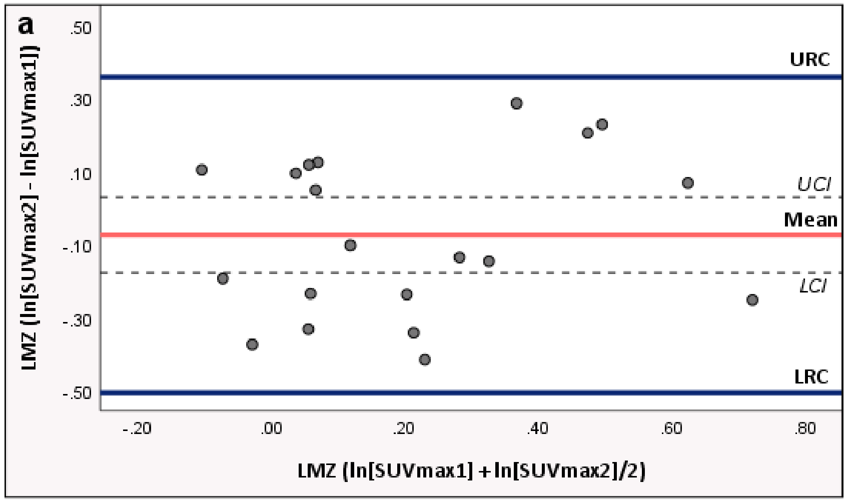

A simple linear regression indicates no significant bias (β = -0.175, *p* = 0.439).

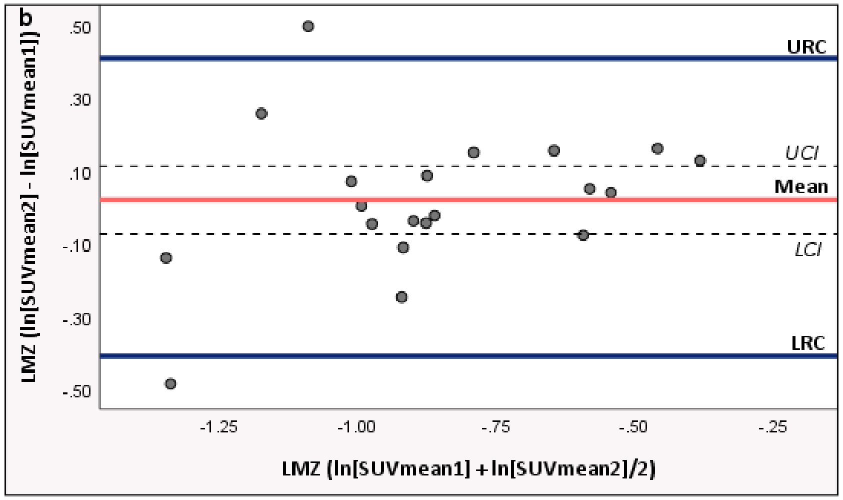

A simple linear regression indicates no significant bias (β = 218, *p* = 0.208).

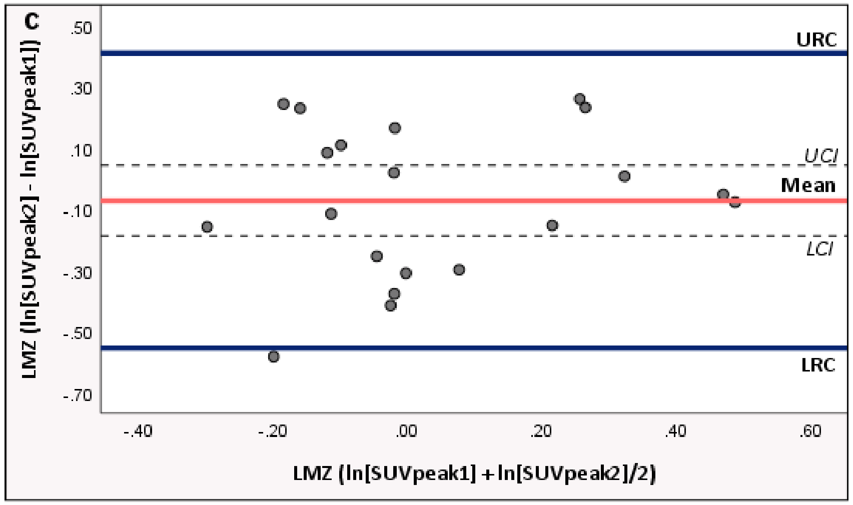

A simple linear regression indicates no significant bias (β = 179, *p* = 0.494).

URC/LRC: upper and lower repeatability coefficients. UCI/LCI: upper and lower 95% confidence intervals for the mean of the difference. SUV: standardized uptake value. LMZ: left middle lung zone.

Fig. S8 Test-retest repeatability of FDG uptake in left middle lung zone (LMZ) illustrated by Bland-Altman method.

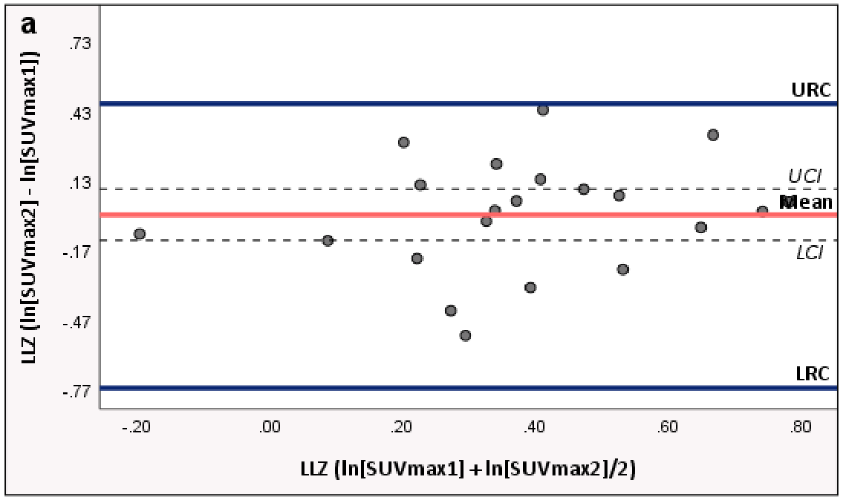

A simple linear regression indicates no significant bias (β = 222, *p* = 0.371).

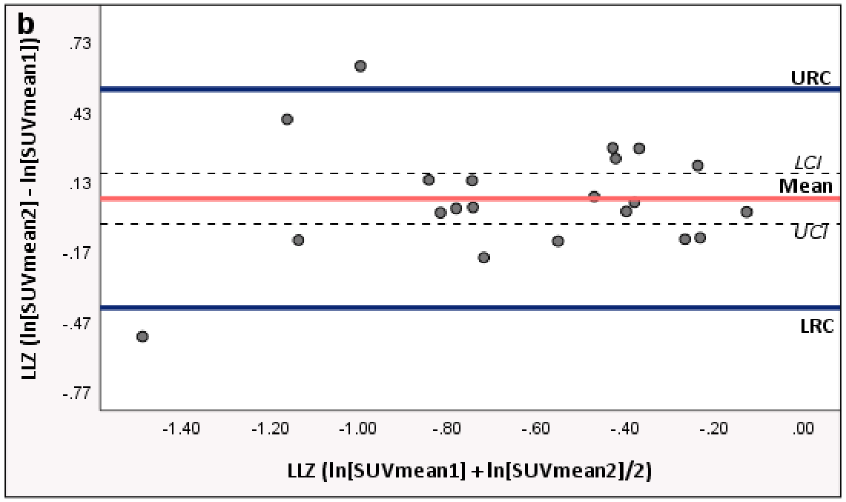

A simple linear regression indicates no significant bias (β = 090, *p* = 0.561).

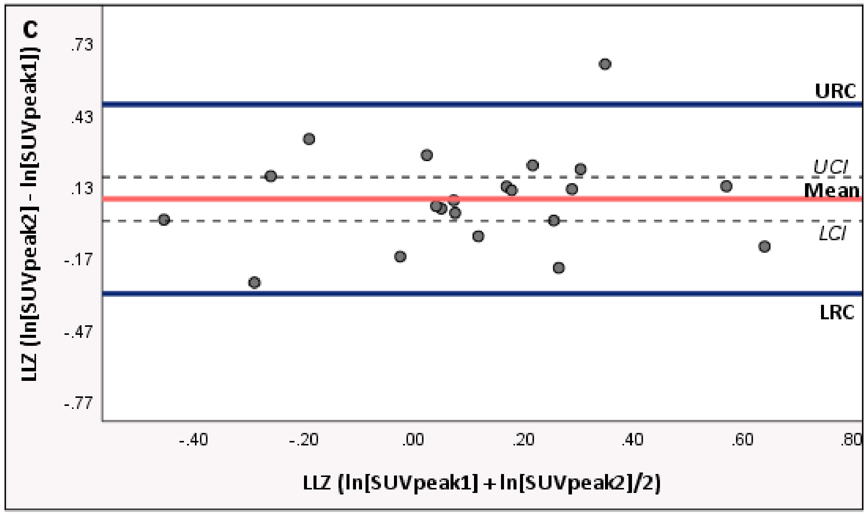

A simple linear regression indicates no significant bias (β = 105, *p* = 0.550).

URC/LRC: upper and lower repeatability coefficients. UCI/LCI: upper and lower 95% confidence intervals for the mean of the difference. SUV: standardized uptake value. LLZ: left lower lung zone.

Fig. S9 Test-retest repeatability of FDG uptake in left lower lung zone (LLZ) illustrated by Bland-Altman method.

Fig. S10 Distribution of different SUV parameters (maximum, mean and peak) for the test-retest data in each organ:

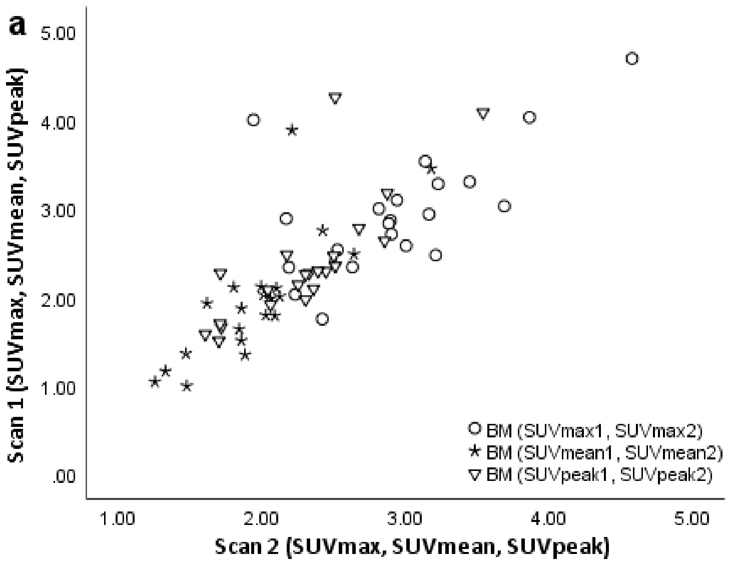

SUVmax, SUVmean and SUVpeak distribution in test-retest of bone marrow (BM)

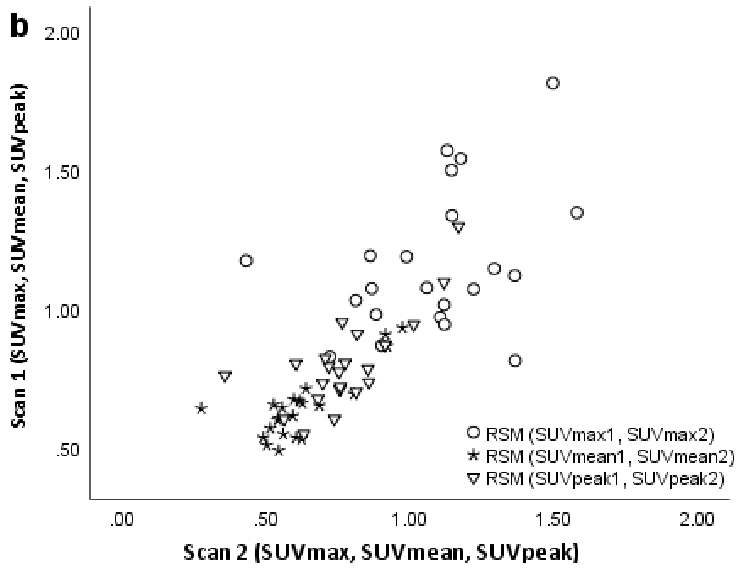

SUVmax, SUVmean and SUVpeak distribution in test-retest right skeletal muscle (RSM)

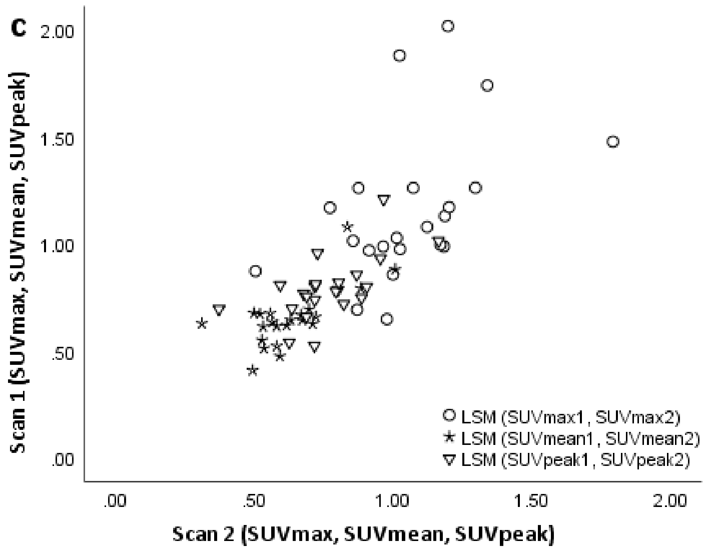

SUVmax, SUVmean and SUVpeak distribution in test-retest of left skeletal muscle.

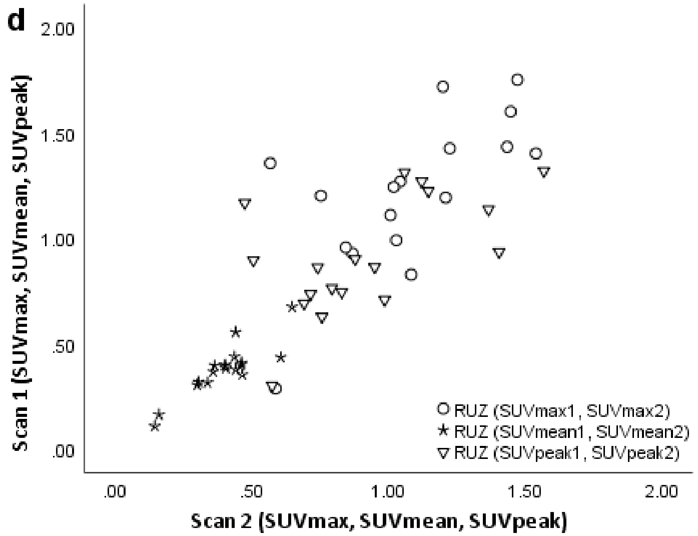

SUVmax, SUVmean and SUVpeak distribution in test-retest of right upper lung zone.

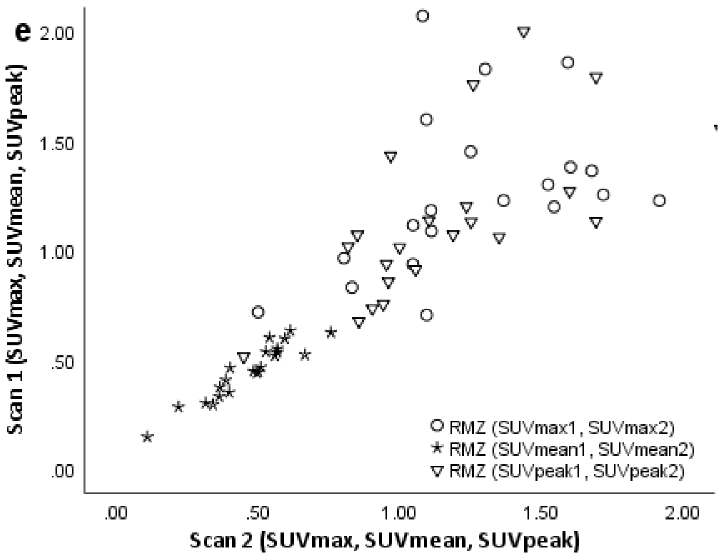

SUVmax, SUVmean and SUVpeak distribution in test-retest of right middle lung zone (RMZ)

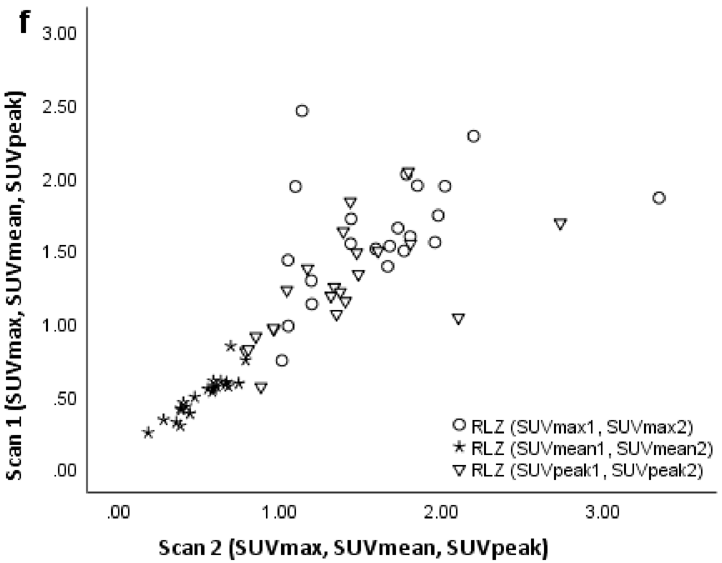

SUVmax, SUVmean and SUVpeak distribution in test-retest of right lower lung zone (RLZ)

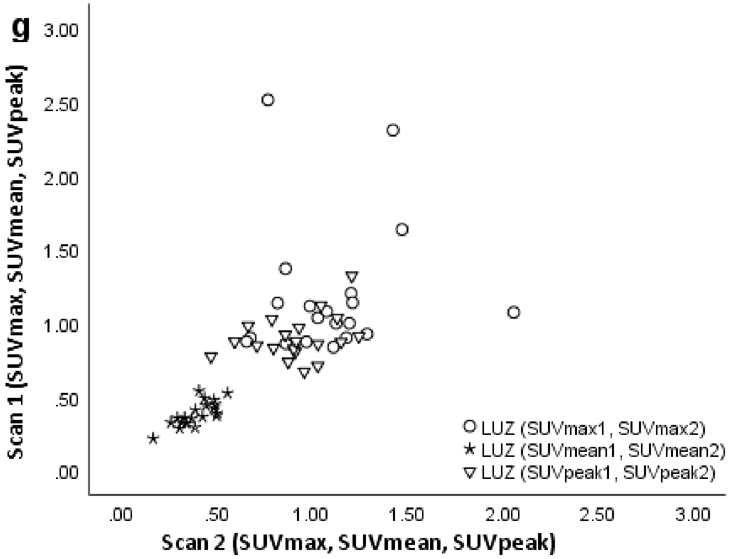

SUVmax, SUVmean and SUVpeak distribution in test-retest of left upper lung zone (LUZ)

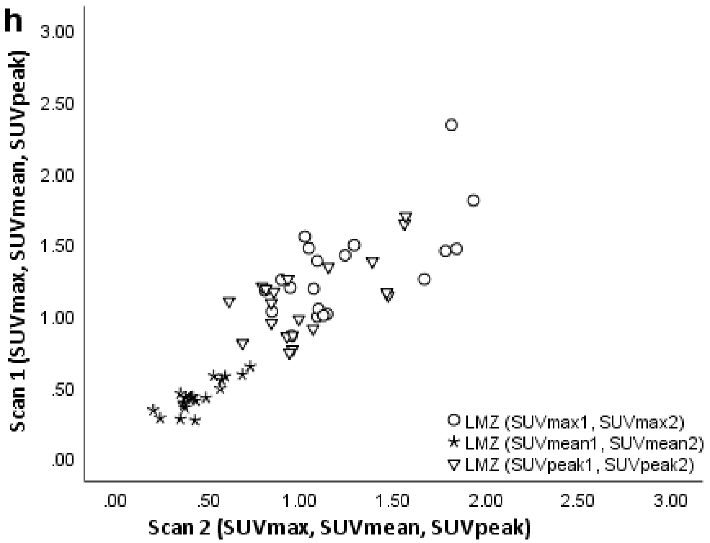

SUVmax, SUVmean and SUVpeak distribution in test-retest of left middle lung zone (LLZ)

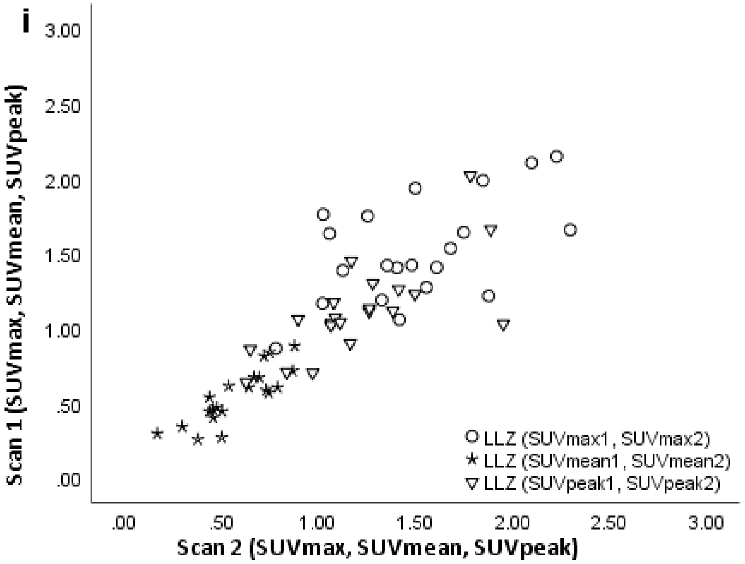

SUVmax, SUVmean and SUVpeak distribution in test-retest of left lower lung zone (LLZ)

Bland-Altman Plots for inter-observer reproducibility in each organ:

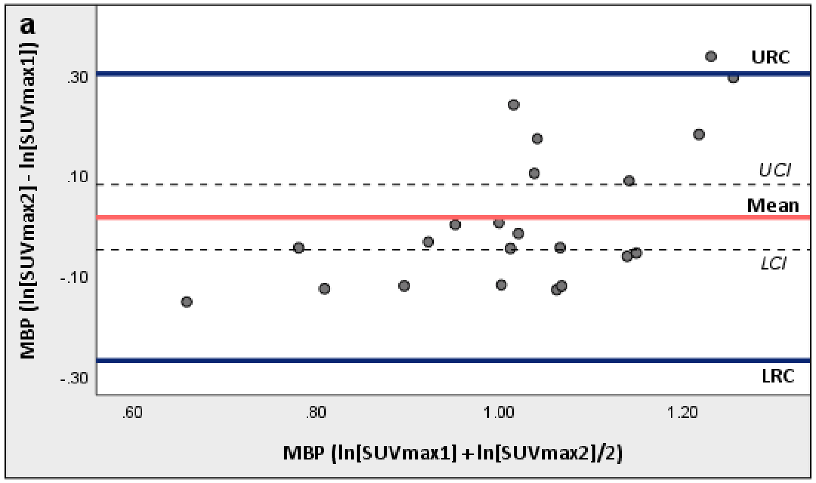

A simple linear regression (dotted line) indicates no significant bias (β = 0.609, *p = 0.002*).

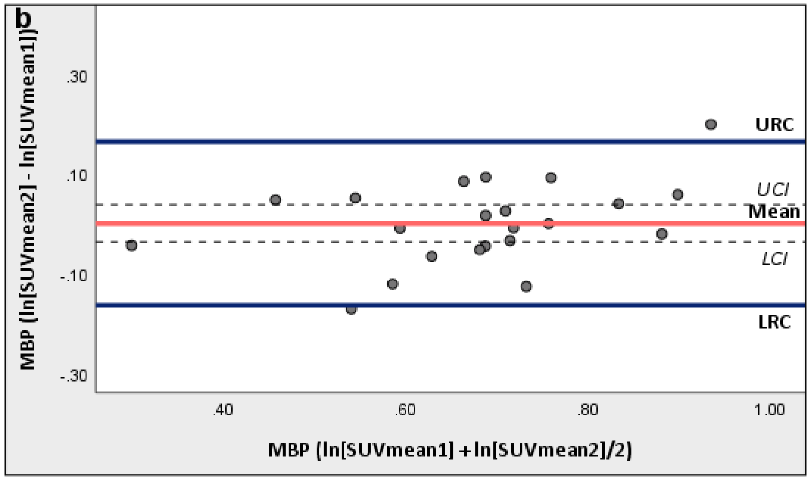

A simple linear regression indicates no significant bias (β = 0.231, *p* = 0.064).

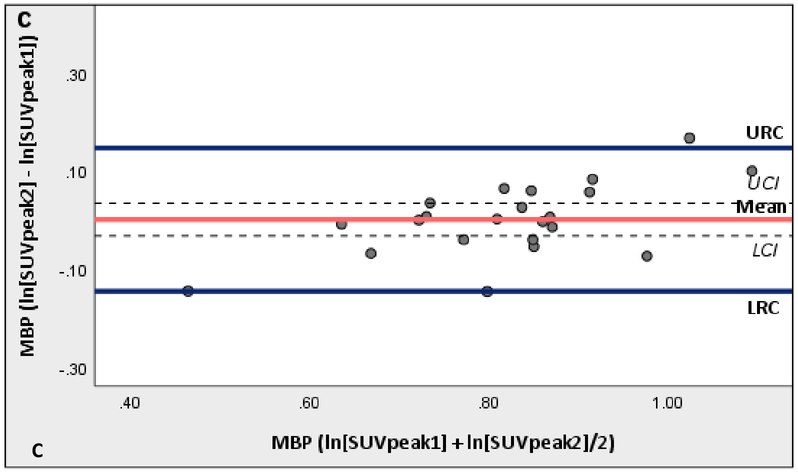

A simple linear regression (dotted line) indicates no significant bias (β = 0.334, *p = 0.003*).

URC/LRC: upper and lower repeatability coefficients. UCI/LCI: upper and lower 95% confidence intervals for the mean of the difference. SUV: standardized uptake value. MBP: mediastinal blood pool.

Fig. S11 Inter-observer repeatability of FDG uptake in mediastinal blood pool (MBP) illustrated by Bland-Altman method.

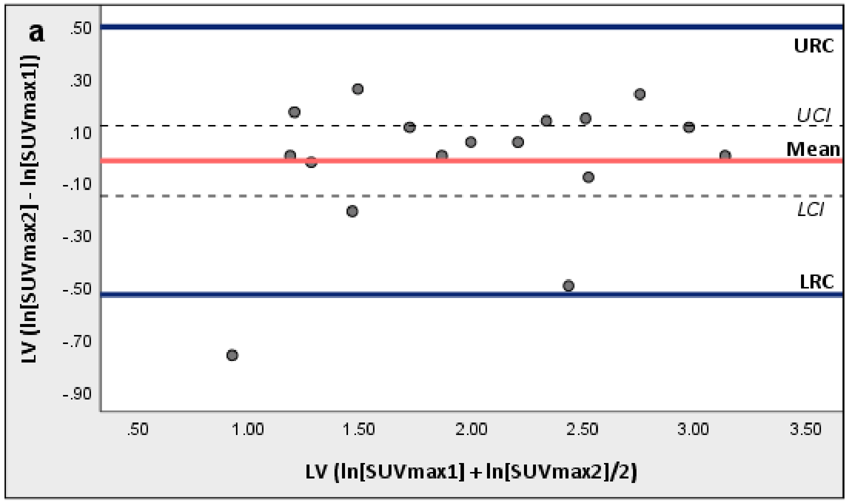

A simple linear regression indicates no significant bias (β = 0.111, p = 0.263).

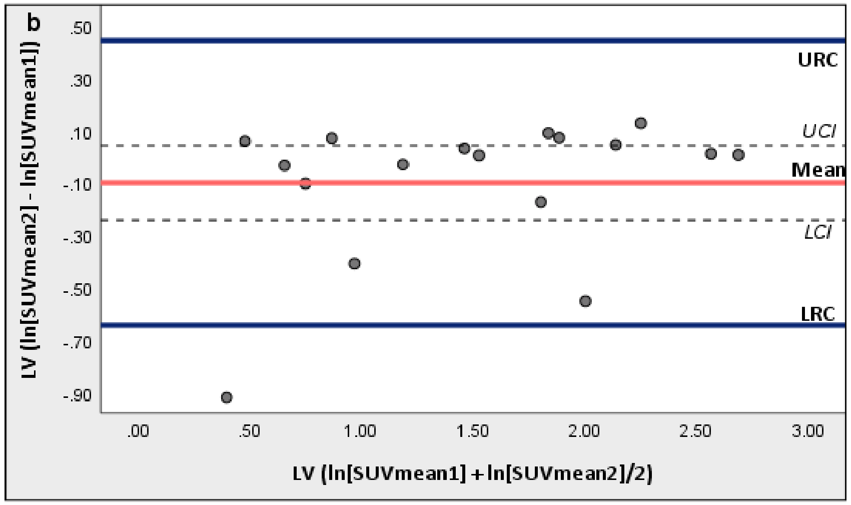

A simple linear regression indicates no significant bias (β = 0.131, p = 0.176).

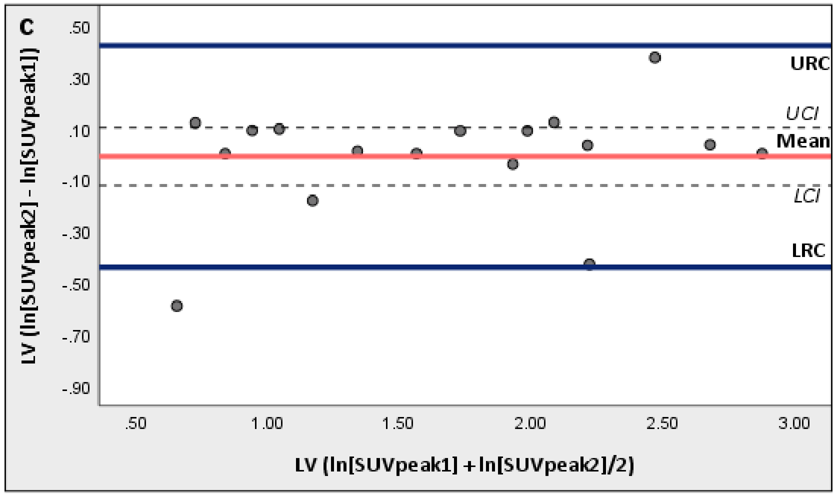

A simple linear regression indicates no significant bias (β = 0.080, p = 0.319).

URC/LRC: upper and lower repeatability coefficients. UCI/LCI: upper and lower 95% confidence intervals for the mean of the difference. SUV: standardized uptake value. LV: left ventricle.

**The 95% RCs for SUVmean may not represent the true limits of agreement because both original and log-transformed differences are not normally distributed at significance level (P < 0.0015).

Fig. S12 Inter-observer repeatability of FDG uptake in left ventricle (LV) illustrated by Bland-Altman method.

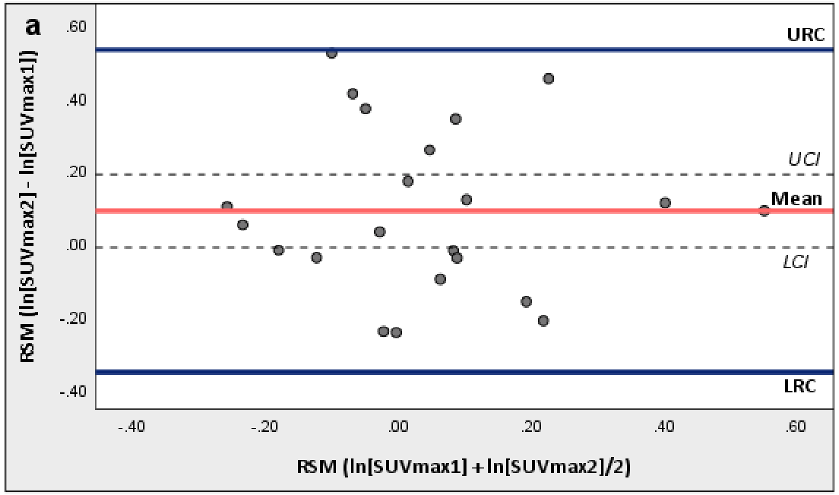

A simple linear regression indicates no significant bias (β = -0.062, *p* = 0.815).

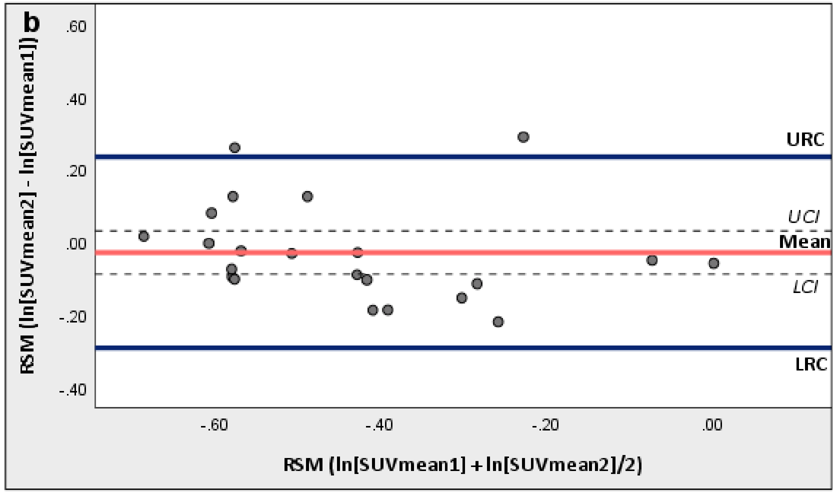

A simple linear regression indicates no significant bias (β = -0.159, *p* = 0.338).

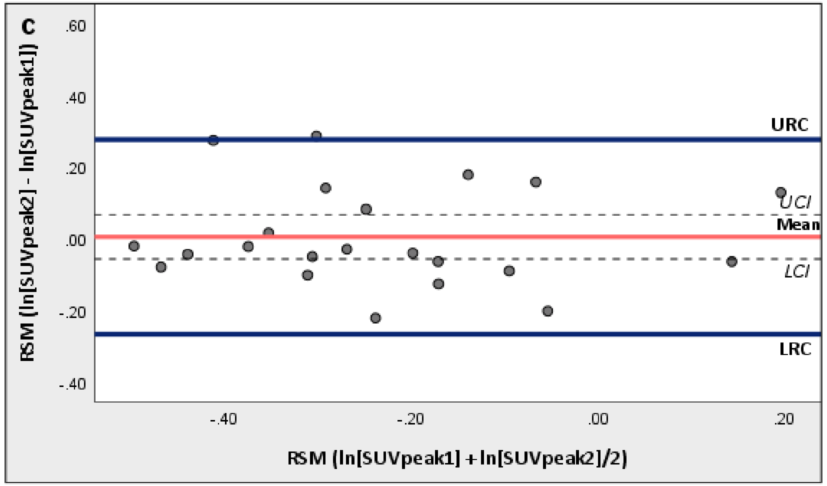

A simple linear regression indicates no significant bias (β = -0.026, *p* = 0.880).

URC/LRC: upper and lower repeatability coefficients. UCI/LCI: upper and lower 95% confidence intervals for the mean of the difference. SUV: standardized uptake value. RSM: right skeletal muscle.

Fig. S13 Inter-observer repeatability of FDG uptake in right skeletal muscle (RSM) illustrated by Bland-Altman method.

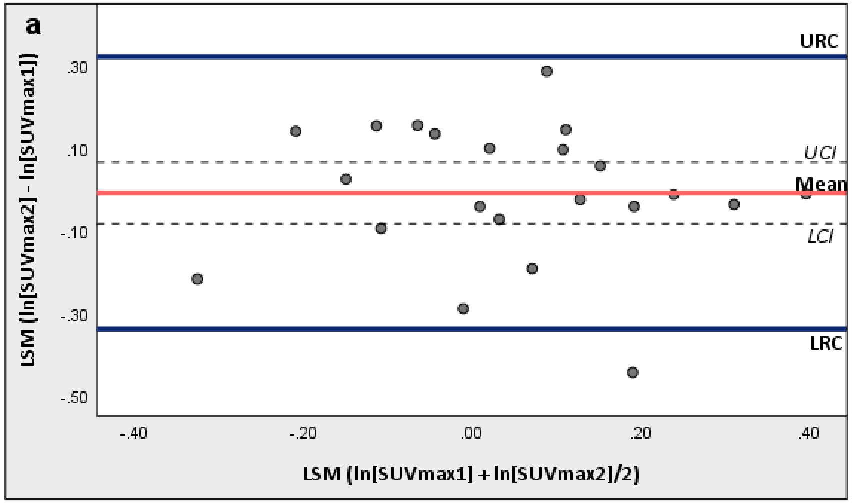

A simple linear regression indicates no significant bias (β = -0.069, *p* = 0.756).

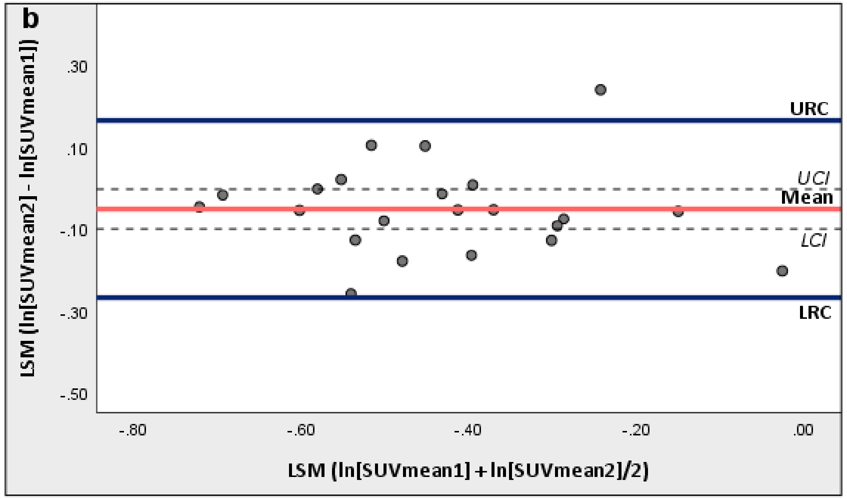

A simple linear regression indicates no significant bias (β = -0.054, *p* = 0.714).

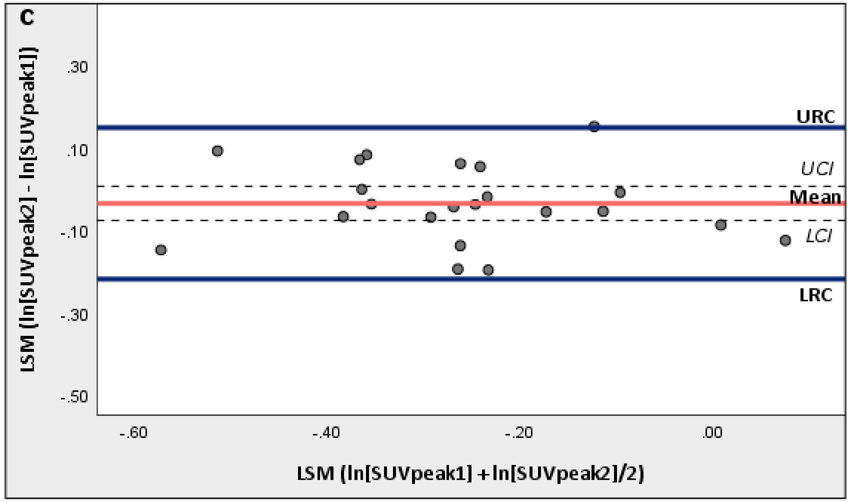

A simple linear regression indicates no significant bias (β = -0.081, *p* = 0.563).

URC/LRC: upper and lower repeatability coefficients. UCI/LCI: upper and lower 95% confidence intervals for the mean of the difference. SUV: standardized uptake value. LSM: left skeletal muscle.

Fig. S14 Inter-observer repeatability of FDG uptake in left skeletal muscle (LSM) illustrated by Bland-Altman method.

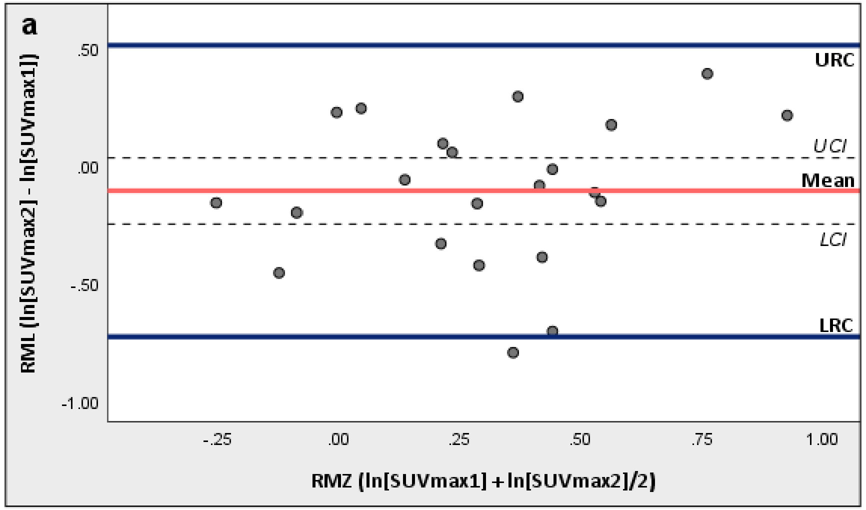

A simple linear regression indicates no significant bias (β = 0.227, p = 0.362).

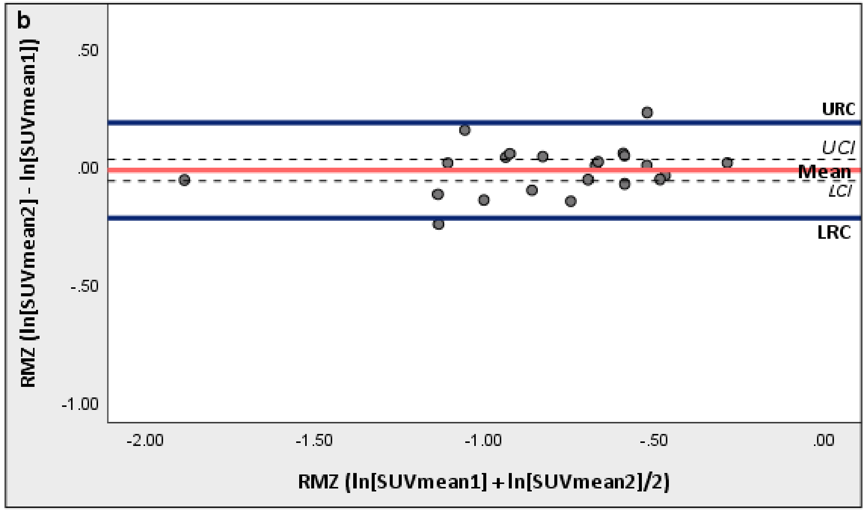

A simple linear regression indicates no significant bias (β = 0.080, p = 0.234).

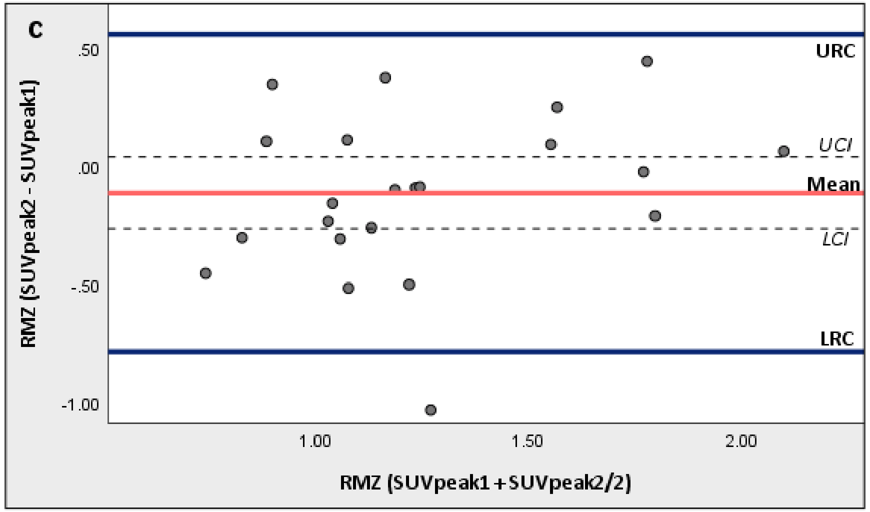

A simple linear regression indicates no significant bias (β = 0.374, p = 0.124).

URC/LRC: upper and lower repeatability coefficients. UCI/LCI: upper and lower 95% confidence intervals for the mean of the difference.
SUV: standardized uptake value. RMZ: right middle lung zone.

Fig. S15 Inter-observer repeatability of FDG uptake in right middle lung zone (RMZ) illustrated by Bland-Altman method.

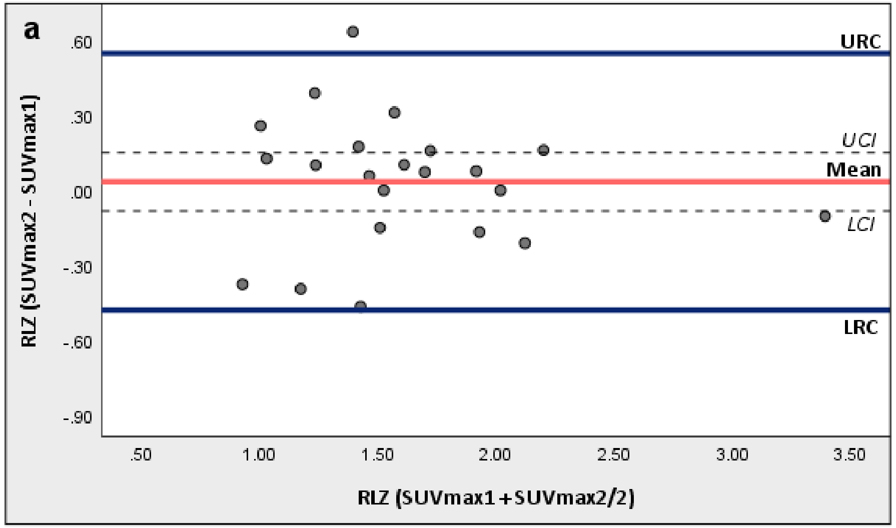

A simple linear regression indicates no significant bias (β = -0.047, p = 0.670) *

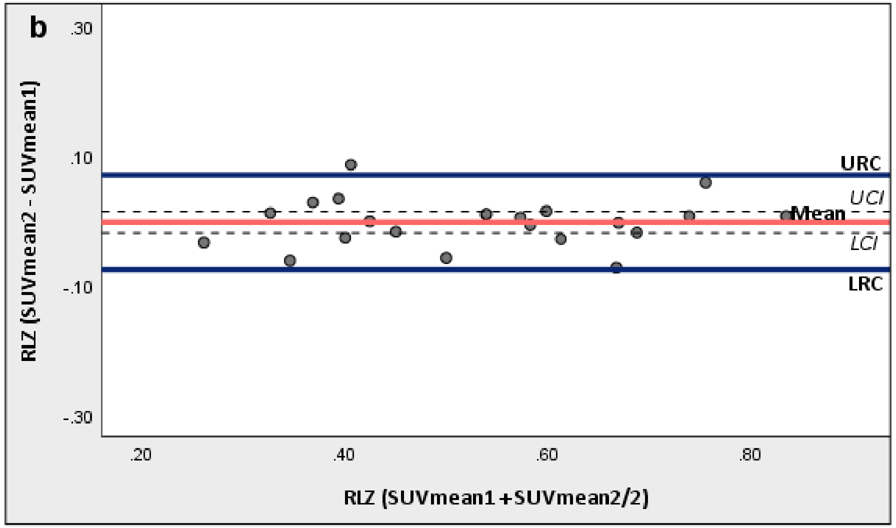

A simple linear regression indicates no significant bias (β = 0.033, p = 0.593).

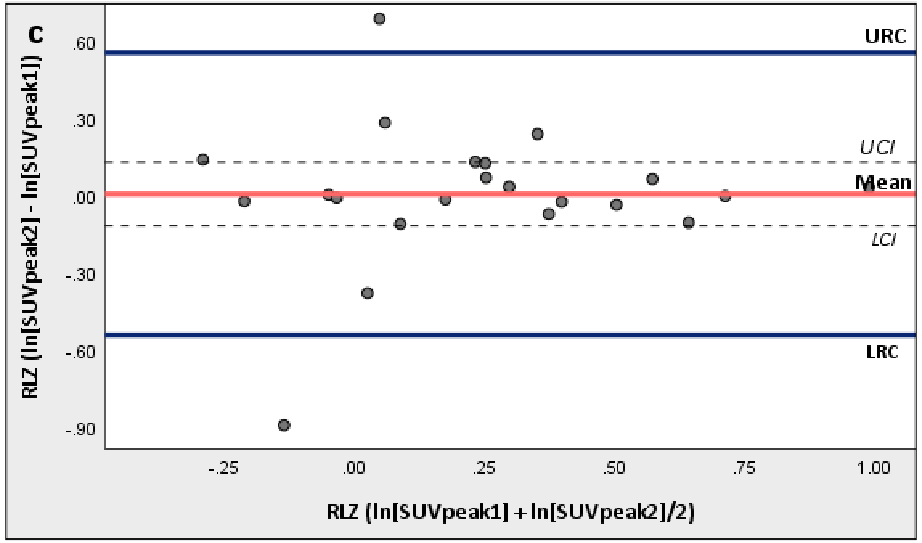

A simple linear regression indicates no significant bias (β = 0.094, p = 0.639) **

URC/LRC: upper and lower repeatability coefficients. UCI/LCI: upper and lower 95% confidence intervals for the mean of the difference.
SUV: standardized uptake value. RLZ: right lower lung zone.

*There is a correlation between the magnitude of the mean log-SUVpeak values and their log-differences based on Kendall τ, thus graph created using the original data.

**The 95% RCs for SUVpeak may not represent the true limits of agreement because the log-transformed difference is not normally distributed at significance level ($p < 0.0015$).

Fig. S16 Inter-observer repeatability of FDG uptake in right lower lung zone (RLZ) illustrated by Bland-Altman method.

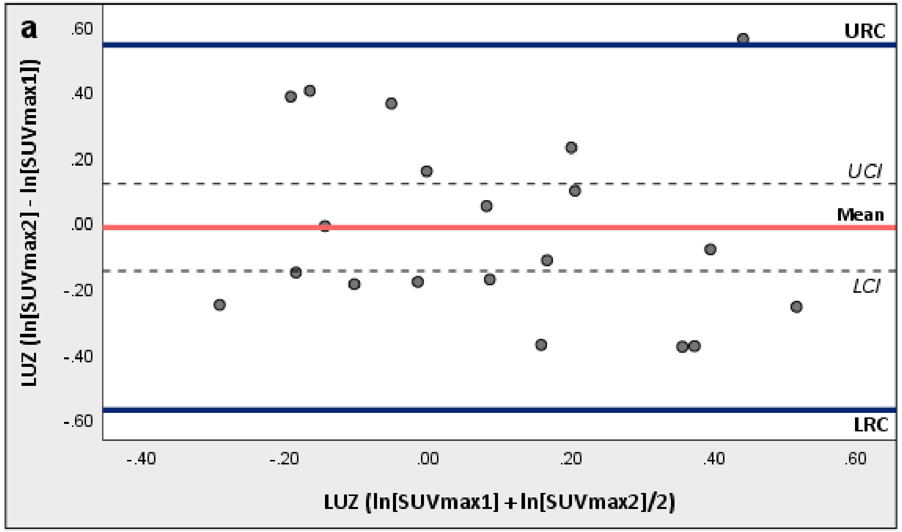

A simple linear regression indicates no significant bias (β = -0.207, *p* = 0.464).

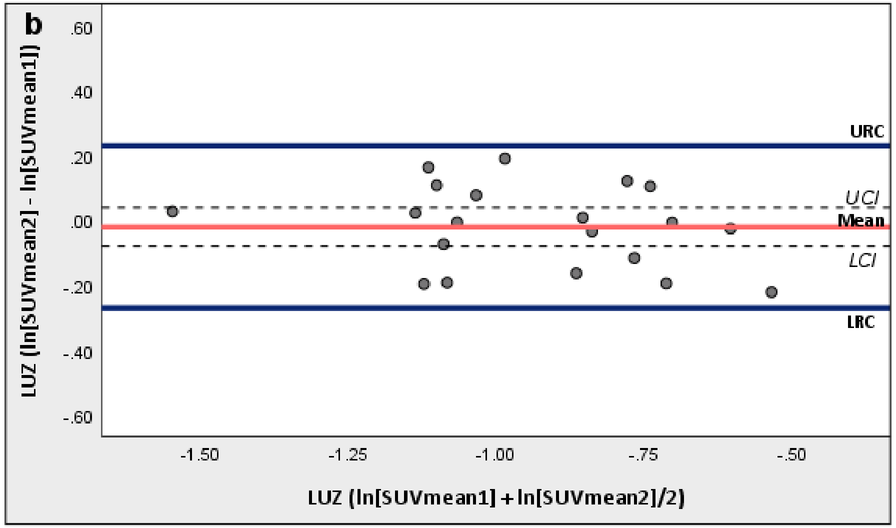

A simple linear regression indicates no significant bias (β = -0.134, *p* = 0.287).

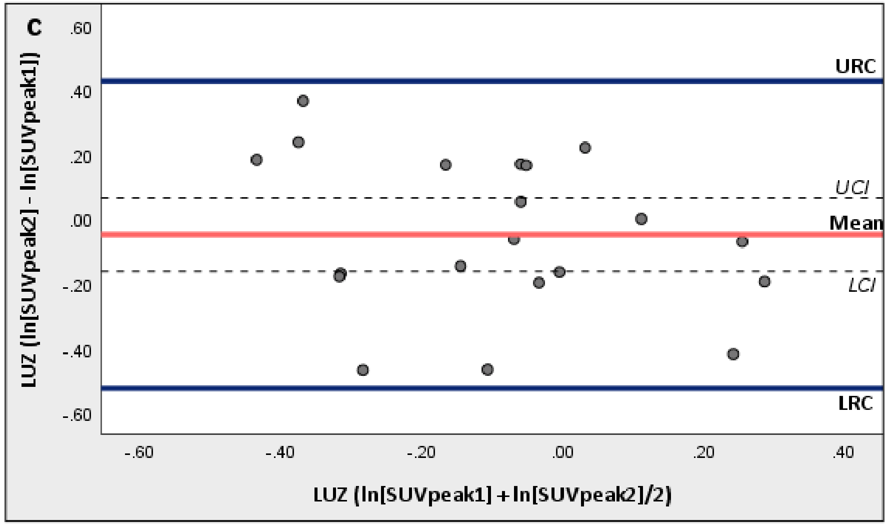

A simple linear regression indicates no significant bias (β = -0.330, *p* = 0.213).

URC/LRC: upper and lower repeatability coefficients. UCI/LCI: upper and lower 95% confidence intervals for the mean of the difference. SUV: standardized uptake value. LUZ: left upper lung zone.

Fig. S17 Inter-observer repeatability of FDG uptake in left upper lung zone (LUZ) illustrated by Bland-Altman method.

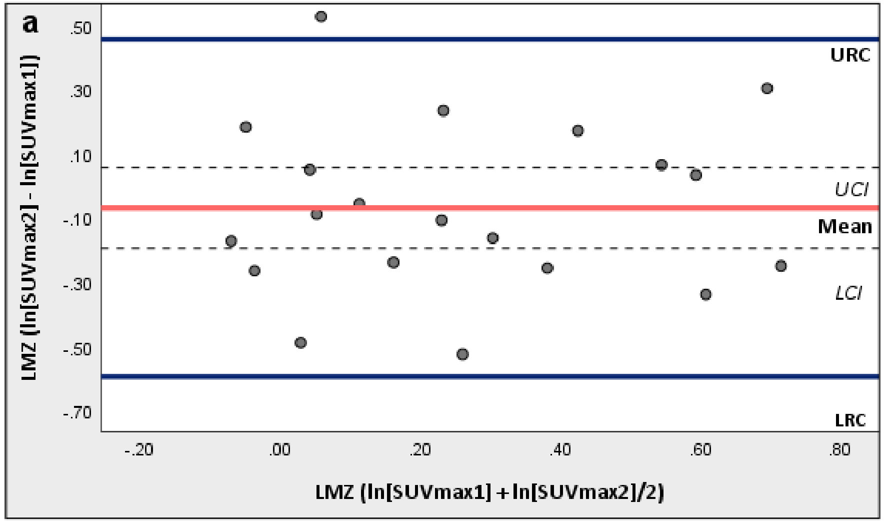

A simple linear regression indicates no significant bias (β = 0.038, p = 0.878).

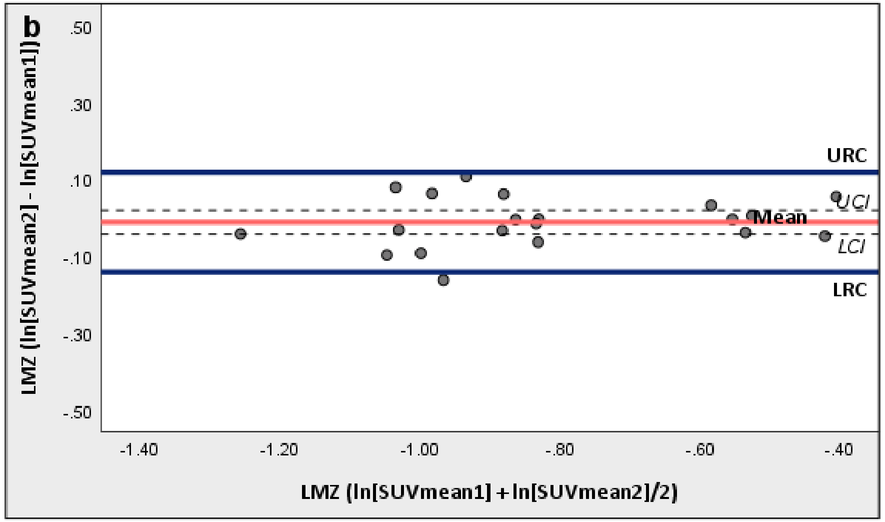

A simple linear regression indicates no significant bias (β = 0.045, p = 0.496).

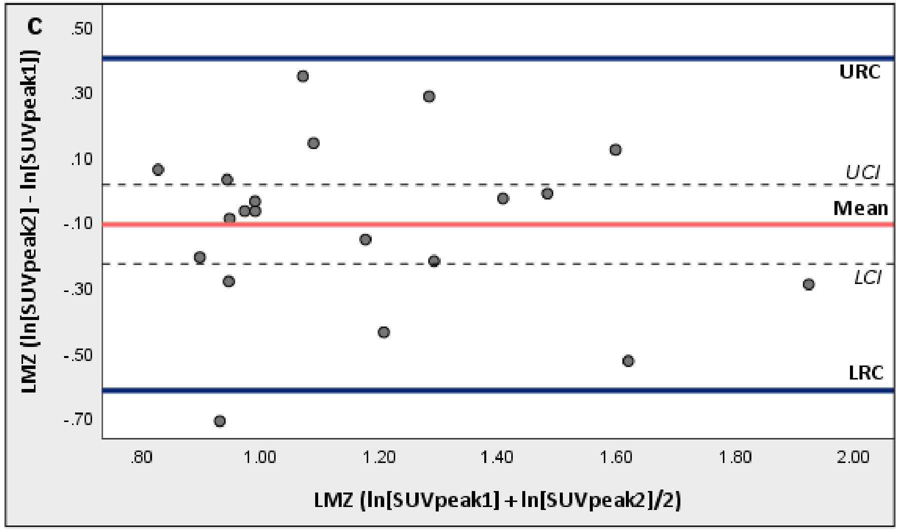

A simple linear regression indicates no significant bias (β = 0.083, p = 0.694).

URC/LRC: upper and lower repeatability coefficients. UCI/LCI: upper and lower 95% confidence intervals for the mean of the difference. SUV: standardized uptake value. LMZ: left middle lung zone.

Fig. S18 Inter-observer repeatability of FDG uptake in left middle lung zone (LMZ) illustrated by Bland-Altman method.

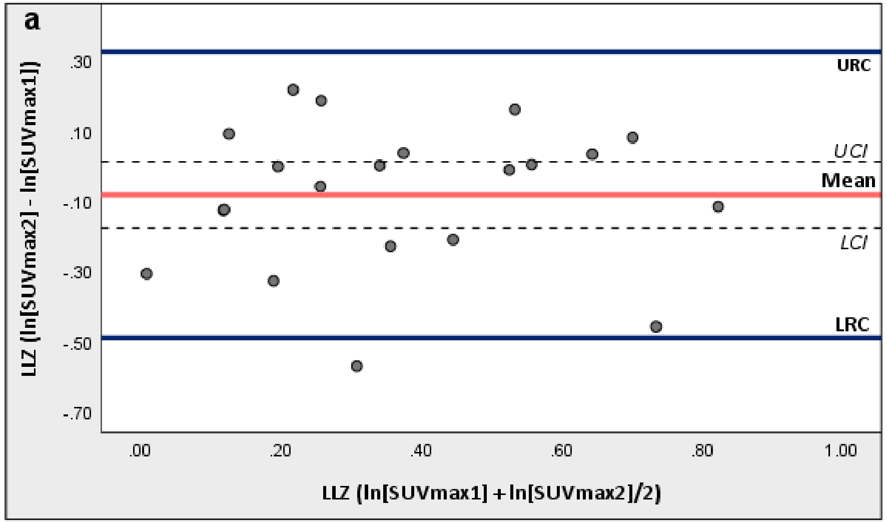

A simple linear regression indicates no significant bias (β = 0.032, *p* = 0.881).

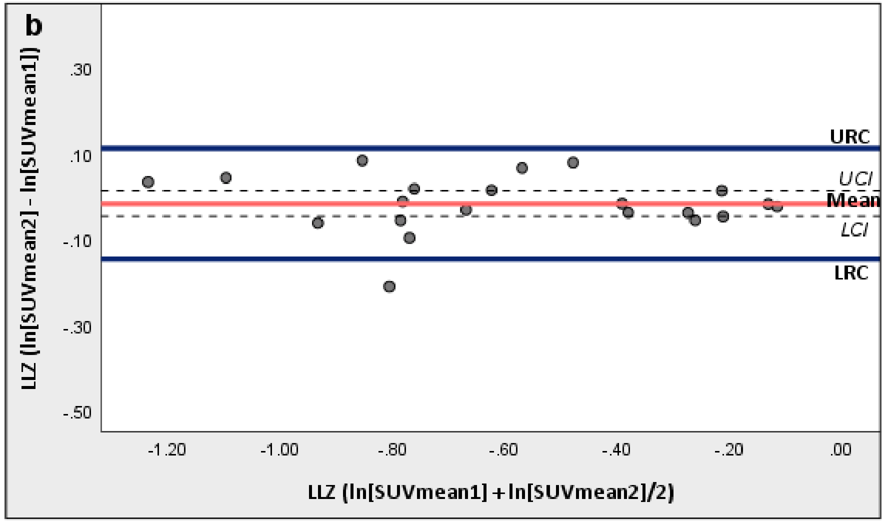

A simple linear regression indicates no significant bias (β = -0.016, *p* = 0.742).

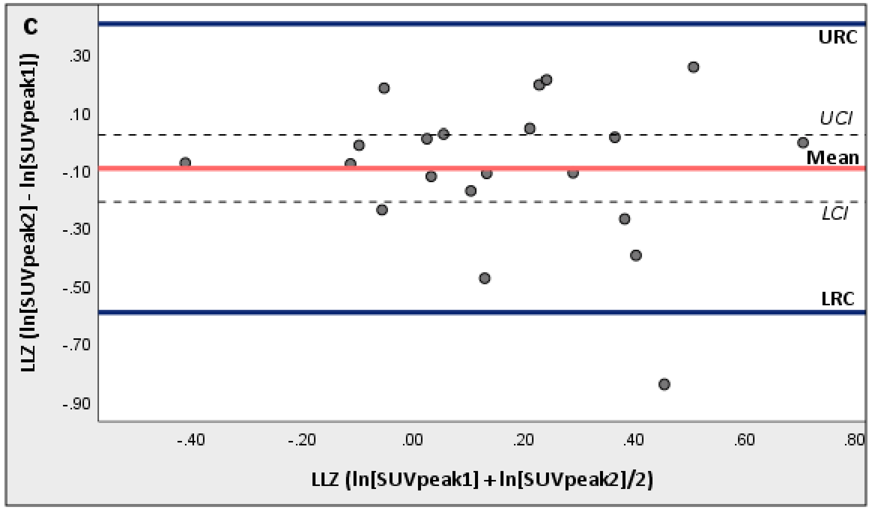

A simple linear regression indicates no significant bias (β = -0.124, *p* = 0.593).

URC/LRC: upper and lower repeatability coefficients. UCI/LCI: upper and lower 95% confidence intervals for the mean of the difference. SUV: standardized uptake value. LLZ: left lower lung zone.

Fig. S19 Inter-observer repeatability of FDG uptake in left lower lung zone (LLZ) illustrated by Bland-Altman method.

Fig. S20 Distribution of different SUV parameters including maximum, mean and peak for inter-observer measurements in each organ:

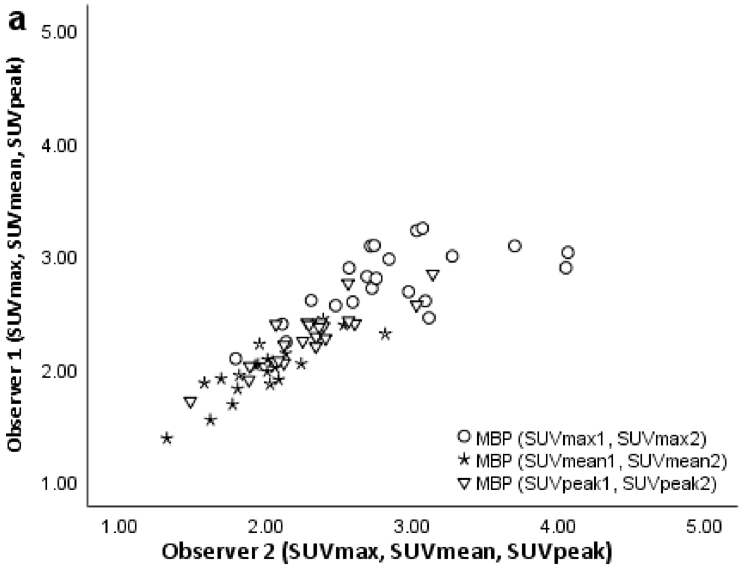

SUVmax, SUVmean and SUVpeak distribution in inter-observer test of mediastinal blood pool (MBP)

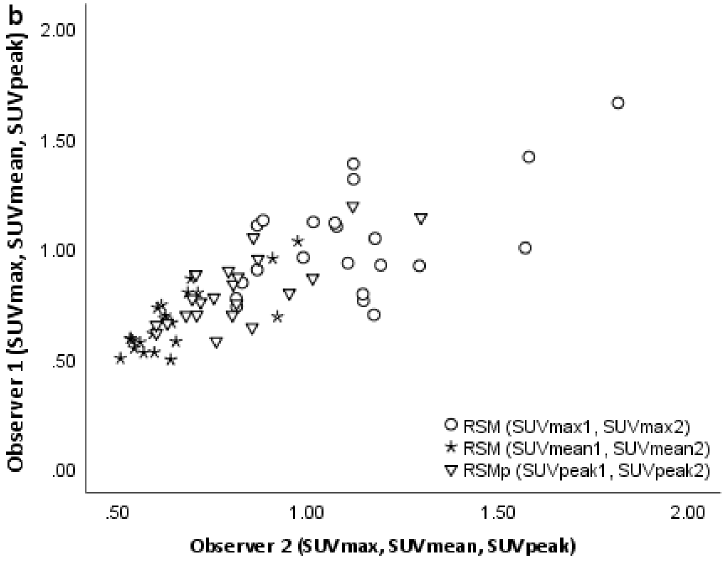

SUVmax, SUVmean and SUVpeak distribution in inter-observer test of right skeletal muscle (RSM)

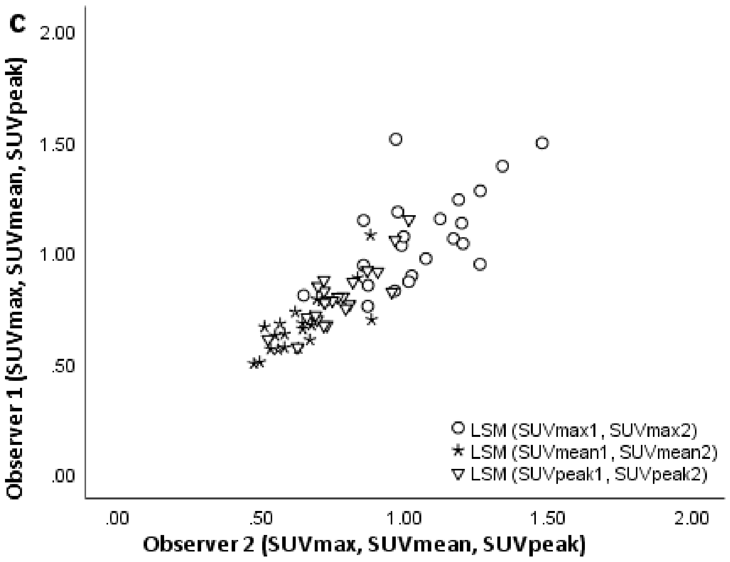

SUVmax, SUVmean and SUVpeak distribution in inter-observer test of left skeletal muscle (LSM)

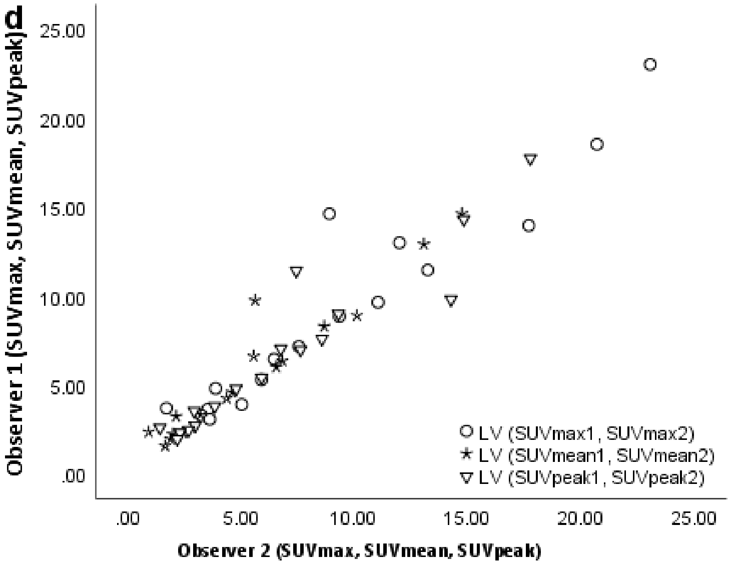

SUVmax, SUVmean and SUVpeak distribution in inter-observer test of left ventricle (LV)

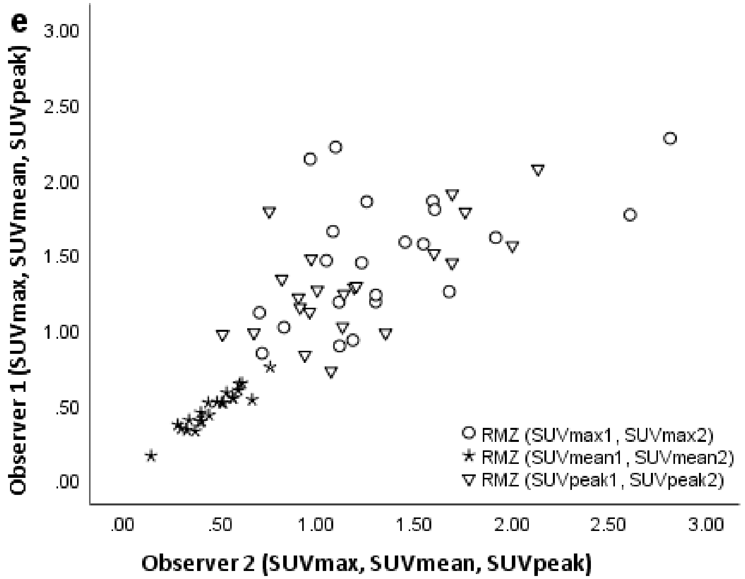

SUVmax, SUVmean and SUVpeak distribution in inter-observer test of right middle lung zone (RMZ)

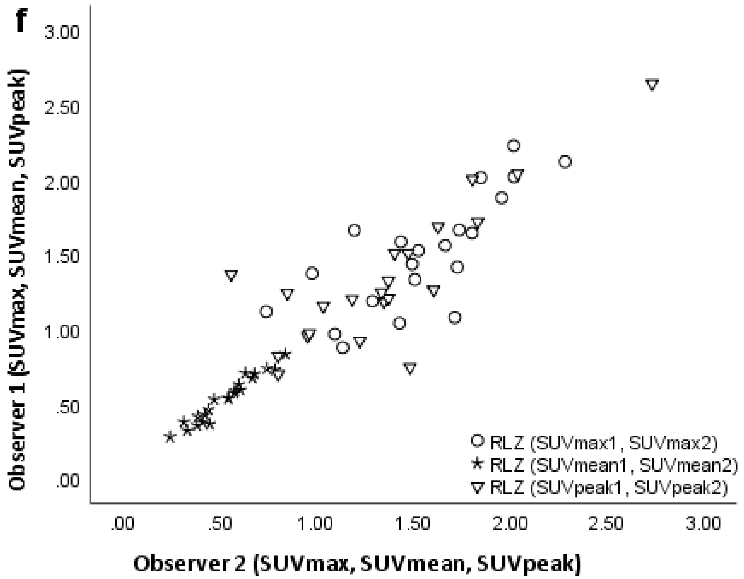

SUVmax, SUVmean and SUVpeak distribution in inter-observer test of right lower lung zone (RLZ)

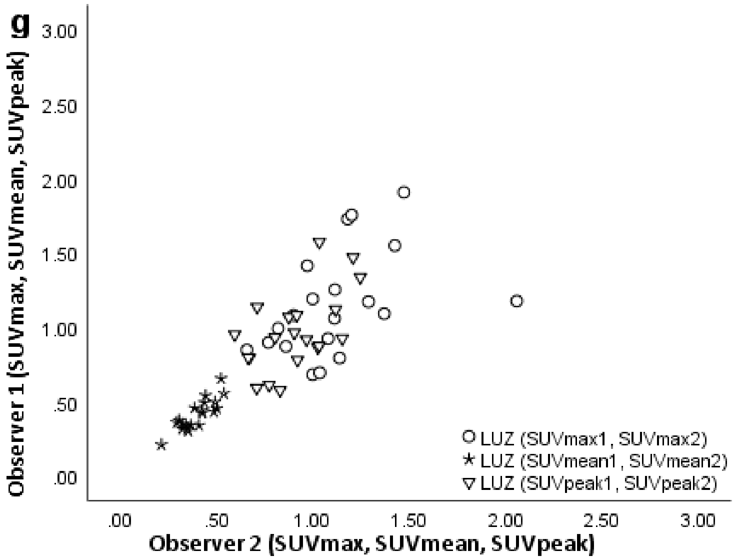

SUVmax, SUVmean and SUVpeak distribution in inter-observer test of left upper lung zone (LUZ)

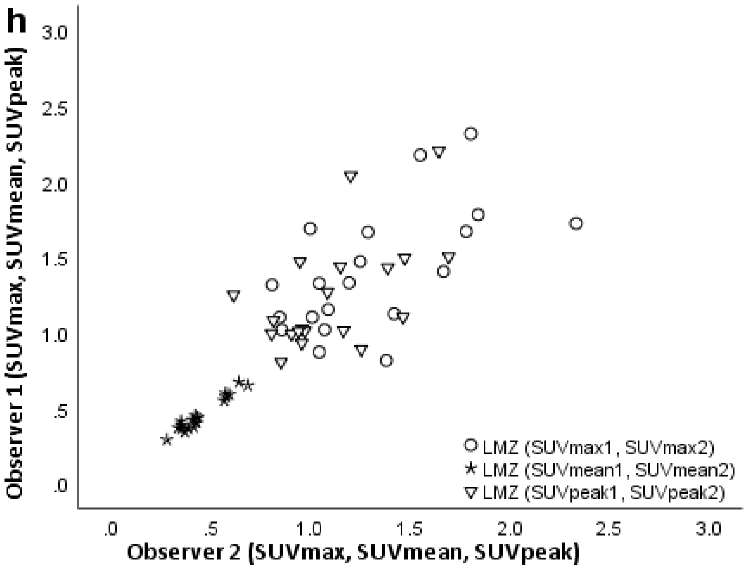

SUVmax, SUVmean and SUVpeak distribution in inter-observer test of left middle lung zone (LMZ)

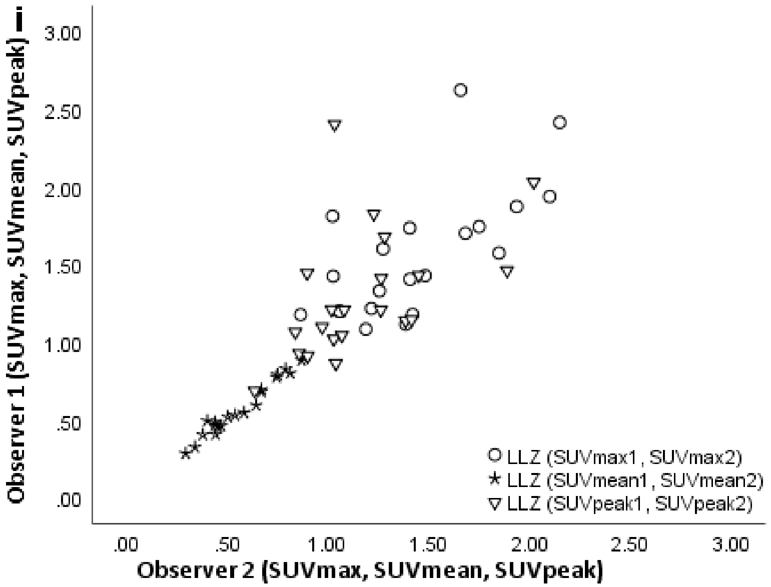

SUVmax, SUVmean and SUVpeak distribution in inter-observer test of left lower lung zone (LLZ)
